# The association between basal metabolic rate and ischemic stroke: a Mendelian randomization study

**DOI:** 10.3389/fneur.2025.1434740

**Published:** 2025-03-03

**Authors:** Yizhou Chen, Xiahui Zhang, Meifang Liu, Yi Zhang, Song Li, Li Zhou, Xiaolin Yang, Xu Chen, Mengqi Yue, Qi Qu, Yong Qiu, Jing Shi

**Affiliations:** ^1^Yunnan University of Traditional Chinese Medicine, Kunming, China; ^2^Department of Acupuncture and Moxibustion, First Affiliated Hospital of Yunnan University of Traditional Chinese Medicine, Kunming, China; ^3^Department of Acupuncture and Moxibustion, Yunnan Provincial Hospital of Traditional Chinese Medicine, Kunming, China; ^4^Qingdao Central Hospital, Qingdao, China; ^5^Department of Medicine, Hubei Minzu University, Enshi, China

**Keywords:** basal metabolic rate, ischemic stroke, causal relationship, Mendelian randomization, MR analysis

## Abstract

**Objective:**

This study aims to elucidate the potential impact of basal metabolic rate on ischemic stroke at the genetic prediction level through a two-sample Mendelian randomization analysis.

**Methods:**

Using summary data from genome-wide association studies, we obtained information on basal metabolic rate and ischemic stroke from a large-scale genome-wide association study. MR analysis used inverse variance weighting, weighted median, MR-Egger, simple mode, and weighted estimation. Sensitivity analyses, including the MR-Egger method, MR-PRESSO, Cochran’s *Q*-test, and leave-one-out assessment, were performed to assess the reliability of the results.

**Results:**

Genetic susceptibility to basal metabolic rate was significantly associated with ischemic stroke in multiple models, including the inverse variance weighting model (*OR*, 1.108 [95% CI: 1.005–1.221]; *p* = 0.0392), the weighted median method (*OR*, 1.179 [95% CI: 1.020–1.363]; *p* = 0.0263), and MR-Egger (*OR*, 1.291 [95% CI: 1.002–1.663]; *p* = 0.0491). These results indicate a positive causal relationship between basal metabolic rate and ischemic stroke. The MR-Egger intercept and Cochran’s *Q*-test indicated the absence of heterogeneity and horizontal pleiotropy in the analyses of basal metabolic rate and ischemic stroke.

**Conclusion:**

The MR analysis suggests a positive correlation between basal metabolic rate and ischemic stroke.

## 1 Introduction

Ischemic stroke (IS) accounts for the majority of stroke cases, representing 87% of all occurrences (1). The narrow therapeutic window, the risk of complications, and limited treatment efficacy impose significant constraints, resulting in only a small proportion of patients receiving treatment (2). Therefore, it is particularly important to focus on preventing potential risk factors for IS and reducing its incidence in the early stages.

Basal Metabolic Rate (BMR) is a critical indicator of the minimum metabolic activity necessary to sustain life, and also a significant component of total energy expenditure. An individual’s BMR is primarily influenced by factors such as age, genetic makeup, body weight, environmental temperature, and overall health status (3). High blood pressure is a well-established risk factor for IS, and recent studies have demonstrated that an increase in BMR can lead to elevated blood pressure (4). Furthermore, IS is closely linked to brain energy metabolism (5). Disruption of the brain’s energy metabolism process can exacerbate the condition. Current Mendelian randomization (MR) studies on BMR primarily focus on its causal relationship with cardiovascular diseases and its association with inflammatory markers. Furthermore, a cross-sectional study (6) on stroke patients indicated that individuals with IS had higher BMR levels, though it did not elucidate the causal relationship. Therefore, based on the aforementioned evidence, we raise the question: Is there a causal relationship between BMR and IS?

Understanding the interaction between BMR and IS is essential for revealing the underlying mechanisms. This study not only provides a new perspective for predicting the occurrence of IS but may also offer scientific evidence to reduce its incidence and improve disease management. However, clarifying the causal relationship is challenging due to the intricate interactions among physiological factors. Although randomized controlled trials (RCTs) are considered the gold standard for causal inference, they are complex in design and resource-intensive. Traditional observational studies (7), however, are susceptible to reverse causality, confounding factors, and sample size limitations. In contrast, MR effectively addresses these limitations by leveraging data from genome-wide association studies (GWAS) and using single nucleotide polymorphisms (SNPs) as instrumental variables, offering a more reliable approach to uncovering causal relationships between exposures and clinical outcomes (8).

## 2 Materials and methods

### 2.1 Study design

**Figure 1:**
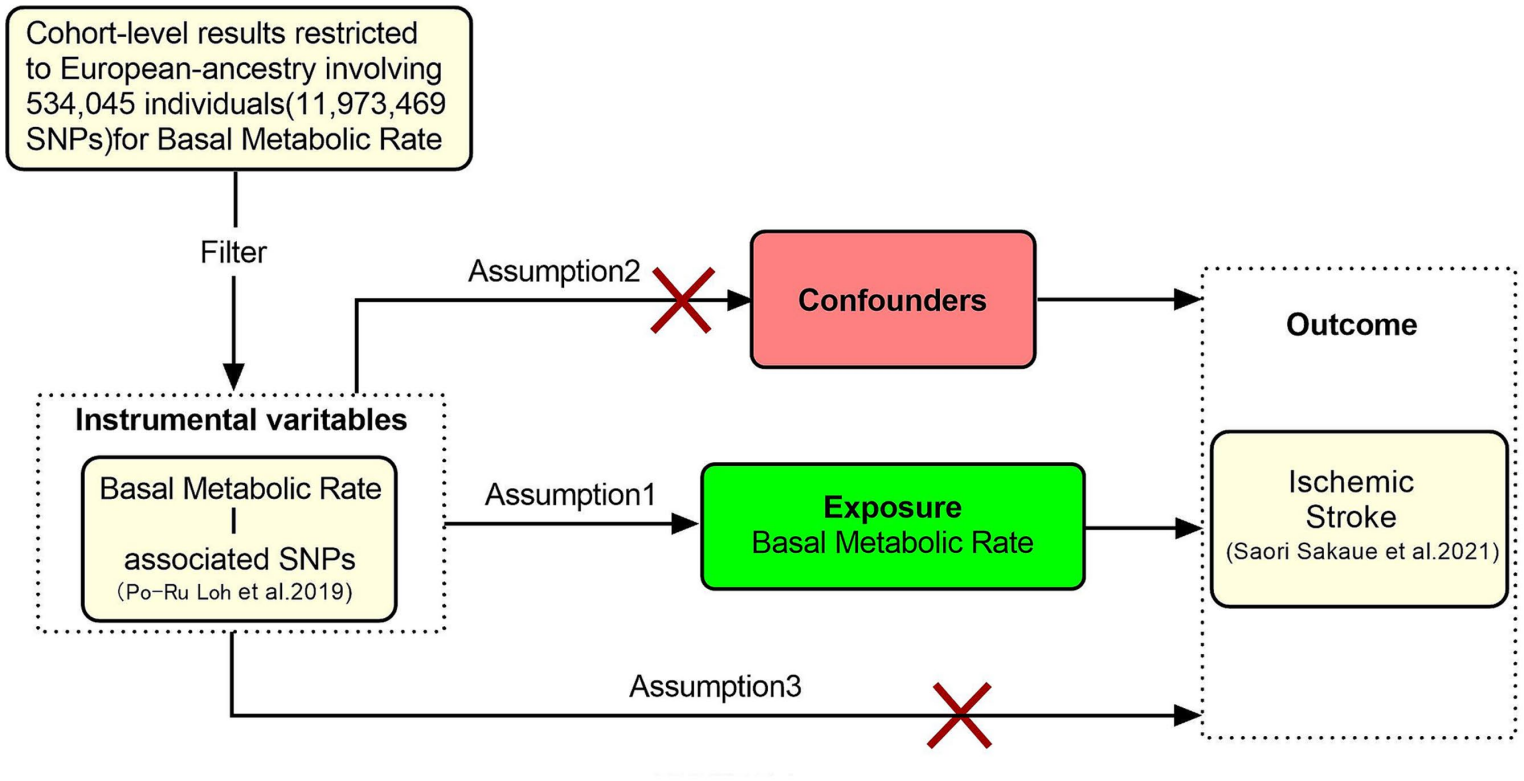
Schematic representation of the MR study.

Mendelian randomization (MR) studies must satisfy three core assumptions: (1) the instrumental variables (IVs) must be strongly associated with the exposure. (2) The IVs must be independent of any confounding factors. (3) The IVs must affect the outcome only through the exposure, with no direct effect on the outcome beyond the exposure (9). This study uses BMR as the exposure variable and IS as the outcome variable. We perform a two-sample MR analysis to investigate the causal relationship between BMR and IS. The specific research methodology is shown in Figure 1.

### 2.2 Data source

The data for BMR and IS were obtained from the GWAS database.^1^ The GWAS dataset for BMR (GWAS ID: ebi-a-GCST90029025) was obtained from the UK Biobank by Po-Ru Loh and colleagues using the BOLT-LMM Bayesian mixed model association method, which resulted in a cohort analysis of 534,045 participants and identified 11,973,469 SNPs (10). Similarly, the GWAS dataset for IS (GWAS ID: ebi-a-GCST90018864) included 484,121 participants (11,929 cases and 472,192 controls) and 9,587,836 SNPs (11). Both datasets focused on European populations to minimize biases from racial differences and are publicly available in the IEU GWAS database. Therefore, no additional ethical approvals are required. Further details on these data can be found in Supplementary Table 1.

### 2.3 Selection of instrumental variables

To satisfy assumption 2, we selected instrumental variables for BMR based on genome-wide significance (*p* < 5e^−8^). Using the clump function from the TwoSampleMR package in R (version 4.3.3), we performed linkage disequilibrium analysis (*r*^2^ < 0.001, window size >10,000 *kb*) to ensure the independence of the instrumental variables. Subsequently, we excluded SNPs with intermediate allele frequencies, palindromic SNPs, and incompatible SNPs from the selection.

To eliminate potential confounding SNPs associated with the outcome variable, we used the PhenoScanner database.^2^ Previous studies have identified risk factors causally associated with IS, including blood lipids (12), blood pressure (13), and type 2 diabetes (14). In this study, these risk factors are regarded as potential confounders. Therefore, we excluded SNPs associated with blood lipid levels, blood pressure (including both diastolic and systolic pressure), and type 2 diabetes. Finally, to ensure the robustness of our analysis, we applied a stringent threshold for statistical strength, setting *F* > 10 as the criterion for strong associations to minimize the potential for weak instrument bias. If *F* < 10, the corresponding SNP should be excluded (15). The formula for calculating the F-statistic is presented below:


$$F=\frac{N-K-1}{K}\times \frac{R^{2}}{1-R^{2}}$$

In this context, *R*^2^ represents the proportion of variance in BMR explained by the SNP, indicating the extent to which the instrumental variable accounts for the exposure (16). *N* represents the sample size of the GWAS, and K denotes the number of instrumental variables.

The selection of IVs in this study adhered to a rigorous step-by-step process based on the outlined methodology. Initially, we used the “TwoSampleMR” package in R to select 598 SNPs for the analysis of IS. Subsequently, 28 palindromic SNPs were excluded from the analysis. Additionally, 48 SNPs were excluded due to their association with potential confounding factors related to IS. Following this, we applied the MR-PRESSO method, which excluded 234 outlier SNPs (Supplementary Table 2). The remaining 288 SNPs were then subjected to F-statistic calculation. The F-statistics for these 288 SNPs ranged from 11.408 to 290.562, with all values exceeding 10, indicating that the potential for weak instrument bias was minimized. The combined *R^2^* value for these 288 SNPs was 2.03%, demonstrating their robustness. Consequently, these 288 SNPs were ultimately included in our study (Supplementary Table 3).

### 2.4 Statistical analysis

In this study, we aim to establish the causal relationship between BMR and IS using several methodologies, including the Inverse-Variance Weighted (IVW) method, the Weighted Median (WME) method, the MR-Egger method, the weighted method, and the simple model. The core concept of the IVW method is to combine the effects of multiple genetic instrumental variables on exposure and outcome, with weights inversely proportional to the estimated variance (17). This weighting approach aims to provide more accurate and reliable results by giving greater weight to instrumental variables with lower standard errors. This method enhances the robustness of the estimation process by reducing the impact of random errors. Pleiotropy occurs when a genetic variant affects multiple phenotypes or biological pathways (18).

In MR studies, pleiotropy in IVs can lead to biased estimates of the causal relationship between exposure and outcome. The MR-Egger regression method is designed to address pleiotropy in instrumental variables, offering a method to detect and correct for bias caused by directional pleiotropy. When assumption 3 of Mendelian randomization is violated, MR-Egger regression is necessary to produce a robust estimate of the causal relationship between exposure and outcome (19). Heterogeneity refers to significant variation in effect estimates among different instrumental variables, which may indicate pleiotropy or other underlying issues. To detect such heterogeneity, we applied the IVW method and MR-Egger regression to identify SNPs with heterogeneity, followed by Cochran’s Q statistic to quantify the heterogeneity (20). Subsequently, we performed sensitivity analyses, such as leave-one-out analysis, to determine whether any individual SNPs significantly influence the primary causal relationship (21). Additionally, when horizontal pleiotropy is below 50%, the MR-PRESSO method is recommended, incorporating the MR-PRESSO global test to detect horizontal pleiotropy. The MR-PRESSO outlier test excludes outlier SNPs and estimates corrected results (18).

The “Two Sample MR” R package was used for all two-sample MR analyses and associated sensitivity tests. The MR-PRESSO analysis was performed using the R package “MR-PRESSO.” Statistical analyses were conducted in R (version 4.3.3), with a significance threshold set at *p* < 0.05 to determine statistical significance.

## 3 Result

### 3.1 MR analysis: influence of the BMR on IS

**Figure 2:**
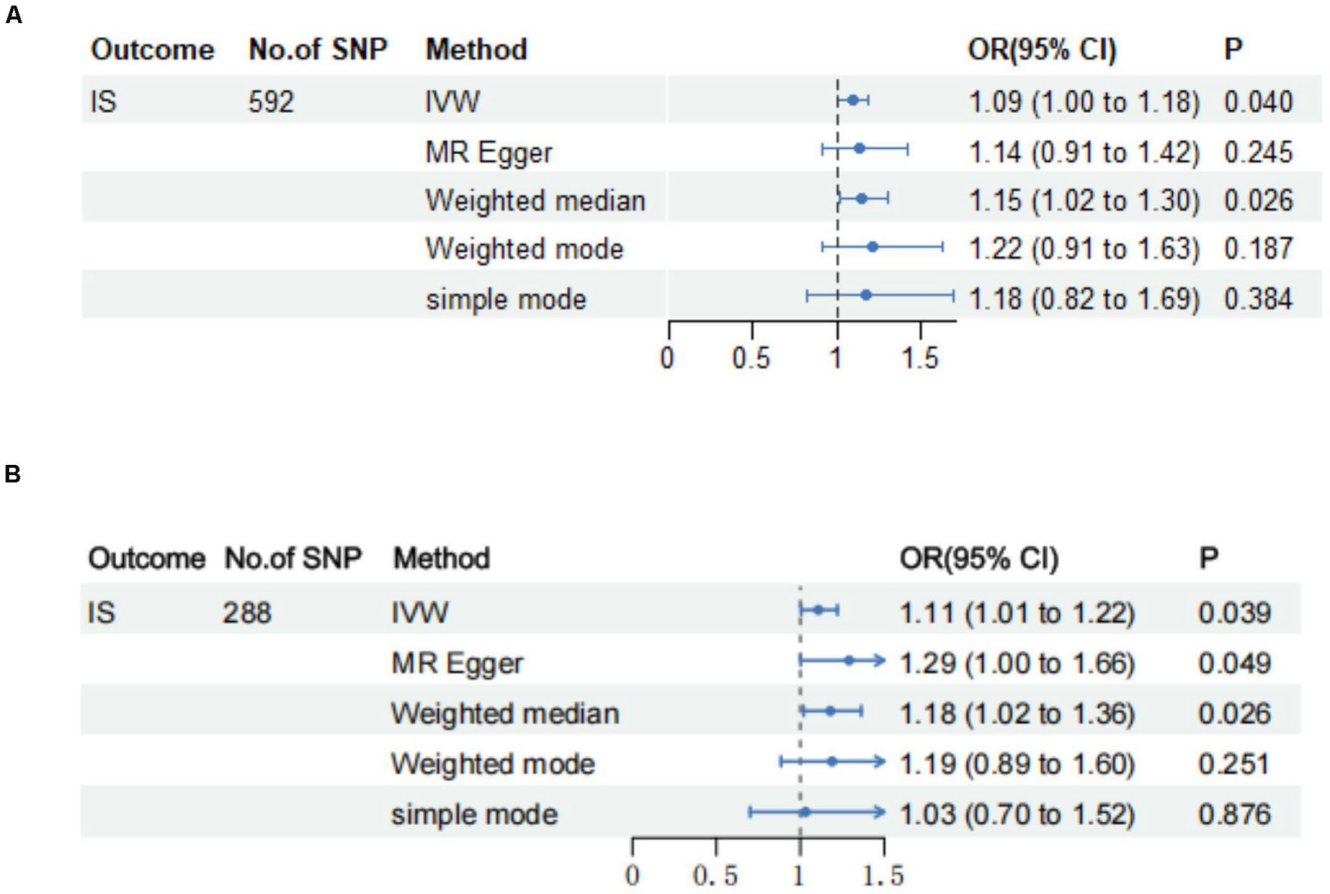
Forest plots of Mendelian randomization analyses of the causal effects of BMR on IS before and after MR-PRESSO. **(A)** Forest plots of Mendelian randomization analyses of the causal effects of BMR on IS at before MR-PRESSO. **(B)** Forest plots of Mendelian randomization analyses of the causal effects of BMR on IS after MR-PRESSO.

We used the IVW method to analyze the relationship between BMR and IS, revealing a potential positive genetic correlation between BMR and the risk of IS (odds ratio [*OR*], 1.090 [95% CI: 1.004–1.182]; *p* = 0.0396). The WME yielded similar results (*OR*, 1.151 [95% CI: 1.018–1.301]; *p* = 0.0244). Nonetheless, upon employing the MR-Egger method for analysis, we did not observe a statistically significant association between BMR and IS (*OR*, 1.139 [95% CI: 0.915–1.418]; *p* = 0.2454) (Supplementary Table 4; Figure 2). After removing 234 outlier SNPs, the MR-Egger intercept showed no evidence of directional pleiotropy (Egger intercept = −0.002, *p* = 0.2010). The MR-PRESSO global test rigorously scrutinized horizontal pleiotropy (*p* < 0.05), providing evidence of no statistically significant directional horizontal pleiotropy in our analysis (Supplementary Table 5). We conducted Cochran’s Q test both before and after removing outliers. Before outlier removal, the results indicated significant heterogeneity (MR-Egger: *Q* value = 598.632, *p* = 0.009; IVW: *Q* value = 598.842, *p* = 0.010) (Supplementary Table 6). Although we observed some heterogeneity in our analysis, the impact on our results was minimal due to the use of a random effects model. After the removal of outliers, our analysis showed no substantial heterogeneity (MR-Egger: *Q* value = 311.501, *p* = 0.144; IVW: *Q* value = 313.293, *p* = 0.1370) (Supplementary Table 6). Finally, we conducted IVW analysis (*OR*, 1.108 [95% CI: 1.005–1.221]; *p* = 0.0392), WME (*OR*, 1.179 [95% CI: 1.020–1.363]; *p* = 0.0263), and MR-Egger (*OR*, 1.291 [95% CI: 1.002–1.663]; *p* = 0.0491) on the final set of 288 SNPs (Supplementary Table 4; Figure 2). All results were statistically significant. The findings from the MR-Egger and weighted median sensitivity analyses supported the conclusions drawn from the IVW analyses, further reinforcing the consistency of the results. Scatter plots and funnel plots are provided in the supplementary figures (Supplementary Figures 1, 2). Additionally, our thorough examination using the leave-one-out analysis (Supplementary Figure 3) failed to detect any specific SNPs that significantly altered the overall relationship between BMR and IS. Thus, based on this comprehensive assessment, we confidently assert that BMR exerts a positive causal influence on IS.

### 3.2 Reverse MR analysis

In this study, we also conducted a reverse MR analysis to assess the causal impact of IS on BMR. The data sources for this analysis were consistent with those mentioned earlier. We initially selected instrumental variables associated with IS, resulting in 14 SNPs (Supplementary Table 7). After performing the MR analysis, we found no significant causal effect of IS on BMR in IVW analysis (*OR*, 1.004 [95% CI: 0.9708–1.0380]; *p* = 0.8224) (Supplementary Table 8).

## 4 Discussion

We conducted MR analysis, and the final results indicated a positive effect of BMR on IS. We also performed various sensitivity analyses to ensure the robustness of our findings. This study found a significant association between BMR and the risk of IS, consistent with previous findings, and further established the causal relationship between BMR and IS. Previous studies have mainly focused on specific populations or short-term analyses, whereas this study strengthens the evidence by validating the robustness of this relationship using large samples and long-term data. However, these results may be influenced by certain confounding factors.

The first potential factor is that an increase in BMR indicates a higher energy demand from the body, including respiratory metabolism, organ function, neural activity, and muscle activity (22, 23). Energy production is closely related to mitochondria, the organelles primarily responsible for adenosine triphosphate (ATP) production. When BMR increases, energy demands rise, leading to a significant increase in mitochondrial workload, which may elevate the risk of mitochondrial dysfunction (24, 25). Furthermore, research has indicated a positive correlation between resting metabolic rate (RMR) and various aspects of mitochondrial function, including small molecule transport across mitochondrial membranes, as well as inner and outer membrane transport and mitochondrial transfer (26). RMR and BMR are closely related, as both describe energy expenditure during rest. Although they differ, they often have similar values and are generally considered equivalent in the study (27, 28). This correlation between BMR and mitochondrial transport suggests that an excessively high BMR may lead to an increase in mitochondrial transport rates, potentially causing decompensation. This decompensation may result in an imbalance in the internal mitochondrial environment, such as disturbances in cellular calcium ion flux, which may disrupt mitochondrial function (29, 30), indirectly indicating that a high BMR may lead to mitochondrial dysfunction.

Mitochondrial dysfunction can lead to several consequences, including energy metabolism disorders, redox imbalance, increased apoptosis, cellular calcium ion imbalance, and disrupted mitochondrial autophagy (31). Recent studies on the cellular and molecular mechanisms associated with IS have suggested that redox imbalance, cellular calcium ion dysregulation, and disruptions in mitochondrial autophagy may be linked to IS (32–34). Interestingly, an MR analysis examining the relationship between BMR and vascular disease risk, based on mitochondrial aging theory, also concluded that increased BMR can lead to a surge in reactive oxygen species (ROS) (35). These findings are consistent with our results.

An increase in ROS primarily results from redox imbalance, leading to enhanced oxidative stress. Calcium ion dysregulation is associated with mitochondrial transport; when mitochondrial transport increases, cellular calcium ion concentrations tend to rise, potentially increasing the risk of IS. Mitochondrial autophagy is a crucial physiological process, and its disruption can trigger a cascade of adverse effects (36). A potential mechanism underlying these effects could be that the pathogenesis of IS involves oxidative stress and inflammatory responses. Disruption of mitochondrial autophagy could exacerbate oxidative stress, leading to excess ROS production and increased release of inflammatory cytokines (34, 37). These pieces of evidence suggest that the causal relationship between BMR and IS may be driven by underlying mitochondrial dysfunction, warranting further exploration of the biological mechanisms involved in future studies.

Thyroid-stimulating hormone (TSH) levels may play a crucial role in the observed association between BMR and IS. Research indicates that thyroid hormone secretion is correlated with BMR levels and plays a critical role in regulating basal metabolic rate (38). As BMR increases, the hypothalamus reduces the release of thyrotropin-releasing hormone (TRH), which subsequently lowers TSH release, leading to a decrease in thyroid hormone production (39). This physiological process is regulated by the hypothalamic–pituitary-thyroid (HPT) axis. A reduction in TSH levels can increase the likelihood of hyperthyroidism (39, 40). An MR study investigating the impact of thyroid function on stroke risk through atrial fibrillation, along with another study examining stroke risk within the normal thyroid function range, found a significant association between higher TSH levels within the normal range and lower stroke risk, potentially mediated by atrial fibrillation (41, 42). Elevated BMR may increase the risk of IS due to its effect on TSH levels.

Furthermore, thrombosis is a direct pathological cause of IS (43). An MR study suggested that higher BMR may increase the risk of venous thrombosis in the lower limbs, potentially through BMR-induced endothelial dysfunction, which creates a pro-inflammatory and pro-coagulant environment in the vascular system (44). Although IS is caused by arterial thrombosis in the brain, the underlying mechanisms in the vascular system are similar. Therefore, we hypothesize that the causal relationship between BMR and IS may be mediated by thrombosis, and further investigation is needed to confirm the link between BMR and cerebral blood vessels.

The results of this study suggest a potential causal relationship between high BMR levels and IS, indicating that elevated BMR could serve as an early risk marker for IS. Monitoring individual BMR levels, especially in populations with metabolic abnormalities, could help identify high-risk individuals early, enabling timely intervention. This provides a new perspective for clinical practice and stroke prevention strategies. Furthermore, future studies could investigate whether lifestyle changes or pharmacological treatments can help individuals with elevated BMR maintain it within the normal range, potentially aiding in the prevention of IS. If successful, these approaches could expand clinical strategies for stroke prevention and open new avenues for reducing stroke risk in individuals with elevated BMR.

This study presents an MR analysis of the relationship between BMR and IS, providing new insights into early stroke prevention for individuals with elevated BMR. While numerous studies have explored BMR and cardiovascular health, research specifically investigating BMR and IS is still lacking, making this study innovative in its focus. Furthermore, the use of MR analysis reduces the impact of confounding factors and biases, enabling more robust causal inferences.

Despite these strengths, our study has several limitations. First, the GWAS data used in this analysis were derived from European populations, raising uncertainty about the applicability of the results to non-European populations. Second, although IS was used as the outcome measure, we did not conduct subgroup analyses by gender, age group, or geographic region due to the limitations of the GWAS database, which limits our ability to assess the stability of outcomes across different subgroups. Third, due to the inability to extract data on hemorrhagic stroke, our analysis was restricted to IS.

## 5 Conclusion

In conclusion, our research findings suggest that a higher BMR may increase the risk of IS. Intervention measures aimed at improving BMR may be beneficial in reducing the incidence of IS.

## Data Availability

The datasets presented in this study can be found in online repositories. The names of the repository/repositories and accession number(s) can be found in the article/Supplementary material.

## Footnotes

^1^ http://gwas-api.mrcieu.ac.uk/

^2^ http://www.phenoscanner.medschl.cam.ac.uk/

## References

[1] Feske SK. Ischemic stroke. Am J Med. (2021) 134:1457–64. doi: 10.1016/j.amjmed.2021.07.027, PMID: 34454905

[2] Powers WJ Rabinstein AA Ackerson T. Guidelines for the early Management of Patients with Acute Ischemic Stroke: 2019 update to the 2018 guidelines for the early Management of Acute Ischemic Stroke: a guideline for healthcare professionals from the American Heart Association/American Stroke Association. Stroke. (2019) 50:e344–418. doi: 10.1161/STR.0000000000000211, PMID: 31662037

[3] Bi X Forde CG Goh AT. Basal metabolic rate and body composition predict habitual food and macronutrient intakes: gender differences. Nutrients. (2019) 11:2653. doi: 10.3390/nu11112653, PMID: 31689964 PMC6893862

[4] Ali N Mahmood S Manirujjaman M. Hypertension prevalence and influence of basal metabolic rate on blood pressure among adult students in Bangladesh. BMC Public Health. (2017) 18:58. doi: 10.1186/s12889-017-4617-9, PMID: 28743284 PMC5526296

[5] Sifat AE Nozohouri S Archie SR. Brain energy metabolism in ischemic stroke: effects of smoking and diabetes. Int J Mol Sci. (2022) 23:8512. doi: 10.3390/ijms23158512, PMID: 35955647 PMC9369264

[6] Wilczyński J Mierzwa-Molenda M Habik-Tatarowska N. Differences in body composition among Patientsafter hemorrhagic and ischemic stroke. Int J Environ Res Public Health. (2020) 17:4170. doi: 10.3390/ijerph17114170, PMID: 32545352 PMC7312185

[7] Smith GD Ebrahim S. Mendelian randomization: prospects, potentials, and limitations. Int J Epidemiol. (2004) 33:30–42. doi: 10.1093/ije/dyh132, PMID: 15075143

[8] Bowden J Holmes MV. Meta-analysis and Mendelian randomization: a review. Res Synth Methods. (2019) 10:486–96. doi: 10.1002/jrsm.1346, PMID: 30861319 PMC6973275

[9] Richmond RC Davey SG. Mendelian randomization: concepts and scope. Cold Spring Harb Perspect Med. (2022) 12:a040501. doi: 10.1101/cshperspect.a040501, PMID: 34426474 PMC8725623

[10] Loh PR Kichaev G Gazal S. Mixed-model association for biobank-scale datasets. Nat Genet. (2018) 50:906–8. doi: 10.1038/s41588-018-0144-6, PMID: 29892013 PMC6309610

[11] Sakaue S Kanai M Tanigawa Y. A cross-population atlas of genetic associations for 220 human phenotypes. Nat Genet. (2021) 53:1415–24. doi: 10.1038/s41588-021-00931-x, PMID: 34594039 PMC12208603

[12] Bétrisey S Haller ML Efthimiou O. Lipid-lowering therapy and risk of hemorrhagic stroke: a systematic review and Meta-analysis of randomized controlled trials. J Am Heart Assoc. (2024) 13:e030714. doi: 10.1161/JAHA.123.030714, PMID: 38323514 PMC11010101

[13] Buonacera A Stancanelli B Malatino L. Stroke and hypertension: an appraisal from pathophysiology to clinical practice. Curr Vasc Pharmacol. (2019) 17:72–84. doi: 10.2174/1570161115666171116151051, PMID: 29149815

[14] van Sloten TT Sedaghat S Carnethon MR. Cerebral microvascular complications of type 2 diabetes: stroke, cognitive dysfunction, and depression. Lancet Diabetes Endocrinol. (2020) 8:325–36. doi: 10.1016/S2213-8587(19)30405-X, PMID: 32135131 PMC11044807

[15] Burgess S Thompson SG CRP CHD Genetics Collaboration. Avoiding bias from weak instruments in Mendelian randomization studies. Int J Epidemiol. (2011) 40:755–64. doi: 10.1093/ije/dyr036, PMID: 21414999

[16] Pierce BL Burgess S. Efficient design for Mendelian randomization studies: subsample and 2-sample instrumental variable estimators. Am J Epidemiol. (2013) 178:1177–84. doi: 10.1093/aje/kwt084, PMID: 23863760 PMC3783091

[17] Brion MJ Shakhbazov K Visscher PM. Calculating statistical power in Mendelian randomization studies. Int J Epidemiol. (2013) 42:1497–501. doi: 10.1093/ije/dyt179, PMID: 24159078 PMC3807619

[18] Verbanck M Chen CY Neale B. Detection of widespread horizontal pleiotropy in causal relationships inferred from Mendelian randomization between complex traits and diseases. Nat Genet. (2018) 50:693–8. doi: 10.1038/s41588-018-0099-7, PMID: 29686387 PMC6083837

[19] Burgess S Thompson SG. Interpreting findings from Mendelian randomization using the MR-egger method. Eur J Epidemiol. (2017) 32:377–89. doi: 10.1007/s10654-017-0255-x, PMID: 28527048 PMC5506233

[20] Sanderson E Smith GD Windmeijer F. Corrigendum to: an examination of multivariable Mendelian randomization in the single-sample and two-sample summary data settings. Int J Epidemiol. (2020) 49:1057. doi: 10.1093/ije/dyaa101, PMID: 32529219 PMC7394940

[21] Nolte IM. Metasubtract: an R-package to analytically produce leave-one-out meta-analysis GWAS summary statistics. Bioinformatics (Oxford, England). (2020) 36:4521–2. doi: 10.1093/bioinformatics/btaa570, PMID: 32696040 PMC7750933

[22] Genoud M Isler K Martin RD. Comparative analyses of basal rate of metabolism in mammals: data selection does matter. Biol Rev Camb Philos Soc. (2018) 93:404–38. doi: 10.1111/brv.12350, PMID: 28752629

[23] Dahmen N Tonn P Messroghli L. Basal metabolic rate in narcoleptic patients. Sleep. (2009) 32:962–4. doi: 10.1093/sleep/32.7.962 PMID: 19639760 PMC2706907

[24] Zong WX Rabinowitz JD White E. Mitochondria and Cancer. Mol Cell. (2016) 61:667–76. doi: 10.1016/j.molcel.2016.02.011, PMID: 26942671 PMC4779192

[25] Chen W Zhao H Li Y. Mitochondrial dynamics in health and disease: mechanisms and potential targets. Signal Transduct Target Ther. (2023) 8:333. doi: 10.1038/s41392-023-01547-9, PMID: 37669960 PMC10480456

[26] Dubé JJ Collyer ML Trant S. Decreased mitochondrial dynamics is associated with insulin resistance, metabolic rate, and fitness in African Americans. J Clin Endocrinol Metab. (2020) 105:1210–20. doi: 10.1210/clinem/dgz272, PMID: 31833547 PMC7067552

[27] Alazzam AM Alrubaye MW Goldsmith JA. Trends in measuring BMR and RMR after spinal cord injury: a comprehensive review. Br J Nutr. (2023) 130:1720–31. doi: 10.1017/S0007114523000831, PMID: 37092679 PMC10587382

[28] Jéquier E Acheson K Schutz Y. Assessment of energy expenditure and fuel utilization in man. Annu Rev Nutr. (1987) 7:187–208. doi: 10.1146/annurev.nu.07.070187.001155, PMID: 3300732

[29] Nakanishi T Kawasaki Y Nakamura Y. An implication of the mitochondrial carrier SLC25A3 as an oxidative stress modulator in NAFLD. Exp Cell Res. (2023) 431:113740. doi: 10.1016/j.yexcr.2023.113740, PMID: 37557977

[30] Bertero E Maack C. Calcium signaling and reactive oxygen species in mitochondria. Circ Res. (2018) 122:1460–78. doi: 10.1161/CIRCRESAHA.118.310082, PMID: 29748369

[31] Sorrentino V Menzies KJ Auwerx J. Repairing mitochondrial dysfunction in disease. Annu Rev Pharmacol Toxicol. (2018) 58:353–89. doi: 10.1146/annurev-pharmtox-010716-104908, PMID: 28961065

[32] Orellana-Urzúa S Rojas I Líbano L. Pathophysiology of ischemic stroke: role of oxidative stress. Curr Pharm Des. (2020) 26:4246–60. doi: 10.2174/1381612826666200708133912, PMID: 32640953

[33] Ludhiadch A Sharma R Muriki A. Role of calcium homeostasis in ischemic stroke: a review. CNS Neurol Disord Drug Targets. (2022) 21:52–61. doi: 10.2174/1871527320666210212141232, PMID: 33583386

[34] Cai Y Yang E Yao X. FUNDC1-dependent mitophagy induced by tPA protects neurons against cerebral ischemia-reperfusion injury. Redox Biol. (2021) 38:101792. doi: 10.1016/j.redox.2020.101792, PMID: 33212415 PMC7679257

[35] Ning L He C Lu C. Association between basal metabolic rate and cardio-metabolic risk factors: evidence from a Mendelian randomization study. Heliyon. (2024) 10:e28154. doi: 10.1016/j.heliyon.2024.e28154, PMID: 38590845 PMC10999873

[36] Goodall EA Kraus F Harper JW. Mechanisms underlying ubiquitin-driven selective mitochondrial and bacterial autophagy. Mol Cell. (2022) 82:1501–13. doi: 10.1016/j.molcel.2022.03.012, PMID: 35364016 PMC9254164

[37] Tan Z Dong F Wu L. Transcutaneous electrical nerve stimulation (TENS) alleviates brain ischemic injury by regulating neuronal oxidative stress, Pyroptosis, and Mitophagy. Mediat Inflamm. (2023) 2023:1–13. doi: 10.1155/2023/5677865, PMID: 37101593 PMC10125764

[38] Kim B. Thyroid hormone as a determinant of energy expenditure and the basal metabolic rate. Thyroid. (2008) 18:141–4. doi: 10.1089/thy.2007.0266, PMID: 18279014

[39] Mullur R Liu YY Brent GA. Thyroid hormone regulation of metabolism. Physiol Rev. (2014) 94:355–82. doi: 10.1152/physrev.00030.2013, PMID: 24692351 PMC4044302

[40] Silva JE. Thyroid hormone control of thermogenesis and energy balance. Thyroid. (1995) 5:481–92. doi: 10.1089/thy.1995.5.481, PMID: 8808101

[41] Marouli E Kus A Del Greco MF. Thyroid function affects the risk of stroke via atrial fibrillation: a Mendelian randomization study. J Clin Endocrinol Metab. (2020) 105:2634–41. doi: 10.1210/clinem/dgaa239, PMID: 32374820 PMC7316221

[42] Chaker L Baumgartner C den Elzen WPJ. Thyroid function within the reference range and the risk of stroke: an individual participant data analysis. J Clin Endocrinol Metab. (2016) 101:4270–82. doi: 10.1210/jc.2016-2255, PMID: 27603906 PMC5095234

[43] Hankey GJ. Secondary stroke prevention. Lancet Neurol. (2014) 13:178–94. doi: 10.1016/S1474-4422(13)70255-2, PMID: 24361114

[44] Huang J Xie Y. Genetically predicted basal metabolic rate and venous thromboembolism risk: a Mendelian randomization study. Front Nutr. (2023) 10:1263804. doi: 10.3389/fnut.2023.1263804, PMID: 38188880 PMC10768029

