# Supplementary material for: The association between basal metabolic rate and ischemic stroke: a Mendelian randomization study
Source: Front Neurol. 2025 Mar 3;16:1434740. doi: 10.3389/fneur.2025.1434740 (PMC11912940; doi:10.3389/fneur.2025.1434740)
Supplement: Supplementary file 4 [file Table_1.DOCX]

| **Phenotype** | **GWAS ID** | **Consortium** | **Simple Size** | **Number of SNPs** | **Population** |
| --- | --- | --- | --- | --- | --- |
| BMR | ebi-a-GCST90029025 | NA | 534,045 | 11,973,469 | European |
| IS | ebi-a-GCST90018864 | NA | 484,121 | 24,174,314 | European |
| Abbreviations:BMR, Basal Metabolic mass; IS,Ischemic Stroke. | | | | | |

**Supplementary Table 1 Overview of the data sources of the exposures and outcome used in the MR Study.**
