# Supplementary material for: The association between basal metabolic rate and ischemic stroke: a Mendelian randomization study
Source: Front Neurol. 2025 Mar 3;16:1434740. doi: 10.3389/fneur.2025.1434740 (PMC11912940; doi:10.3389/fneur.2025.1434740)
Supplement: Supplementary file 5 [file Table_2.DOCX]

| **Supplementary Table 2 Exclusion of IS Outliers Detected in MR-PRESSO** | | | | | | | | |
| --- | --- | --- | --- | --- | --- | --- | --- | --- |
| N | SNP | EA | beta.exp | se.exp | pval.exp | beta.out | se.out | pval.out |
| 1 | rs10124197 | C | -0.00708775 | 0.00132487 | 4.00E-08 | 0.006 | 0.0149 | 0.6892 |
| 2 | rs10215645 | C | 0.00793283 | 0.00122103 | 7.70E-11 | -0.0068 | 0.0093 | 0.462 |
| 3 | rs10243988 | A | -0.00909182 | 0.00153622 | 1.70E-08 | 0.0235 | 0.0124 | 0.0572902 |
| 4 | rs1035607 | C | 0.00898685 | 0.00141981 | 3.40E-10 | 9.00E-04 | 0.0089 | 0.9227 |
| 5 | rs1060967 | C | -0.00816335 | 0.00126479 | 2.30E-11 | -0.0025 | 0.0096 | 0.7907 |
| 6 | rs10769939 | T | -0.00911419 | 0.00128219 | 7.20E-13 | -0.0076 | 0.0087 | 0.3837 |
| 7 | rs10773172 | A | 0.0173355 | 0.00138851 | 1.60E-36 | -0.0188 | 0.0111 | 0.0909097 |
| 8 | rs10803955 | G | -0.010769 | 0.00120505 | 3.20E-19 | 0.0052 | 0.0093 | 0.5784 |
| 9 | rs10811668 | A | -0.00879786 | 0.00151909 | 2.50E-09 | -0.0139 | 0.01 | 0.1652 |
| 10 | rs10835368 | C | 0.00910497 | 0.00125782 | 1.70E-13 | 0.0085 | 0.0091 | 0.3533 |
| 11 | rs10958478 | T | 0.0180477 | 0.00145531 | 8.60E-36 | -0.003 | 0.0104 | 0.7738 |
| 12 | rs10973198 | C | -0.00775301 | 0.00121899 | 9.70E-11 | 0.0061 | 0.0086 | 0.4804 |
| 13 | rs10979612 | C | 0.0179176 | 0.00233872 | 1.90E-15 | 0.0022 | 0.0131 | 0.8699 |
| 14 | rs10985962 | T | -0.00720236 | 0.00127069 | 7.00E-09 | -0.0084 | 0.0089 | 0.3448 |
| 15 | rs10995319 | C | -0.00865181 | 0.0014387 | 1.60E-09 | 0.0019 | 0.0121 | 0.8726 |
| 16 | rs111365325 | T | -0.0133059 | 0.00144045 | 2.20E-20 | -0.0033 | 0.0118 | 0.777 |
| 17 | rs11142705 | A | -0.00798757 | 0.00124142 | 1.20E-10 | -0.0126 | 0.0085 | 0.139 |
| 18 | rs1122848 | G | -0.00724958 | 0.00130665 | 1.70E-08 | -0.0266 | 0.0095 | 0.00517297 |
| 19 | rs11263862 | T | 0.00794387 | 0.00131018 | 3.40E-09 | -0.008 | 0.0086 | 0.3561 |
| 20 | rs112685832 | A | 0.014217 | 0.00191907 | 1.00E-13 | 0.001 | 0.0226 | 0.9663 |
| 21 | rs113115311 | A | -0.0128855 | 0.00236902 | 2.90E-08 | -0.0061 | 0.0147 | 0.676401 |
| 22 | rs114177791 | T | 0.0113071 | 0.00148728 | 1.70E-14 | -0.0149 | 0.0168 | 0.3763 |
| 23 | rs11498196 | T | 0.00791775 | 0.00124142 | 2.10E-10 | -0.0219 | 0.0085 | 0.01037 |
| 24 | rs11524516 | A | -0.010174 | 0.00133659 | 2.20E-15 | 0.0194 | 0.0124 | 0.1179 |
| 25 | rs11593107 | T | -0.00792108 | 0.00122311 | 5.60E-10 | 0.0161 | 0.0086 | 0.061 |
| 26 | rs11616283 | C | 0.0113507 | 0.00178826 | 1.20E-10 | -0.0123 | 0.0211 | 0.5593 |
| 27 | rs116337081 | T | 0.0136505 | 0.00237141 | 3.70E-09 | -0.0219 | 0.0292 | 0.4536 |
| 28 | rs11663903 | A | 0.00717217 | 0.00123663 | 1.90E-09 | -0.0089 | 0.0199 | 0.6552 |
| 29 | rs1167309 | T | -0.0104944 | 0.00129452 | 2.00E-16 | -0.0047 | 0.0087 | 0.592899 |
| 30 | rs11712872 | A | 0.0170954 | 0.00189283 | 2.50E-19 | 0.0132 | 0.016 | 0.4089 |
| 31 | rs11739036 | A | -0.0117189 | 0.00131851 | 3.50E-18 | 0.0025 | 0.01 | 0.8011 |
| 32 | rs117451679 | G | 0.0133981 | 0.00200104 | 3.80E-11 | -0.0086 | 0.0213 | 0.685001 |
| 33 | rs1176314 | G | -0.00704636 | 0.00123396 | 5.60E-09 | -0.001 | 0.0084 | 0.907 |
| 34 | rs11771928 | A | -0.00749689 | 0.00133365 | 4.10E-09 | -0.0166 | 0.0121 | 0.1712 |
| 35 | rs11773731 | G | 0.0116689 | 0.00126071 | 2.30E-20 | 0.016 | 0.0086 | 0.0611604 |
| 36 | rs11784985 | T | -0.00834207 | 0.00124208 | 3.60E-12 | -0.0066 | 0.0112 | 0.552699 |
| 37 | rs11833839 | T | 0.0169207 | 0.00262949 | 1.10E-10 | -0.0012 | 0.0124 | 0.9225 |
| 38 | rs11925245 | G | -0.0107224 | 0.00158489 | 8.00E-12 | -0.0107 | 0.0091 | 0.2403 |
| 39 | rs11997525 | A | 0.0135707 | 0.00163554 | 1.90E-16 | 0.0164 | 0.0103 | 0.113 |
| 40 | rs12031493 | G | -0.00829079 | 0.00121424 | 2.90E-12 | 0.0085 | 0.0088 | 0.3298 |
| 41 | rs12041740 | A | -0.0155671 | 0.00137651 | 1.70E-29 | -0.0046 | 0.0101 | 0.6524 |
| 42 | rs12091972 | C | 0.0222206 | 0.00211016 | 3.30E-26 | -0.0097 | 0.0156 | 0.5346 |
| 43 | rs12148418 | A | -0.0104214 | 0.001224 | 3.60E-18 | 0.0014 | 0.0085 | 0.8696 |
| 44 | rs12188627 | G | -0.0107869 | 0.00121316 | 4.20E-19 | -0.0243 | 0.0102 | 0.01689 |
| 45 | rs12213070 | A | -0.00930116 | 0.00127337 | 3.80E-13 | -0.0074 | 0.0091 | 0.4153 |
| 46 | rs12227680 | A | 0.00764283 | 0.00137898 | 3.90E-08 | 0.002 | 0.0147 | 0.8912 |
| 47 | rs12434837 | T | -0.0116772 | 0.00197247 | 1.10E-08 | 0.0134 | 0.0154 | 0.3867 |
| 48 | rs12520308 | C | -0.00907289 | 0.0012978 | 9.80E-13 | -0.0052 | 0.0087 | 0.5514 |
| 49 | rs12520894 | C | 0.00667023 | 0.00121938 | 9.10E-09 | -0.0112 | 0.0111 | 0.3146 |
| 50 | rs12533548 | G | 0.00729168 | 0.00127963 | 8.10E-09 | 1.00E-04 | 0.0091 | 0.9937 |
| 51 | rs12543207 | T | -0.00982666 | 0.00143741 | 2.10E-12 | -0.0048 | 0.0087 | 0.5768 |
| 52 | rs12588830 | A | 0.0100055 | 0.00157846 | 1.40E-10 | 0.0056 | 0.009 | 0.5331 |
| 53 | rs12657771 | A | -0.0117786 | 0.00122852 | 6.80E-22 | -0.0026 | 0.0085 | 0.7621 |
| 54 | rs12679359 | T | -0.0122436 | 0.00177134 | 2.70E-12 | 0.0033 | 0.0094 | 0.7278 |
| 55 | rs12729817 | G | -0.00869965 | 0.00120729 | 1.80E-12 | 0.0205 | 0.0084 | 0.01439 |
| 56 | rs12784071 | T | 0.00898176 | 0.00144751 | 1.40E-09 | 0.0164 | 0.0136 | 0.2282 |
| 57 | rs12881375 | A | -0.00709574 | 0.00134994 | 3.10E-08 | -0.0037 | 0.0095 | 0.696899 |
| 58 | rs12900800 | A | -0.00900092 | 0.0014203 | 8.10E-11 | 0.0196 | 0.0151 | 0.1941 |
| 59 | rs12908182 | T | -0.0102749 | 0.0012226 | 3.40E-16 | 0.0128 | 0.0085 | 0.1319 |
| 60 | rs12959994 | A | -0.0115206 | 0.00146637 | 1.80E-15 | -0.0031 | 0.0093 | 0.736801 |
| 61 | rs12981554 | G | -0.00891311 | 0.00123404 | 8.20E-13 | -0.0055 | 0.0087 | 0.5281 |
| 62 | rs13007086 | T | 0.0362174 | 0.0015995 | 7.80E-116 | 0.003 | 0.0127 | 0.8129 |
| 63 | rs13077747 | C | 0.0131083 | 0.0012139 | 4.60E-27 | -0.0177 | 0.0087 | 0.0407803 |
| 64 | rs13099930 | G | -0.00864195 | 0.00128509 | 1.70E-11 | -0.0026 | 0.0104 | 0.8061 |
| 65 | rs13125807 | T | 0.0113994 | 0.00174859 | 1.50E-11 | 0.003 | 0.0106 | 0.7756 |
| 66 | rs13173394 | C | 0.00736701 | 0.00125897 | 5.90E-09 | -0.0025 | 0.0085 | 0.7651 |
| 67 | rs13176855 | T | -0.00645232 | 0.00123293 | 4.60E-08 | 0.0132 | 0.0099 | 0.1832 |
| 68 | rs13244614 | A | 0.0116781 | 0.00134935 | 1.20E-18 | 0.0246 | 0.0116 | 0.0332698 |
| 69 | rs13299559 | T | -0.00949077 | 0.00122514 | 4.50E-14 | 0.0167 | 0.0113 | 0.1397 |
| 70 | rs13392666 | A | 0.00805374 | 0.00145575 | 1.80E-08 | -0.0024 | 0.0158 | 0.8797 |
| 71 | rs1374370 | A | 0.00892898 | 0.00131109 | 1.60E-11 | -0.0062 | 0.0123 | 0.616601 |
| 72 | rs139031896 | C | -0.021306 | 0.003723 | 7.10E-09 | 0.0105 | 0.0433 | 0.8085 |
| 73 | rs1421035 | T | 0.00768281 | 0.00128788 | 1.60E-09 | -0.0086 | 0.0087 | 0.3219 |
| 74 | rs1424371 | T | 0.00682779 | 0.00127261 | 2.90E-08 | 0.0026 | 0.0085 | 0.763199 |
| 75 | rs1431663 | C | -0.0104291 | 0.00123388 | 2.70E-17 | -0.0078 | 0.0138 | 0.570401 |
| 76 | rs144260843 | A | -0.0231757 | 0.00401798 | 5.90E-09 | 0.0565 | 0.0507 | 0.2655 |
| 77 | rs1517248 | G | -0.00757752 | 0.00127431 | 3.60E-09 | 0.0052 | 0.014 | 0.7089 |
| 78 | rs1539110 | T | 0.00817826 | 0.00141399 | 1.90E-09 | -0.0236 | 0.0155 | 0.1276 |
| 79 | rs1553065 | A | -0.00767302 | 0.00122929 | 2.40E-10 | -0.009 | 0.0087 | 0.2971 |
| 80 | rs1618407 | C | 0.0118004 | 0.00156053 | 2.90E-15 | 0.007 | 0.0091 | 0.4436 |
| 81 | rs1657222 | A | 0.00850909 | 0.00124828 | 1.30E-11 | -0.0017 | 0.0084 | 0.8417 |
| 82 | rs17042011 | T | 0.00918505 | 0.00151809 | 9.00E-10 | 0.0139 | 0.0103 | 0.1762 |
| 83 | rs17197114 | C | 0.0128264 | 0.00161007 | 1.50E-15 | 0.0138 | 0.0148 | 0.3514 |
| 84 | rs1722322 | G | -0.00800814 | 0.00137673 | 2.30E-09 | -0.0115 | 0.0092 | 0.2101 |
| 85 | rs1765133 | T | -0.00984288 | 0.00145352 | 5.10E-11 | -0.0054 | 0.0105 | 0.6084 |
| 86 | rs17708997 | G | 0.0144055 | 0.00193082 | 2.80E-14 | 0.0029 | 0.0179 | 0.8724 |
| 87 | rs17881236 | A | -0.0202624 | 0.00363006 | 4.00E-08 | 0.0033 | 0.0448 | 0.9411 |
| 88 | rs1813212 | G | -0.0084891 | 0.0012263 | 2.60E-12 | 0.0052 | 0.0086 | 0.5406 |
| 89 | rs1846221 | C | -0.00946551 | 0.00162378 | 5.60E-09 | -0.0134 | 0.0156 | 0.3895 |
| 90 | rs1883798 | G | -0.00859234 | 0.00138127 | 1.70E-10 | -0.0286 | 0.0151 | 0.0586003 |
| 91 | rs1910466 | C | -0.00833396 | 0.00121925 | 2.10E-11 | 0.0174 | 0.0084 | 0.0388401 |
| 92 | rs1919442 | G | -0.00867775 | 0.00142386 | 1.20E-09 | 0.0066 | 0.012 | 0.5818 |
| 93 | rs1960268 | T | 0.00782713 | 0.00139994 | 5.10E-09 | 0.0088 | 0.01 | 0.3808 |
| 94 | rs1971955 | G | -0.0128869 | 0.00172333 | 1.70E-14 | -0.0012 | 0.0094 | 0.9001 |
| 95 | rs2025129 | G | -0.00854624 | 0.00143047 | 6.10E-09 | 0.0071 | 0.0131 | 0.584901 |
| 96 | rs2045767 | G | -0.00747892 | 0.00123685 | 4.90E-09 | -0.0149 | 0.0088 | 0.0919306 |
| 97 | rs204928 | G | -0.00834644 | 0.00121828 | 5.20E-12 | -0.0012 | 0.0087 | 0.8947 |
| 98 | rs2065999 | C | -0.00710784 | 0.00125604 | 5.40E-09 | 0.004 | 0.0114 | 0.7258 |
| 99 | rs212538 | C | 0.00785849 | 0.001219 | 6.30E-11 | -0.0056 | 0.0101 | 0.576901 |
| 100 | rs214247 | C | -0.00869719 | 0.00124966 | 1.20E-12 | 0.0173 | 0.0096 | 0.0715007 |
| 101 | rs2163832 | C | -0.0126765 | 0.00129077 | 1.60E-22 | 0.0299 | 0.0147 | 0.0420097 |
| 102 | rs2197563 | A | 0.010305 | 0.00123172 | 2.40E-16 | -0.0311 | 0.0092 | 0.000753095 |
| 103 | rs2197780 | C | 0.0117034 | 0.00134559 | 2.90E-18 | -0.0127 | 0.0094 | 0.1794 |
| 104 | rs2214964 | T | -0.00714483 | 0.00122226 | 2.00E-08 | 0.0227 | 0.0088 | 0.00972389 |
| 105 | rs223942 | G | -0.00873474 | 0.00124814 | 1.30E-12 | -0.0083 | 0.0086 | 0.3357 |
| 106 | rs2281175 | C | 0.0113782 | 0.00129339 | 7.40E-19 | -0.0051 | 0.0093 | 0.582801 |
| 107 | rs2286028 | C | 0.0165585 | 0.00151626 | 2.70E-29 | 0.016 | 0.0129 | 0.2168 |
| 108 | rs2320731 | T | 0.00826623 | 0.00130254 | 9.90E-11 | 0.0077 | 0.0094 | 0.4113 |
| 109 | rs2358947 | G | -0.00969299 | 0.00175924 | 4.30E-08 | -0.0069 | 0.0095 | 0.4689 |
| 110 | rs236587 | C | -0.00843594 | 0.001399 | 9.30E-10 | 0.0047 | 0.0087 | 0.592199 |
| 111 | rs238819 | C | 0.00954743 | 0.00159163 | 2.60E-09 | 0.0162 | 0.01 | 0.1052 |
| 112 | rs2442497 | T | -0.0109625 | 0.00178319 | 1.40E-09 | -0.0059 | 0.0114 | 0.6047 |
| 113 | rs251388 | T | -0.0095493 | 0.00121755 | 6.80E-15 | -0.0032 | 0.0092 | 0.728501 |
| 114 | rs2592208 | A | -0.00657976 | 0.0012188 | 3.50E-08 | -0.0016 | 0.0084 | 0.8451 |
| 115 | rs2615074 | A | 0.00966192 | 0.00125162 | 2.80E-15 | 0.0052 | 0.0089 | 0.5592 |
| 116 | rs2627692 | T | -0.00987569 | 0.00121806 | 6.10E-17 | -0.0016 | 0.0086 | 0.8534 |
| 117 | rs2749807 | T | 0.0076709 | 0.00129486 | 2.00E-10 | 0.0024 | 0.0086 | 0.784099 |
| 118 | rs2756895 | T | 0.00759888 | 0.00121955 | 1.20E-09 | 0.0099 | 0.0085 | 0.2433 |
| 119 | rs28445331 | C | 0.0074215 | 0.00125967 | 2.00E-09 | -0.0066 | 0.0086 | 0.444 |
| 120 | rs28575622 | T | 0.0166054 | 0.00253745 | 1.40E-11 | 0.0043 | 0.0178 | 0.8084 |
| 121 | rs29946 | C | 0.00774908 | 0.00124964 | 2.10E-09 | 0.0143 | 0.0101 | 0.1558 |
| 122 | rs3013301 | A | -0.00685818 | 0.00127614 | 4.70E-08 | -0.0048 | 0.0093 | 0.6078 |
| 123 | rs3116603 | C | -0.027381 | 0.00148378 | 1.30E-77 | 0.0263 | 0.0149 | 0.0770105 |
| 124 | rs32736 | G | 0.00684865 | 0.0012795 | 4.00E-08 | -0.0021 | 0.0095 | 0.8272 |
| 125 | rs34693680 | T | 0.0128338 | 0.00178742 | 1.40E-13 | 0.0025 | 0.0183 | 0.8919 |
| 126 | rs34825238 | T | 0.00810291 | 0.00141182 | 1.10E-08 | -0.0212 | 0.0111 | 0.0558599 |
| 127 | rs35050648 | T | 0.00907837 | 0.00143679 | 1.90E-10 | -0.0089 | 0.0117 | 0.4478 |
| 128 | rs35446432 | T | 0.00711527 | 0.00129235 | 1.90E-08 | 0.0087 | 0.0092 | 0.3401 |
| 129 | rs35492502 | A | 0.00967173 | 0.00132361 | 2.50E-13 | -0.0056 | 0.0114 | 0.625 |
| 130 | rs359938 | G | -0.0092321 | 0.00143701 | 1.70E-10 | 0.0193 | 0.0136 | 0.1559 |
| 131 | rs36073309 | A | 0.00832163 | 0.00129455 | 1.50E-10 | 0.0052 | 0.0099 | 0.6016 |
| 132 | rs36089326 | T | 0.00736535 | 0.00129415 | 7.80E-09 | 0.006 | 0.012 | 0.615399 |
| 133 | rs3772051 | A | -0.00819723 | 0.00144342 | 8.80E-09 | -4.00E-04 | 0.0126 | 0.9759 |
| 134 | rs3778157 | C | 0.0110266 | 0.00162305 | 4.20E-12 | 0.0335 | 0.0156 | 0.0315 |
| 135 | rs3808424 | C | -0.0156684 | 0.00147209 | 4.10E-26 | -4.00E-04 | 0.0087 | 0.9638 |
| 136 | rs3845344 | T | 0.0091788 | 0.00123617 | 3.50E-14 | 0.0061 | 0.009 | 0.4993 |
| 137 | rs40393 | C | 0.00769746 | 0.00124844 | 1.60E-09 | 0.0059 | 0.0096 | 0.541 |
| 138 | rs4235838 | A | -0.00834798 | 0.00129969 | 2.20E-10 | 0.003 | 0.0087 | 0.7309 |
| 139 | rs4257594 | T | -0.00726035 | 0.00125748 | 2.60E-08 | 2.00E-04 | 0.009 | 0.9848 |
| 140 | rs4291242 | C | 0.00829704 | 0.00145756 | 5.10E-09 | 0.0318 | 0.0097 | 0.000982811 |
| 141 | rs4439140 | A | -0.00972751 | 0.00126402 | 6.10E-15 | 0.0152 | 0.0087 | 0.0793999 |
| 142 | rs4525525 | T | -0.0106369 | 0.00140076 | 1.30E-14 | -0.0031 | 0.0096 | 0.743401 |
| 143 | rs4566595 | A | 0.00727039 | 0.0012622 | 4.30E-09 | 0.0021 | 0.0091 | 0.8156 |
| 144 | rs4648626 | A | 0.00789919 | 0.00121348 | 5.40E-11 | -0.0164 | 0.0086 | 0.0574698 |
| 145 | rs4676442 | C | 0.00746662 | 0.00126062 | 3.90E-10 | 0.0093 | 0.0085 | 0.273 |
| 146 | rs4727295 | A | -0.0170036 | 0.0016162 | 2.20E-26 | 0.014 | 0.0173 | 0.4192 |
| 147 | rs4795318 | T | 0.00863275 | 0.0012214 | 2.60E-12 | -0.0174 | 0.0092 | 0.0566905 |
| 148 | rs4800670 | C | -0.00691881 | 0.0012578 | 3.00E-08 | 0.002 | 0.009 | 0.8211 |
| 149 | rs4912537 | T | -0.0125205 | 0.00142205 | 5.70E-19 | -0.0129 | 0.0104 | 0.2118 |
| 150 | rs4912905 | C | -0.0100783 | 0.00140773 | 7.60E-13 | 0.0053 | 0.0091 | 0.5577 |
| 151 | rs4948293 | A | -0.0105384 | 0.00150107 | 1.10E-12 | 0.0095 | 0.0104 | 0.361 |
| 152 | rs5019542 | T | -0.00894166 | 0.00126069 | 1.20E-13 | -0.0055 | 0.0095 | 0.5639 |
| 153 | rs528378 | C | 0.00705542 | 0.00126092 | 1.40E-08 | 0.0187 | 0.0138 | 0.1728 |
| 154 | rs532499 | C | -0.00863852 | 0.00139547 | 4.70E-10 | 0.0044 | 0.0116 | 0.701501 |
| 155 | rs55674305 | A | -0.00964482 | 0.00132353 | 1.20E-13 | -0.0071 | 0.0108 | 0.510199 |
| 156 | rs55800172 | A | 0.0189177 | 0.00247115 | 2.20E-14 | -0.0486 | 0.0311 | 0.1178 |
| 157 | rs55854145 | C | -0.0172474 | 0.00269612 | 4.80E-11 | -0.0125 | 0.0322 | 0.6967 |
| 158 | rs56130943 | C | 0.00940205 | 0.00148129 | 3.00E-10 | 0.0078 | 0.0167 | 0.6404 |
| 159 | rs56207600 | A | 0.0136817 | 0.00194733 | 4.70E-12 | 0.0206 | 0.0132 | 0.1172 |
| 160 | rs56304624 | A | -0.00676137 | 0.00124345 | 2.50E-08 | -0.007 | 0.0087 | 0.4207 |
| 161 | rs57135834 | T | -0.0160957 | 0.00141391 | 1.20E-29 | -0.0235 | 0.0104 | 0.0246502 |
| 162 | rs599004 | T | -0.0101078 | 0.00134756 | 2.40E-14 | -0.0165 | 0.0095 | 0.0816394 |
| 163 | rs60071805 | A | 0.00746956 | 0.00130594 | 3.80E-08 | 0.0088 | 0.0107 | 0.4104 |
| 164 | rs6056342 | A | -0.00767753 | 0.00129089 | 7.50E-10 | -0.0101 | 0.0101 | 0.3166 |
| 165 | rs60667771 | C | 0.0104829 | 0.00165495 | 1.80E-10 | 0.0094 | 0.0099 | 0.3405 |
| 166 | rs6142059 | C | 0.00973613 | 0.0012236 | 1.70E-17 | 0.0129 | 0.0085 | 0.1272 |
| 167 | rs61628776 | G | -0.0145523 | 0.00175353 | 3.90E-17 | 0.0057 | 0.011 | 0.6034 |
| 168 | rs61775433 | G | 0.0131892 | 0.00158491 | 2.90E-17 | -0.0162 | 0.0125 | 0.1953 |
| 169 | rs62275882 | A | -0.0107818 | 0.00174749 | 4.30E-10 | 0.0053 | 0.0201 | 0.7935 |
| 170 | rs632224 | G | -0.0120826 | 0.00123139 | 1.30E-22 | -0.013 | 0.0085 | 0.1246 |
| 171 | rs6493780 | G | -0.0134594 | 0.00212751 | 2.30E-10 | -0.0337 | 0.0232 | 0.1474 |
| 172 | rs6563808 | C | -0.00992402 | 0.00138759 | 1.10E-12 | 0.007 | 0.009 | 0.4368 |
| 173 | rs6693481 | C | -0.00787115 | 0.00131386 | 2.70E-09 | -0.0077 | 0.0087 | 0.3762 |
| 174 | rs67362530 | A | -0.0154582 | 0.00190473 | 1.80E-17 | 0.0018 | 0.0172 | 0.919 |
| 175 | rs6745626 | T | 0.0090194 | 0.00122922 | 1.90E-14 | -4.00E-04 | 0.0088 | 0.9617 |
| 176 | rs6772164 | A | 0.00897332 | 0.00126491 | 1.60E-12 | -0.0044 | 0.0145 | 0.7604 |
| 177 | rs6779752 | A | -0.0131839 | 0.0012699 | 1.10E-25 | 0.0087 | 0.0106 | 0.4105 |
| 178 | rs6905095 | A | -0.00816381 | 0.00122372 | 7.70E-12 | -0.0063 | 0.0084 | 0.453799 |
| 179 | rs695922 | G | -0.00977492 | 0.00165769 | 1.20E-09 | 0.0185 | 0.0108 | 0.0853493 |
| 180 | rs6975015 | A | 0.0168627 | 0.00189534 | 2.70E-19 | -0.0305 | 0.0217 | 0.1603 |
| 181 | rs71423263 | G | 0.0131324 | 0.0017342 | 6.60E-15 | 0.007 | 0.0131 | 0.594101 |
| 182 | rs7188009 | A | 0.00841701 | 0.0012411 | 1.20E-11 | -0.0269 | 0.0146 | 0.0644006 |
| 183 | rs7245985 | G | -0.011072 | 0.00150737 | 3.00E-13 | -0.0129 | 0.0117 | 0.2722 |
| 184 | rs7269113 | C | -0.00863801 | 0.00155122 | 1.10E-08 | 0.0122 | 0.0174 | 0.4848 |
| 185 | rs72697614 | A | 0.00775289 | 0.00130993 | 9.90E-10 | -0.0027 | 0.0101 | 0.787601 |
| 186 | rs72771080 | T | 0.0138091 | 0.00148901 | 3.50E-21 | -0.0114 | 0.0157 | 0.4688 |
| 187 | rs72820209 | T | 0.00702928 | 0.00123674 | 7.40E-09 | -0.0118 | 0.0135 | 0.3837 |
| 188 | rs72845395 | C | -0.0222242 | 0.00307003 | 2.20E-13 | 0.0498 | 0.0349 | 0.1538 |
| 189 | rs7301341 | C | -0.00948523 | 0.00129534 | 1.20E-13 | 0.0038 | 0.0087 | 0.66 |
| 190 | rs73081811 | G | 0.00878213 | 0.00121732 | 2.60E-13 | -0.0105 | 0.0109 | 0.332 |
| 191 | rs73093103 | T | 0.0241633 | 0.00335482 | 1.30E-13 | -0.0024 | 0.038 | 0.9498 |
| 192 | rs7321045 | A | 0.00945976 | 0.00123758 | 2.50E-15 | -0.0023 | 0.0084 | 0.7813 |
| 193 | rs73791415 | T | -0.00718243 | 0.00126585 | 2.40E-08 | -0.0114 | 0.0107 | 0.2856 |
| 194 | rs7399309 | T | 0.0120441 | 0.00184542 | 3.50E-11 | 0.0149 | 0.0101 | 0.1405 |
| 195 | rs74032128 | G | 0.0216905 | 0.00343987 | 3.40E-10 | -0.056 | 0.0434 | 0.1968 |
| 196 | rs741409 | T | 0.0108485 | 0.00166966 | 9.60E-11 | -0.0017 | 0.012 | 0.8898 |
| 197 | rs7426945 | G | 0.00750398 | 0.00121691 | 2.40E-09 | 0.0058 | 0.0084 | 0.4943 |
| 198 | rs74737644 | G | -0.01409 | 0.00212978 | 7.70E-12 | -0.0113 | 0.0211 | 0.590999 |
| 199 | rs7487292 | G | 0.0102479 | 0.00122966 | 2.60E-17 | -0.0094 | 0.0085 | 0.2651 |
| 200 | rs751894 | T | 0.00917897 | 0.00151504 | 2.00E-10 | 0.0121 | 0.0112 | 0.2789 |
| 201 | rs752070 | G | 0.0108737 | 0.0018214 | 5.80E-10 | -0.0038 | 0.0114 | 0.740099 |
| 202 | rs7546249 | A | 0.0151577 | 0.00135446 | 8.60E-30 | 0.0072 | 0.0092 | 0.4361 |
| 203 | rs7576796 | A | -0.00803553 | 0.00144742 | 4.20E-08 | -0.0101 | 0.016 | 0.5271 |
| 204 | rs76067562 | T | 0.0139888 | 0.00207169 | 1.30E-11 | 0.0271 | 0.0247 | 0.2725 |
| 205 | rs7671407 | A | -0.00755734 | 0.00135575 | 2.20E-08 | -0.0018 | 0.01 | 0.8603 |
| 206 | rs76733024 | G | -0.01454 | 0.00245091 | 9.10E-10 | -0.008 | 0.0234 | 0.732399 |
| 207 | rs7673764 | A | -0.0139224 | 0.00248308 | 1.30E-08 | 0.0258 | 0.0209 | 0.2181 |
| 208 | rs7691068 | C | 0.00663792 | 0.00121378 | 4.60E-08 | 0.0049 | 0.0092 | 0.5926 |
| 209 | rs76951439 | T | 0.0204589 | 0.00271149 | 3.20E-14 | -0.023 | 0.0373 | 0.5386 |
| 210 | rs77093479 | G | -0.0111724 | 0.00165359 | 2.10E-11 | -7.00E-04 | 0.0099 | 0.9423 |
| 211 | rs7719688 | G | -0.00878572 | 0.00123064 | 2.80E-13 | -8.00E-04 | 0.0095 | 0.9317 |
| 212 | rs7755185 | G | 0.00879721 | 0.0013112 | 1.20E-11 | 0.0123 | 0.0086 | 0.1546 |
| 213 | rs7758804 | T | 0.00741954 | 0.0012456 | 1.90E-08 | -0.005 | 0.0097 | 0.6076 |
| 214 | rs7776917 | A | 0.0127745 | 0.00121941 | 4.60E-26 | 1.00E-04 | 0.0087 | 0.9944 |
| 215 | rs781648 | T | -0.0139006 | 0.0024113 | 9.70E-09 | 0.0407 | 0.0282 | 0.1488 |
| 216 | rs7843128 | C | -0.00777863 | 0.00127149 | 5.00E-09 | -0.0091 | 0.0089 | 0.3032 |
| 217 | rs7900548 | G | -0.0138869 | 0.00160846 | 1.40E-17 | 0.033 | 0.0118 | 0.00518203 |
| 218 | rs8017780 | A | -0.00926013 | 0.00149837 | 3.40E-10 | -0.0132 | 0.0117 | 0.2564 |
| 219 | rs80295797 | T | -0.0120873 | 0.00128938 | 2.20E-21 | 0.0091 | 0.0096 | 0.3433 |
| 220 | rs8091374 | A | -0.0101354 | 0.00169263 | 1.30E-09 | -0.0095 | 0.0108 | 0.3774 |
| 221 | rs836532 | A | 0.0110783 | 0.00151656 | 4.80E-14 | 0.0475 | 0.0245 | 0.0525896 |
| 222 | rs878347 | C | -0.00912659 | 0.00126373 | 4.20E-13 | -0.0027 | 0.0086 | 0.756201 |
| 223 | rs882378 | C | 0.00992979 | 0.00132012 | 1.00E-14 | -0.0065 | 0.0213 | 0.759899 |
| 224 | rs903162 | A | 0.00752678 | 0.00131174 | 8.40E-09 | 0.0035 | 0.0093 | 0.7059 |
| 225 | rs9291926 | G | -0.0118167 | 0.0012162 | 2.50E-22 | 0.0081 | 0.0085 | 0.3409 |
| 226 | rs9399656 | T | -0.00838239 | 0.0012173 | 7.40E-12 | -0.0061 | 0.0102 | 0.5481 |
| 227 | rs946197 | C | 0.0137795 | 0.00143529 | 3.00E-21 | 0.0068 | 0.0091 | 0.4553 |
| 228 | rs9480933 | G | -0.012177 | 0.00127221 | 1.40E-22 | -0.0081 | 0.0085 | 0.3387 |
| 229 | rs987204 | A | 0.0103734 | 0.00122123 | 1.70E-17 | -0.0094 | 0.0085 | 0.2684 |
| 230 | rs9951619 | G | 0.0118305 | 0.0014549 | 2.40E-17 | 0.0053 | 0.011 | 0.629901 |
| 231 | rs9967287 | T | 0.00774088 | 0.00140914 | 3.00E-08 | 0.0246 | 0.0088 | 0.00526405 |
| 232 | rs9985795 | C | -0.0071301 | 0.00121915 | 4.10E-09 | -0.0146 | 0.0136 | 0.2836 |
| 233 | rs1795323 | G | 0.00636662 | 0.00121506 | 4.00E-08 | 0.0045 | 0.009 | 0.6131 |
| 234 | rs926436 | A | 0.00916139 | 0.00173375 | 4.20E-08 | 0.0126 | 0.0106 | 0.2339 |
| IS=Ischemic Stroke; EA = effect allele; β = per allele effect on the outcome; SE = standard error;  P value = p-value for the genetic association. | | | | | | | | |
