# Supplementary material for: The association between basal metabolic rate and ischemic stroke: a Mendelian randomization study
Source: Front Neurol. 2025 Mar 3;16:1434740. doi: 10.3389/fneur.2025.1434740 (PMC11912940; doi:10.3389/fneur.2025.1434740)
Supplement: Supplementary file 6 [file Table_3.DOCX]

| **Supplementary Table 3 Detailed information of SNPs used in BMR and IS MR analyses** | | | | | | | | | | | | | | | |
| --- | --- | --- | --- | --- | --- | --- | --- | --- | --- | --- | --- | --- | --- | --- | --- |
| N | SNP | EA | OA | F | beta.exp | se.exp | pval.exp | samplesize.exp | beta.out | se.out | pval.out | samplesize.out | palindromic+ambiguours | non-  confounders | non-  outliers |
| 1 | rs1008158 | G | A | 19.2315 | 0.00894516 | 0.00129017 | 9.80E-13 | 534045 | -0.0041 | 0.0086 | 0.6305 | 484121 | TRUE | TRUE | TRUE |
| 2 | rs10172196 | A | G | 30.6801 | 0.0116365 | 0.00131393 | 2.10E-18 | 534045 | 0.0027 | 0.011 | 0.807 | 484121 | TRUE | TRUE | TRUE |
| 3 | rs10202845 | G | A | 29.3244 | -0.0165309 | 0.00192345 | 9.30E-17 | 534045 | 0.0017 | 0.012 | 0.885 | 484121 | TRUE | TRUE | TRUE |
| 4 | rs1024889 | G | A | 14.7613 | 0.00789197 | 0.00128678 | 1.30E-09 | 534045 | -0.0129 | 0.0136 | 0.3437 | 484121 | TRUE | TRUE | TRUE |
| 5 | rs1037702 | A | G | 16.4620 | -0.00809593 | 0.00125695 | 2.40E-10 | 534045 | 0.0096 | 0.009 | 0.2838 | 484121 | TRUE | TRUE | TRUE |
| 6 | rs1045475 | G | A | 62.3554 | 0.0199626 | 0.00159022 | 1.40E-35 | 534045 | 0.0045 | 0.0097 | 0.6417 | 484121 | TRUE | TRUE | TRUE |
| 7 | rs10457469 | A | G | 53.7266 | 0.0142001 | 0.00121469 | 3.60E-32 | 534045 | -0.002 | 0.0089 | 0.8245 | 484121 | TRUE | TRUE | TRUE |
| 8 | rs1048932 | A | C | 13.1346 | -0.00712123 | 0.00123581 | 3.70E-08 | 534045 | 1.00E-04 | 0.0085 | 0.9867 | 484121 | TRUE | TRUE | TRUE |
| 9 | rs1064213 | A | G | 26.5002 | 0.00997141 | 0.00120794 | 3.90E-17 | 534045 | -0.0014 | 0.009 | 0.8779 | 484121 | TRUE | TRUE | TRUE |
| 10 | rs10735418 | C | T | 26.2594 | -0.0102485 | 0.00125364 | 2.50E-16 | 534045 | 4.00E-04 | 0.0086 | 0.9621 | 484121 | TRUE | TRUE | TRUE |
| 11 | rs10738451 | C | T | 12.6382 | 0.00764488 | 0.00135699 | 6.90E-09 | 534045 | 0.0165 | 0.011 | 0.1343 | 484121 | TRUE | TRUE | TRUE |
| 12 | rs10777860 | A | G | 23.2803 | -0.00935217 | 0.00121578 | 2.00E-15 | 534045 | -0.0061 | 0.0087 | 0.4841 | 484121 | TRUE | TRUE | TRUE |
| 13 | rs10838703 | G | C | 19.2720 | -0.00918571 | 0.00131883 | 3.30E-13 | 534045 | -0.0253 | 0.0102 | 0.0133601 | 484121 | TRUE | TRUE | TRUE |
| 14 | rs10870597 | G | A | 21.1137 | -0.0104855 | 0.0014396 | 7.50E-13 | 534045 | 0.0232 | 0.0099 | 0.01907 | 484121 | TRUE | TRUE | TRUE |
| 15 | rs10878984 | T | C | 32.4129 | 0.01159 | 0.00128235 | 2.70E-20 | 534045 | 0.0015 | 0.0089 | 0.866 | 484121 | TRUE | TRUE | TRUE |
| 16 | rs10925635 | C | A | 18.2021 | 0.0086161 | 0.00125653 | 1.20E-11 | 534045 | -0.0136 | 0.0143 | 0.3417 | 484121 | TRUE | TRUE | TRUE |
| 17 | rs10938397 | G | A | 55.3411 | 0.0145209 | 0.00122781 | 2.40E-33 | 534045 | 0.0113 | 0.0089 | 0.204 | 484121 | TRUE | TRUE | TRUE |
| 18 | rs10991925 | C | T | 30.2986 | -0.0118685 | 0.00135934 | 1.40E-17 | 534045 | -0.0022 | 0.0096 | 0.8151 | 484121 | TRUE | TRUE | TRUE |
| 19 | rs11013045 | G | T | 22.1794 | -0.00933398 | 0.00124887 | 1.50E-13 | 534045 | 0.0062 | 0.014 | 0.6595 | 484121 | TRUE | TRUE | TRUE |
| 20 | rs11014285 | A | G | 47.8194 | 0.0180079 | 0.00165846 | 4.40E-27 | 534045 | 0.0153 | 0.019 | 0.4205 | 484121 | TRUE | TRUE | TRUE |
| 21 | rs11031105 | G | C | 33.1814 | 0.0119074 | 0.00130289 | 6.40E-21 | 534045 | 0.0143 | 0.0143 | 0.3161 | 484121 | TRUE | TRUE | TRUE |
| 22 | rs11065015 | T | C | 22.1304 | -0.0279555 | 0.00380318 | 6.80E-13 | 534045 | 0.0366 | 0.0344 | 0.2876 | 484121 | TRUE | TRUE | TRUE |
| 23 | rs11160601 | T | C | 25.8191 | 0.0169837 | 0.00212368 | 3.90E-16 | 534045 | -0.0052 | 0.0101 | 0.6028 | 484121 | TRUE | TRUE | TRUE |
| 24 | rs111640872 | C | G | 63.0535 | 0.0163291 | 0.00130129 | 2.10E-36 | 534045 | -0.0055 | 0.0114 | 0.6296 | 484121 | TRUE | TRUE | TRUE |
| 25 | rs11196169 | G | A | 25.8723 | -0.00985771 | 0.00124435 | 8.50E-15 | 534045 | -0.0112 | 0.0093 | 0.2287 | 484121 | TRUE | TRUE | TRUE |
| 26 | rs11205354 | A | C | 14.1292 | -0.00732021 | 0.00122175 | 1.70E-09 | 534045 | 0.0041 | 0.0087 | 0.636099 | 484121 | TRUE | TRUE | TRUE |
| 27 | rs112069922 | T | C | 30.9622 | -0.0250999 | 0.00286334 | 3.20E-18 | 534045 | 0.0401 | 0.0351 | 0.2533 | 484121 | TRUE | TRUE | TRUE |
| 28 | rs11240565 | T | C | 35.7512 | 0.0118154 | 0.00123165 | 6.50E-22 | 534045 | 0.0041 | 0.0087 | 0.6337 | 484121 | TRUE | TRUE | TRUE |
| 29 | rs11243202 | C | T | 73.8675 | 0.0166377 | 0.00121835 | 5.10E-42 | 534045 | 0.0034 | 0.0094 | 0.7181 | 484121 | TRUE | TRUE | TRUE |
| 30 | rs11245450 | A | G | 26.2431 | -0.0100356 | 0.00124379 | 2.80E-16 | 534045 | 0.005 | 0.0087 | 0.566601 | 484121 | TRUE | TRUE | TRUE |
| 31 | rs112957890 | G | A | 18.7506 | 0.00949271 | 0.00139628 | 8.00E-12 | 534045 | -0.015 | 0.0158 | 0.3443 | 484121 | TRUE | TRUE | TRUE |
| 32 | rs114056237 | A | G | 45.7609 | -0.0591129 | 0.00547287 | 3.80E-27 | 534045 | 0.0884 | 0.0769 | 0.2503 | 484121 | TRUE | TRUE | TRUE |
| 33 | rs114949263 | C | T | 15.2259 | -0.011978 | 0.00193388 | 2.90E-09 | 534045 | -0.0189 | 0.0232 | 0.4165 | 484121 | TRUE | TRUE | TRUE |
| 34 | rs115179432 | G | A | 33.7736 | -0.0217638 | 0.00234816 | 3.40E-20 | 534045 | -0.03 | 0.0274 | 0.273 | 484121 | TRUE | TRUE | TRUE |
| 35 | rs11545482 | T | C | 19.2615 | -0.0299435 | 0.00425009 | 3.10E-12 | 534045 | 0.0407 | 0.0605 | 0.501 | 484121 | TRUE | TRUE | TRUE |
| 36 | rs11681299 | T | C | 21.0408 | 0.00981375 | 0.00133272 | 1.50E-13 | 534045 | -0.0028 | 0.0087 | 0.7443 | 484121 | TRUE | TRUE | TRUE |
| 37 | rs11689727 | A | C | 28.3024 | -0.0109335 | 0.00128408 | 2.50E-17 | 534045 | -0.0116 | 0.011 | 0.2893 | 484121 | TRUE | TRUE | TRUE |
| 38 | rs11709402 | G | A | 32.3743 | 0.012277 | 0.00135595 | 2.60E-20 | 534045 | 0.0163 | 0.0107 | 0.1277 | 484121 | TRUE | TRUE | TRUE |
| 39 | rs11779459 | T | C | 15.3169 | 0.00791602 | 0.00127776 | 3.20E-10 | 534045 | -0.0022 | 0.009 | 0.805 | 484121 | TRUE | TRUE | TRUE |
| 40 | rs11785562 | A | G | 23.6970 | 0.0117589 | 0.00153407 | 6.30E-14 | 534045 | -0.007 | 0.0106 | 0.508401 | 484121 | TRUE | TRUE | TRUE |
| 41 | rs11794152 | G | A | 33.2839 | 0.0113276 | 0.00123766 | 2.80E-20 | 534045 | 0.0093 | 0.0084 | 0.2675 | 484121 | TRUE | TRUE | TRUE |
| 42 | rs117999064 | G | A | 12.6115 | -0.0272276 | 0.00492186 | 3.50E-08 | 534045 | -0.0347 | 0.0742 | 0.6404 | 484121 | TRUE | TRUE | TRUE |
| 43 | rs118173451 | C | T | 14.8335 | -0.0295441 | 0.00492861 | 2.60E-09 | 534045 | -0.0712 | 0.0554 | 0.1988 | 484121 | TRUE | TRUE | TRUE |
| 44 | rs11880992 | A | G | 40.7716 | 0.0125685 | 0.00124219 | 5.70E-25 | 534045 | 0.0073 | 0.0085 | 0.3887 | 484121 | TRUE | TRUE | TRUE |
| 45 | rs11982736 | A | G | 16.2346 | -0.0108169 | 0.00168453 | 1.90E-10 | 534045 | -0.0014 | 0.0091 | 0.876 | 484121 | TRUE | TRUE | TRUE |
| 46 | rs12072845 | A | G | 51.9007 | -0.0142632 | 0.00123671 | 3.50E-31 | 534045 | 0.0041 | 0.0085 | 0.633299 | 484121 | TRUE | TRUE | TRUE |
| 47 | rs12099669 | A | G | 58.3384 | 0.0160691 | 0.00131981 | 9.80E-35 | 534045 | -0.0047 | 0.0107 | 0.6595 | 484121 | TRUE | TRUE | TRUE |
| 48 | rs12129705 | T | A | 16.6211 | -0.0118338 | 0.0018428 | 1.80E-10 | 534045 | 0.0225 | 0.0208 | 0.2801 | 484121 | TRUE | TRUE | TRUE |
| 49 | rs1218822 | A | G | 26.3740 | 0.0105017 | 0.00129183 | 1.10E-16 | 534045 | 0.0032 | 0.0086 | 0.7085 | 484121 | TRUE | TRUE | TRUE |
| 50 | rs12209223 | A | C | 21.4761 | 0.014851 | 0.00201717 | 2.40E-13 | 534045 | 0.0155 | 0.0126 | 0.2193 | 484121 | TRUE | TRUE | TRUE |
| 51 | rs12254441 | T | C | 14.2322 | -0.00754648 | 0.00130304 | 3.40E-09 | 534045 | -0.0095 | 0.015 | 0.5293 | 484121 | TRUE | TRUE | TRUE |
| 52 | rs12314162 | T | C | 64.1041 | 0.0210965 | 0.00165371 | 4.70E-38 | 534045 | -0.0027 | 0.0116 | 0.8175 | 484121 | TRUE | TRUE | TRUE |
| 53 | rs12375196 | A | C | 32.4961 | 0.0111606 | 0.0012368 | 2.10E-19 | 534045 | 0.0018 | 0.0086 | 0.8366 | 484121 | TRUE | TRUE | TRUE |
| 54 | rs12427047 | T | C | 23.8589 | -0.0110184 | 0.00141471 | 3.90E-14 | 534045 | 0.0031 | 0.0096 | 0.7431 | 484121 | TRUE | TRUE | TRUE |
| 55 | rs12443906 | T | C | 41.0225 | -0.0138552 | 0.00136413 | 1.50E-24 | 534045 | -4.00E-04 | 0.0096 | 0.9708 | 484121 | TRUE | TRUE | TRUE |
| 56 | rs12467963 | T | A | 17.3849 | -0.00822069 | 0.00124026 | 5.10E-12 | 534045 | 0.0038 | 0.0088 | 0.664599 | 484121 | TRUE | TRUE | TRUE |
| 57 | rs1250569 | C | T | 12.3961 | 0.00685948 | 0.00123178 | 4.00E-09 | 534045 | -0.0107 | 0.0085 | 0.2072 | 484121 | TRUE | TRUE | TRUE |
| 58 | rs12713004 | G | A | 56.3672 | 0.016275 | 0.00135354 | 6.10E-34 | 534045 | -8.00E-04 | 0.0132 | 0.9519 | 484121 | TRUE | TRUE | TRUE |
| 59 | rs12731187 | T | C | 15.9502 | -0.0080601 | 0.00127228 | 1.40E-10 | 534045 | -0.012 | 0.0085 | 0.1584 | 484121 | TRUE | TRUE | TRUE |
| 60 | rs12738523 | T | A | 14.3840 | -0.00791823 | 0.00130842 | 4.20E-10 | 534045 | -0.0069 | 0.0087 | 0.4278 | 484121 | TRUE | TRUE | TRUE |
| 61 | rs12907384 | C | T | 30.1140 | -0.0106416 | 0.00122401 | 4.50E-19 | 534045 | 0.0244 | 0.0197 | 0.2161 | 484121 | TRUE | TRUE | TRUE |
| 62 | rs1296328 | C | A | 25.1612 | -0.0097756 | 0.00122947 | 8.70E-16 | 534045 | -0.0038 | 0.012 | 0.753199 | 484121 | TRUE | TRUE | TRUE |
| 63 | rs13185520 | A | G | 14.8190 | -0.00753269 | 0.00122435 | 2.90E-10 | 534045 | -0.0168 | 0.0098 | 0.0871004 | 484121 | TRUE | TRUE | TRUE |
| 64 | rs13271368 | T | C | 43.0107 | -0.0149071 | 0.00144944 | 3.10E-25 | 534045 | -0.0259 | 0.011 | 0.0187802 | 484121 | TRUE | TRUE | TRUE |
| 65 | rs13430869 | T | G | 32.8809 | 0.0126932 | 0.00138601 | 7.70E-21 | 534045 | 4.00E-04 | 0.0093 | 0.9685 | 484121 | TRUE | TRUE | TRUE |
| 66 | rs1360371 | C | T | 45.6357 | 0.0156412 | 0.00146227 | 2.90E-27 | 534045 | -0.0042 | 0.0145 | 0.772999 | 484121 | TRUE | TRUE | TRUE |
| 67 | rs141729694 | T | C | 36.7820 | 0.0223909 | 0.00232894 | 2.90E-22 | 534045 | -0.0025 | 0.0168 | 0.8832 | 484121 | TRUE | TRUE | TRUE |
| 68 | rs141866277 | T | A | 19.7574 | -0.0280215 | 0.00400396 | 4.60E-13 | 534045 | 0.0217 | 0.059 | 0.712801 | 484121 | TRUE | TRUE | TRUE |
| 69 | rs143384 | G | A | 290.5616 | 0.0336053 | 0.00124467 | 5.00E-163 | 534045 | -0.0067 | 0.0093 | 0.4714 | 484121 | TRUE | TRUE | TRUE |
| 70 | rs1439287 | A | G | 21.9829 | 0.00907821 | 0.00121548 | 6.60E-14 | 534045 | -6.00E-04 | 0.0085 | 0.9475 | 484121 | TRUE | TRUE | TRUE |
| 71 | rs1443536 | G | A | 34.1417 | 0.0122796 | 0.00132168 | 1.50E-20 | 534045 | -0.0084 | 0.0095 | 0.3782 | 484121 | TRUE | TRUE | TRUE |
| 72 | rs1452822 | A | T | 59.2273 | 0.0164954 | 0.00134809 | 2.80E-35 | 534045 | 0.0082 | 0.01 | 0.4134 | 484121 | TRUE | TRUE | TRUE |
| 73 | rs147110934 | T | G | 20.5084 | -0.028459 | 0.00396106 | 1.70E-13 | 534045 | -0.0613 | 0.0569 | 0.2812 | 484121 | TRUE | TRUE | TRUE |
| 74 | rs1472852 | A | C | 116.9274 | -0.0286675 | 0.00166483 | 1.50E-68 | 534045 | 0.0108 | 0.015 | 0.4736 | 484121 | TRUE | TRUE | TRUE |
| 75 | rs148636479 | T | A | 14.3844 | -0.0220269 | 0.00388552 | 7.50E-09 | 534045 | -0.037 | 0.0544 | 0.4959 | 484121 | TRUE | TRUE | TRUE |
| 76 | rs150059308 | A | G | 13.2926 | 0.0327443 | 0.00563179 | 8.10E-09 | 534045 | -0.042 | 0.0608 | 0.489199 | 484121 | TRUE | TRUE | TRUE |
| 77 | rs1521624 | A | C | 23.8984 | -0.00947681 | 0.00122927 | 4.00E-15 | 534045 | 0.0028 | 0.0086 | 0.7395 | 484121 | TRUE | TRUE | TRUE |
| 78 | rs1573891 | C | G | 57.7529 | -0.0201561 | 0.00168407 | 2.80E-33 | 534045 | -0.028 | 0.0156 | 0.0737004 | 484121 | TRUE | TRUE | TRUE |
| 79 | rs1582931 | A | G | 91.8109 | -0.0185679 | 0.00122457 | 1.20E-52 | 534045 | -0.0063 | 0.0084 | 0.4563 | 484121 | TRUE | TRUE | TRUE |
| 80 | rs1599473 | T | G | 26.1052 | -0.011516 | 0.0014211 | 4.90E-16 | 534045 | -0.0027 | 0.0148 | 0.8542 | 484121 | TRUE | TRUE | TRUE |
| 81 | rs16942324 | A | C | 41.0634 | -0.0382892 | 0.00376801 | 1.60E-24 | 534045 | 0.0407 | 0.05 | 0.4152 | 484121 | TRUE | TRUE | TRUE |
| 82 | rs16996637 | T | C | 20.9650 | 0.0131737 | 0.00183517 | 7.70E-13 | 534045 | 0.0183 | 0.018 | 0.31 | 484121 | TRUE | TRUE | TRUE |
| 83 | rs17024393 | C | T | 29.5498 | 0.0330903 | 0.00380835 | 2.40E-18 | 534045 | 0.0269 | 0.034 | 0.4281 | 484121 | TRUE | TRUE | TRUE |
| 84 | rs17049820 | C | T | 15.3621 | -0.0119777 | 0.00191931 | 8.70E-10 | 534045 | 0.005 | 0.0096 | 0.5991 | 484121 | TRUE | TRUE | TRUE |
| 85 | rs17115481 | A | G | 23.1465 | -0.010492 | 0.00136961 | 3.20E-14 | 534045 | -0.0013 | 0.0108 | 0.9041 | 484121 | TRUE | TRUE | TRUE |
| 86 | rs1721382 | A | C | 14.7243 | 0.00778776 | 0.00127444 | 5.30E-11 | 534045 | -6.00E-04 | 0.0091 | 0.9467 | 484121 | TRUE | TRUE | TRUE |
| 87 | rs17246129 | A | G | 24.5764 | 0.0104244 | 0.00131524 | 2.10E-15 | 534045 | -9.00E-04 | 0.0104 | 0.9307 | 484121 | TRUE | TRUE | TRUE |
| 88 | rs17363646 | G | A | 21.1679 | 0.0130022 | 0.00176022 | 4.00E-14 | 534045 | -0.0085 | 0.0146 | 0.5622 | 484121 | TRUE | TRUE | TRUE |
| 89 | rs17668356 | G | C | 25.7759 | -0.0139082 | 0.00171397 | 5.10E-16 | 534045 | -0.0067 | 0.0114 | 0.5538 | 484121 | TRUE | TRUE | TRUE |
| 90 | rs17812715 | T | G | 15.3077 | 0.00770087 | 0.00123754 | 6.10E-10 | 534045 | -0.0059 | 0.0088 | 0.5049 | 484121 | TRUE | TRUE | TRUE |
| 91 | rs1857423 | C | T | 14.3196 | 0.00826194 | 0.00137571 | 5.00E-09 | 534045 | -8.00E-04 | 0.0124 | 0.9464 | 484121 | TRUE | TRUE | TRUE |
| 92 | rs1927635 | C | T | 17.5971 | 0.00848721 | 0.00128755 | 2.60E-11 | 534045 | 0.008 | 0.0086 | 0.3532 | 484121 | TRUE | TRUE | TRUE |
| 93 | rs1941697 | A | G | 15.9992 | 0.0077805 | 0.00122632 | 1.20E-10 | 534045 | 0.0074 | 0.0092 | 0.4222 | 484121 | TRUE | TRUE | TRUE |
| 94 | rs1984119 | C | T | 36.6849 | -0.0134292 | 0.00141205 | 2.60E-21 | 534045 | -0.0083 | 0.0092 | 0.3675 | 484121 | TRUE | TRUE | TRUE |
| 95 | rs2005172 | C | A | 96.8930 | 0.0198374 | 0.00128443 | 4.10E-55 | 534045 | -0.0042 | 0.0086 | 0.624901 | 484121 | TRUE | TRUE | TRUE |
| 96 | rs2101975 | G | A | 62.1526 | -0.0153983 | 0.00123001 | 1.20E-35 | 534045 | -0.014 | 0.0084 | 0.0949992 | 484121 | TRUE | TRUE | TRUE |
| 97 | rs2104449 | T | G | 31.9882 | 0.0121468 | 0.00134262 | 1.40E-18 | 534045 | 0.0157 | 0.0163 | 0.334 | 484121 | TRUE | TRUE | TRUE |
| 98 | rs2119753 | G | A | 14.8015 | -0.00763019 | 0.00124201 | 9.80E-10 | 534045 | -0.0022 | 0.0087 | 0.799 | 484121 | TRUE | TRUE | TRUE |
| 99 | rs2122823 | T | C | 19.4296 | 0.0104601 | 0.00150714 | 5.30E-12 | 534045 | -0.0025 | 0.0091 | 0.780899 | 484121 | TRUE | TRUE | TRUE |
| 100 | rs2192158 | G | A | 14.0196 | -0.00728792 | 0.00122176 | 4.70E-10 | 534045 | -0.002 | 0.0086 | 0.814 | 484121 | TRUE | TRUE | TRUE |
| 101 | rs2230590 | C | T | 54.3205 | 0.0142694 | 0.00121314 | 4.90E-33 | 534045 | 0.0027 | 0.01 | 0.7901 | 484121 | TRUE | TRUE | TRUE |
| 102 | rs224045 | C | T | 16.8526 | -0.00805291 | 0.00123789 | 9.20E-11 | 534045 | 0.0128 | 0.0084 | 0.1305 | 484121 | TRUE | TRUE | TRUE |
| 103 | rs2252720 | T | C | 40.5531 | -0.0131452 | 0.00131249 | 2.70E-24 | 534045 | -0.0026 | 0.0086 | 0.7602 | 484121 | TRUE | TRUE | TRUE |
| 104 | rs2265309 | C | T | 41.7813 | -0.0125155 | 0.0012184 | 1.00E-25 | 534045 | -0.0114 | 0.0085 | 0.1807 | 484121 | TRUE | TRUE | TRUE |
| 105 | rs2270894 | G | C | 58.4928 | -0.018385 | 0.00156247 | 1.80E-32 | 534045 | -0.0152 | 0.0094 | 0.1047 | 484121 | TRUE | TRUE | TRUE |
| 106 | rs2287214 | G | A | 11.4077 | 0.00667716 | 0.00123923 | 3.80E-08 | 534045 | -6.00E-04 | 0.0111 | 0.958 | 484121 | TRUE | TRUE | TRUE |
| 107 | rs2290616 | T | C | 14.1678 | 0.00818542 | 0.00136448 | 4.20E-09 | 534045 | -0.0011 | 0.0089 | 0.9018 | 484121 | TRUE | TRUE | TRUE |
| 108 | rs2296316 | C | T | 27.3241 | -0.0101414 | 0.00123231 | 2.20E-16 | 534045 | -0.0058 | 0.0094 | 0.5358 | 484121 | TRUE | TRUE | TRUE |
| 109 | rs2386887 | C | A | 12.9558 | 0.00750268 | 0.00131034 | 1.50E-08 | 534045 | -0.0023 | 0.01 | 0.8211 | 484121 | TRUE | TRUE | TRUE |
| 110 | rs2390669 | C | A | 15.3453 | 0.0113105 | 0.00181249 | 4.70E-10 | 534045 | 0.0082 | 0.0295 | 0.7812 | 484121 | TRUE | TRUE | TRUE |
| 111 | rs244711 | T | C | 64.9727 | 0.0168031 | 0.0014016 | 8.10E-34 | 534045 | 0.0103 | 0.0101 | 0.304 | 484121 | TRUE | TRUE | TRUE |
| 112 | rs2533879 | A | G | 98.8834 | -0.0209904 | 0.00132595 | 1.40E-55 | 534045 | 0.0079 | 0.0092 | 0.3882 | 484121 | TRUE | TRUE | TRUE |
| 113 | rs2568958 | A | G | 35.8721 | 0.0118451 | 0.0012331 | 1.20E-22 | 534045 | -0.0118 | 0.0115 | 0.3077 | 484121 | TRUE | TRUE | TRUE |
| 114 | rs2569993 | C | T | 14.7094 | 0.0079541 | 0.00130242 | 7.30E-10 | 534045 | -0.0122 | 0.0088 | 0.1647 | 484121 | TRUE | TRUE | TRUE |
| 115 | rs2578246 | A | G | 14.7500 | -0.00944494 | 0.00154768 | 5.60E-10 | 534045 | 0.0091 | 0.0091 | 0.3176 | 484121 | TRUE | TRUE | TRUE |
| 116 | rs2602713 | C | A | 31.1598 | 0.0108816 | 0.00124143 | 2.90E-18 | 534045 | -0.0053 | 0.0092 | 0.5642 | 484121 | TRUE | TRUE | TRUE |
| 117 | rs2611732 | G | A | 15.8310 | 0.00786492 | 0.00123755 | 2.30E-10 | 534045 | 0.0174 | 0.0087 | 0.04469 | 484121 | TRUE | TRUE | TRUE |
| 118 | rs2650965 | G | A | 18.9795 | -0.00896988 | 0.00130495 | 1.60E-11 | 534045 | -0.0135 | 0.009 | 0.1348 | 484121 | TRUE | TRUE | TRUE |
| 119 | rs2658797 | T | C | 11.6983 | -0.00662315 | 0.00121737 | 3.50E-08 | 534045 | -0.0137 | 0.0119 | 0.2484 | 484121 | TRUE | TRUE | TRUE |
| 120 | rs2716212 | G | A | 12.6599 | 0.00707559 | 0.00125686 | 1.80E-08 | 534045 | -0.0153 | 0.0086 | 0.0770194 | 484121 | TRUE | TRUE | TRUE |
| 121 | rs2736429 | G | A | 34.4965 | 0.0117257 | 0.00171608 | 6.00E-12 | 534045 | 0.0137 | 0.0085 | 0.1089 | 484121 | TRUE | TRUE | TRUE |
| 122 | rs2744956 | C | T | 195.2531 | 0.039074 | 0.00175051 | 4.80E-112 | 534045 | 0.0452 | 0.0181 | 0.0122301 | 484121 | TRUE | TRUE | TRUE |
| 123 | rs284662 | C | T | 24.3143 | -0.00982788 | 0.00125596 | 1.60E-14 | 534045 | -0.0085 | 0.0085 | 0.3156 | 484121 | TRUE | TRUE | TRUE |
| 124 | rs28605759 | A | G | 18.3031 | -0.00831268 | 0.00121196 | 7.60E-12 | 534045 | 0.0142 | 0.0084 | 0.0923592 | 484121 | TRUE | TRUE | TRUE |
| 125 | rs2866719 | T | C | 26.0324 | 0.0102313 | 0.0012643 | 1.70E-15 | 534045 | -5.00E-04 | 0.0086 | 0.9526 | 484121 | TRUE | TRUE | TRUE |
| 126 | rs28701981 | C | T | 68.4675 | 0.0167902 | 0.0012816 | 2.60E-40 | 534045 | 0.0052 | 0.0091 | 0.5644 | 484121 | TRUE | TRUE | TRUE |
| 127 | rs2885697 | T | G | 75.2567 | -0.017783 | 0.00127715 | 3.80E-43 | 534045 | -0.0024 | 0.0089 | 0.792399 | 484121 | TRUE | TRUE | TRUE |
| 128 | rs2900208 | A | C | 42.8475 | 0.0132443 | 0.00127132 | 4.60E-26 | 534045 | -0.0243 | 0.0089 | 0.00659007 | 484121 | TRUE | TRUE | TRUE |
| 129 | rs2950446 | C | T | 31.3559 | -0.0140253 | 0.00157888 | 5.40E-19 | 534045 | -0.0069 | 0.0126 | 0.5846 | 484121 | TRUE | TRUE | TRUE |
| 130 | rs2952615 | C | G | 39.5244 | -0.0125171 | 0.00124841 | 2.90E-23 | 534045 | -0.0101 | 0.0102 | 0.3214 | 484121 | TRUE | TRUE | TRUE |
| 131 | rs2970592 | A | G | 17.1781 | 0.0081496 | 0.00122398 | 2.70E-11 | 534045 | -6.00E-04 | 0.0085 | 0.9457 | 484121 | TRUE | TRUE | TRUE |
| 132 | rs310796 | T | G | 24.3339 | 0.0102417 | 0.00130656 | 9.10E-16 | 534045 | -0.0202 | 0.0111 | 0.0697108 | 484121 | TRUE | TRUE | TRUE |
| 133 | rs3110496 | G | A | 13.4674 | 0.00765298 | 0.00131209 | 1.00E-08 | 534045 | -0.009 | 0.0092 | 0.3281 | 484121 | TRUE | TRUE | TRUE |
| 134 | rs3116201 | A | G | 28.9023 | -0.0175197 | 0.00203546 | 6.40E-18 | 534045 | 0.0388 | 0.0258 | 0.1321 | 484121 | TRUE | TRUE | TRUE |
| 135 | rs31210 | A | G | 28.8933 | -0.0119665 | 0.0013976 | 3.20E-17 | 534045 | -0.006 | 0.0088 | 0.5004 | 484121 | TRUE | TRUE | TRUE |
| 136 | rs3212260 | T | A | 35.2577 | 0.0131273 | 0.00139644 | 1.80E-21 | 534045 | 0.0052 | 0.0103 | 0.611999 | 484121 | TRUE | TRUE | TRUE |
| 137 | rs33973388 | T | G | 33.6535 | 0.0113211 | 0.00123154 | 2.60E-20 | 534045 | -0.01 | 0.0141 | 0.4762 | 484121 | TRUE | TRUE | TRUE |
| 138 | rs34517439 | A | C | 166.8876 | 0.0381746 | 0.00186844 | 2.90E-95 | 534045 | 0.0587 | 0.0206 | 0.004462 | 484121 | TRUE | TRUE | TRUE |
| 139 | rs34776209 | T | C | 48.2824 | -0.0155779 | 0.00141034 | 3.00E-28 | 534045 | 0.0098 | 0.0152 | 0.517399 | 484121 | TRUE | TRUE | TRUE |
| 140 | rs34848742 | G | T | 19.2315 | -0.014909 | 0.00149037 | 3.00E-24 | 534045 | 0.0204 | 0.0139 | 0.1435 | 484121 | TRUE | TRUE | TRUE |
| 141 | rs34879158 | C | A | 30.6801 | -0.0198295 | 0.00139069 | 1.40E-47 | 534045 | -0.0122 | 0.0158 | 0.4411 | 484121 | TRUE | TRUE | TRUE |
| 142 | rs35196838 | A | C | 29.3244 | 0.0102063 | 0.0017392 | 1.50E-08 | 534045 | 0.0132 | 0.01 | 0.1842 | 484121 | TRUE | TRUE | TRUE |
| 143 | rs35467921 | T | C | 14.7613 | 0.0220244 | 0.00124202 | 1.90E-72 | 534045 | 0.0056 | 0.0102 | 0.579399 | 484121 | TRUE | TRUE | TRUE |
| 144 | rs35874463 | G | A | 16.4620 | 0.0200603 | 0.00261464 | 1.40E-14 | 534045 | 0.0279 | 0.0315 | 0.3758 | 484121 | TRUE | TRUE | TRUE |
| 145 | rs36000545 | G | A | 62.3554 | -0.0158045 | 0.00126235 | 1.10E-36 | 534045 | -0.0116 | 0.009 | 0.1975 | 484121 | TRUE | TRUE | TRUE |
| 146 | rs365352 | A | G | 53.7266 | -0.0148384 | 0.001408 | 1.20E-27 | 534045 | -1.00E-04 | 0.0122 | 0.9956 | 484121 | TRUE | TRUE | TRUE |
| 147 | rs3730071 | A | C | 13.1346 | -0.0273843 | 0.00354184 | 1.30E-14 | 534045 | 0.0735 | 0.0491 | 0.1343 | 484121 | TRUE | TRUE | TRUE |
| 148 | rs3759094 | T | C | 26.5002 | -0.0116496 | 0.00128512 | 2.50E-19 | 534045 | 0.0057 | 0.0089 | 0.5242 | 484121 | TRUE | TRUE | TRUE |
| 149 | rs3765351 | T | C | 26.2594 | -0.0085465 | 0.00120978 | 1.60E-13 | 534045 | -0.0133 | 0.0101 | 0.1882 | 484121 | TRUE | TRUE | TRUE |
| 150 | rs3778934 | C | A | 12.6382 | -0.00742547 | 0.00128197 | 2.30E-09 | 534045 | 0.0019 | 0.0087 | 0.8302 | 484121 | TRUE | TRUE | TRUE |
| 151 | rs3810291 | A | G | 23.2803 | 0.0180436 | 0.00130276 | 4.40E-44 | 534045 | 0.0012 | 0.0142 | 0.9343 | 484121 | TRUE | TRUE | TRUE |
| 152 | rs3853252 | A | G | 19.2720 | 0.0152378 | 0.00122298 | 4.30E-36 | 534045 | -0.0031 | 0.0086 | 0.7213 | 484121 | TRUE | TRUE | TRUE |
| 153 | rs390192 | G | A | 21.1137 | -0.0100426 | 0.00122104 | 2.50E-17 | 534045 | -0.015 | 0.0089 | 0.0941109 | 484121 | TRUE | TRUE | TRUE |
| 154 | rs3925 | A | G | 32.4129 | -0.0100386 | 0.00141621 | 3.40E-12 | 534045 | 0.013 | 0.0094 | 0.1648 | 484121 | TRUE | TRUE | TRUE |
| 155 | rs394487 | T | C | 18.2021 | 0.0111436 | 0.00135852 | 2.70E-17 | 534045 | 0.0109 | 0.0093 | 0.241 | 484121 | TRUE | TRUE | TRUE |
| 156 | rs3990738 | G | A | 55.3411 | -0.00719323 | 0.00128859 | 1.50E-08 | 534045 | 0.0017 | 0.0208 | 0.9347 | 484121 | TRUE | TRUE | TRUE |
| 157 | rs3998115 | C | G | 30.2986 | 0.00886175 | 0.00125363 | 1.10E-12 | 534045 | -0.0191 | 0.0116 | 0.0997493 | 484121 | TRUE | TRUE | TRUE |
| 158 | rs40071 | C | T | 22.1794 | -0.00884366 | 0.00158391 | 7.40E-09 | 534045 | -0.0193 | 0.009 | 0.0325597 | 484121 | TRUE | TRUE | TRUE |
| 159 | rs4055791 | T | C | 47.8194 | -0.00914122 | 0.00123883 | 1.90E-14 | 534045 | -0.0013 | 0.0096 | 0.8947 | 484121 | TRUE | TRUE | TRUE |
| 160 | rs41271299 | T | C | 33.1814 | 0.0399701 | 0.00274588 | 2.90E-48 | 534045 | 0.0576 | 0.0379 | 0.1291 | 484121 | TRUE | TRUE | TRUE |
| 161 | rs41311445 | C | A | 22.1304 | -0.0238101 | 0.0020858 | 8.90E-31 | 534045 | 0.0026 | 0.0212 | 0.9008 | 484121 | TRUE | TRUE | TRUE |
| 162 | rs4132132 | C | T | 25.8191 | 0.00939439 | 0.00123553 | 7.40E-15 | 534045 | -0.0117 | 0.0097 | 0.228 | 484121 | TRUE | TRUE | TRUE |
| 163 | rs4240326 | G | A | 63.0535 | -0.0203766 | 0.00122084 | 5.20E-62 | 534045 | -0.0142 | 0.0089 | 0.1098 | 484121 | TRUE | TRUE | TRUE |
| 164 | rs4282339 | A | G | 25.8723 | -0.0171265 | 0.00149207 | 3.70E-31 | 534045 | -0.0014 | 0.0104 | 0.8909 | 484121 | TRUE | TRUE | TRUE |
| 165 | rs4325427 | T | C | 14.1292 | -0.010186 | 0.00134783 | 1.10E-13 | 534045 | 0.0038 | 0.0092 | 0.68 | 484121 | TRUE | TRUE | TRUE |
| 166 | rs4369779 | C | T | 30.9622 | 0.0248722 | 0.00149407 | 4.60E-61 | 534045 | 0.0093 | 0.0103 | 0.3667 | 484121 | TRUE | TRUE | TRUE |
| 167 | rs4489042 | C | G | 35.7512 | -0.00684954 | 0.00125572 | 2.90E-08 | 534045 | -0.0061 | 0.0138 | 0.6598 | 484121 | TRUE | TRUE | TRUE |
| 168 | rs45528934 | T | C | 73.8675 | 0.0173734 | 0.00165401 | 5.10E-26 | 534045 | -0.0039 | 0.0133 | 0.768999 | 484121 | TRUE | TRUE | TRUE |
| 169 | rs4675617 | G | A | 26.2431 | 0.0106743 | 0.00162788 | 3.00E-11 | 534045 | -0.0078 | 0.0189 | 0.6821 | 484121 | TRUE | TRUE | TRUE |
| 170 | rs4680 | A | G | 18.7506 | 0.00802008 | 0.00122651 | 4.20E-11 | 534045 | 0.0202 | 0.0087 | 0.0205902 | 484121 | TRUE | TRUE | TRUE |
| 171 | rs4821940 | C | T | 45.7609 | -0.00840943 | 0.00123369 | 1.90E-11 | 534045 | 0.0058 | 0.0084 | 0.4894 | 484121 | TRUE | TRUE | TRUE |
| 172 | rs4865956 | A | T | 15.2259 | -0.0105075 | 0.00132291 | 2.10E-15 | 534045 | 0.0197 | 0.0092 | 0.0310499 | 484121 | TRUE | TRUE | TRUE |
| 173 | rs4900715 | A | G | 33.7736 | -0.00874865 | 0.00122192 | 1.40E-13 | 534045 | -0.0056 | 0.009 | 0.532 | 484121 | TRUE | TRUE | TRUE |
| 174 | rs4909912 | T | C | 19.2615 | -0.0172714 | 0.00123215 | 2.10E-45 | 534045 | -0.0066 | 0.0084 | 0.4314 | 484121 | TRUE | TRUE | TRUE |
| 175 | rs4980826 | A | C | 21.0408 | 0.00832533 | 0.00124007 | 3.60E-11 | 534045 | 0.001 | 0.0087 | 0.9096 | 484121 | TRUE | TRUE | TRUE |
| 176 | rs520161 | C | T | 28.3024 | -0.0110899 | 0.00132952 | 1.30E-15 | 534045 | -0.0135 | 0.0096 | 0.1632 | 484121 | TRUE | TRUE | TRUE |
| 177 | rs540652 | T | C | 32.3743 | 0.0101282 | 0.00120719 | 3.70E-16 | 534045 | -0.0039 | 0.0084 | 0.640801 | 484121 | TRUE | TRUE | TRUE |
| 178 | rs543874 | G | A | 15.3169 | 0.0282064 | 0.00149409 | 1.70E-81 | 534045 | 0.0068 | 0.0102 | 0.5049 | 484121 | TRUE | TRUE | TRUE |
| 179 | rs55758152 | A | G | 23.6970 | 0.00842842 | 0.00130838 | 1.80E-10 | 534045 | 0.0247 | 0.0145 | 0.0875992 | 484121 | TRUE | TRUE | TRUE |
| 180 | rs56228832 | A | T | 33.2839 | -0.0198991 | 0.00140672 | 1.20E-45 | 534045 | -0.0262 | 0.0146 | 0.0729206 | 484121 | TRUE | TRUE | TRUE |
| 181 | rs56383938 | G | A | 12.6115 | -0.0145575 | 0.00215009 | 1.40E-12 | 534045 | -0.0245 | 0.0233 | 0.2929 | 484121 | TRUE | TRUE | TRUE |
| 182 | rs5752989 | A | G | 14.8335 | -0.0105387 | 0.00123807 | 2.10E-17 | 534045 | 0.0099 | 0.0102 | 0.3354 | 484121 | TRUE | TRUE | TRUE |
| 183 | rs57635800 | A | G | 40.7716 | 0.0130234 | 0.00134757 | 3.20E-23 | 534045 | 0.0075 | 0.0103 | 0.4679 | 484121 | TRUE | TRUE | TRUE |
| 184 | rs57636386 | C | T | 16.2346 | -0.0256001 | 0.00221099 | 2.10E-32 | 534045 | -0.0027 | 0.0131 | 0.8375 | 484121 | TRUE | TRUE | TRUE |
| 185 | rs5771118 | C | T | 51.9007 | 0.0091639 | 0.00140711 | 1.10E-10 | 534045 | -0.0115 | 0.0099 | 0.2456 | 484121 | TRUE | TRUE | TRUE |
| 186 | rs582780 | G | A | 58.3384 | 0.0153369 | 0.00123761 | 1.60E-34 | 534045 | -0.0086 | 0.0118 | 0.4642 | 484121 | TRUE | TRUE | TRUE |
| 187 | rs58584712 | A | G | 16.6211 | 0.00903929 | 0.00147907 | 1.30E-09 | 534045 | -0.0022 | 0.0173 | 0.8969 | 484121 | TRUE | TRUE | TRUE |
| 188 | rs59985551 | T | C | 26.3740 | -0.0179132 | 0.00144166 | 2.90E-35 | 534045 | 0.0107 | 0.0098 | 0.2729 | 484121 | TRUE | TRUE | TRUE |
| 189 | rs60077625 | A | G | 21.4761 | 0.0142083 | 0.00130001 | 1.60E-27 | 534045 | 0.0093 | 0.0098 | 0.3405 | 484121 | TRUE | TRUE | TRUE |
| 190 | rs6026578 | G | C | 14.2322 | -0.0100998 | 0.00126761 | 2.60E-15 | 534045 | 0.0095 | 0.0091 | 0.2961 | 484121 | TRUE | TRUE | TRUE |
| 191 | rs6096886 | G | A | 64.1041 | -0.018949 | 0.00155603 | 2.50E-33 | 534045 | -0.0139 | 0.0111 | 0.2094 | 484121 | TRUE | TRUE | TRUE |
| 192 | rs61729527 | T | C | 32.4961 | -0.0214159 | 0.00274485 | 4.40E-15 | 534045 | -0.0304 | 0.0286 | 0.2888 | 484121 | TRUE | TRUE | TRUE |
| 193 | rs61828917 | T | C | 23.8589 | 0.0108089 | 0.00183321 | 3.90E-09 | 534045 | -0.0179 | 0.0112 | 0.1087 | 484121 | TRUE | TRUE | TRUE |
| 194 | rs61849823 | C | T | 41.0225 | 0.0111739 | 0.00165548 | 2.50E-11 | 534045 | 0.0083 | 0.0193 | 0.668599 | 484121 | TRUE | TRUE | TRUE |
| 195 | rs61855849 | A | G | 17.3849 | -0.0100738 | 0.00139197 | 2.30E-12 | 534045 | -0.0026 | 0.0099 | 0.7922 | 484121 | TRUE | TRUE | TRUE |
| 196 | rs61869763 | T | C | 12.3961 | 0.0173261 | 0.00200517 | 3.30E-18 | 534045 | 0.0087 | 0.012 | 0.4688 | 484121 | TRUE | TRUE | TRUE |
| 197 | rs61992671 | G | A | 56.3672 | -0.011216 | 0.00127529 | 2.60E-18 | 534045 | 0.0044 | 0.013 | 0.733199 | 484121 | TRUE | TRUE | TRUE |
| 198 | rs62246314 | A | G | 15.9502 | 0.0139944 | 0.00199674 | 1.30E-12 | 534045 | -0.0027 | 0.0247 | 0.9135 | 484121 | TRUE | TRUE | TRUE |
| 199 | rs6235 | G | C | 14.3840 | 0.0164596 | 0.00136817 | 4.10E-34 | 534045 | 0.0041 | 0.0098 | 0.6791 | 484121 | TRUE | TRUE | TRUE |
| 200 | rs62372052 | G | A | 30.1140 | 0.0245679 | 0.00194315 | 1.70E-36 | 534045 | 0.0061 | 0.0214 | 0.773701 | 484121 | TRUE | TRUE | TRUE |
| 201 | rs62621197 | T | C | 25.1612 | -0.0338421 | 0.00335251 | 5.60E-24 | 534045 | -0.0017 | 0.0425 | 0.9685 | 484121 | TRUE | TRUE | TRUE |
| 202 | rs6457762 | C | T | 14.8190 | 0.0154594 | 0.0016989 | 1.80E-20 | 534045 | 0.0255 | 0.0113 | 0.0243899 | 484121 | TRUE | TRUE | TRUE |
| 203 | rs655598 | A | G | 43.0107 | -0.00951773 | 0.00121561 | 4.20E-15 | 534045 | -0.0077 | 0.0086 | 0.3676 | 484121 | TRUE | TRUE | TRUE |
| 204 | rs6570509 | T | G | 32.8809 | -0.0141029 | 0.00134009 | 2.60E-26 | 534045 | -0.0038 | 0.0088 | 0.6627 | 484121 | TRUE | TRUE | TRUE |
| 205 | rs667515 | C | G | 45.6357 | -0.0113291 | 0.00125391 | 5.20E-19 | 534045 | 0.015 | 0.0106 | 0.1568 | 484121 | TRUE | TRUE | TRUE |
| 206 | rs6675689 | C | A | 36.7820 | 0.00762259 | 0.00127021 | 1.00E-09 | 534045 | 0.011 | 0.0093 | 0.2346 | 484121 | TRUE | TRUE | TRUE |
| 207 | rs6738207 | A | G | 19.7574 | 0.00714641 | 0.00123612 | 1.50E-08 | 534045 | 0.0011 | 0.0085 | 0.8939 | 484121 | TRUE | TRUE | TRUE |
| 208 | rs67551338 | T | C | 290.5616 | 0.0235436 | 0.00255474 | 1.10E-20 | 534045 | -0.019 | 0.0312 | 0.542199 | 484121 | TRUE | TRUE | TRUE |
| 209 | rs6762578 | A | G | 21.9829 | 0.0154374 | 0.00146429 | 1.60E-26 | 534045 | -0.002 | 0.0099 | 0.8424 | 484121 | TRUE | TRUE | TRUE |
| 210 | rs6804915 | A | C | 34.1417 | 0.00903647 | 0.00133428 | 3.00E-12 | 534045 | -0.0163 | 0.0097 | 0.0939896 | 484121 | TRUE | TRUE | TRUE |
| 211 | rs6821305 | C | A | 59.2273 | 0.012837 | 0.00124025 | 9.20E-25 | 534045 | 0.0045 | 0.0201 | 0.823 | 484121 | TRUE | TRUE | TRUE |
| 212 | rs6874142 | G | T | 20.5084 | 0.0171976 | 0.00202055 | 1.10E-17 | 534045 | 0.0218 | 0.023 | 0.3427 | 484121 | TRUE | TRUE | TRUE |
| 213 | rs6902789 | A | G | 116.9274 | 0.0110311 | 0.00126247 | 1.50E-18 | 534045 | 0.0029 | 0.0094 | 0.754899 | 484121 | TRUE | TRUE | TRUE |
| 214 | rs6946091 | G | A | 14.3844 | 0.0185658 | 0.00125878 | 9.40E-50 | 534045 | -0.004 | 0.0109 | 0.717599 | 484121 | TRUE | TRUE | TRUE |
| 215 | rs7033487 | C | T | 13.2926 | -0.0209549 | 0.00152885 | 1.10E-42 | 534045 | 0.0282 | 0.0165 | 0.0873494 | 484121 | TRUE | TRUE | TRUE |
| 216 | rs703593 | G | A | 23.8984 | -0.0112393 | 0.00121499 | 2.90E-20 | 534045 | 0.0148 | 0.0084 | 0.0788697 | 484121 | TRUE | TRUE | TRUE |
| 217 | rs7072873 | T | C | 57.7529 | -0.0105678 | 0.00122636 | 2.90E-18 | 534045 | 0.0045 | 0.0084 | 0.5897 | 484121 | TRUE | TRUE | TRUE |
| 218 | rs7115013 | T | C | 91.8109 | -0.00749496 | 0.00122778 | 5.70E-10 | 534045 | -0.0032 | 0.0085 | 0.705399 | 484121 | TRUE | TRUE | TRUE |
| 219 | rs7129320 | A | G | 26.1052 | -0.0214691 | 0.0016349 | 1.80E-39 | 534045 | 0.0169 | 0.012 | 0.1594 | 484121 | TRUE | TRUE | TRUE |
| 220 | rs7134283 | A | G | 41.0634 | -0.0118095 | 0.00135088 | 1.50E-18 | 534045 | -0.031 | 0.0121 | 0.0102601 | 484121 | TRUE | TRUE | TRUE |
| 221 | rs71385734 | G | T | 20.9650 | -0.0227637 | 0.00163183 | 1.30E-47 | 534045 | -0.0155 | 0.018 | 0.3882 | 484121 | TRUE | TRUE | TRUE |
| 222 | rs7154982 | A | G | 29.5498 | -0.0140434 | 0.00137622 | 5.70E-25 | 534045 | 0.0031 | 0.0123 | 0.8005 | 484121 | TRUE | TRUE | TRUE |
| 223 | rs7156335 | C | T | 15.3621 | 0.0172037 | 0.00213349 | 8.10E-16 | 534045 | 0.0196 | 0.0157 | 0.2121 | 484121 | TRUE | TRUE | TRUE |
| 224 | rs7199856 | T | G | 23.1465 | -0.00838283 | 0.00140824 | 7.80E-10 | 534045 | 7.00E-04 | 0.0091 | 0.9418 | 484121 | TRUE | TRUE | TRUE |
| 225 | rs7223535 | A | G | 14.7243 | -0.0247128 | 0.00137133 | 1.60E-73 | 534045 | 0.0223 | 0.0138 | 0.1064 | 484121 | TRUE | TRUE | TRUE |
| 226 | rs7229520 | A | G | 24.5764 | -0.0101299 | 0.00129204 | 1.20E-15 | 534045 | 0.0239 | 0.0086 | 0.005332 | 484121 | TRUE | TRUE | TRUE |
| 227 | rs723149 | G | A | 21.1679 | -0.0110854 | 0.00122586 | 6.70E-19 | 534045 | 0.002 | 0.0086 | 0.815 | 484121 | TRUE | TRUE | TRUE |
| 228 | rs724016 | G | A | 25.7759 | 0.0297052 | 0.00121872 | 8.59E-136 | 534045 | 0.0146 | 0.0086 | 0.0894891 | 484121 | TRUE | TRUE | TRUE |
| 229 | rs726547 | A | G | 15.3077 | -0.0200005 | 0.00292449 | 1.80E-11 | 534045 | -0.031 | 0.0104 | 0.00284302 | 484121 | TRUE | TRUE | TRUE |
| 230 | rs72656010 | C | T | 14.3196 | -0.0347319 | 0.00180277 | 6.20E-82 | 534045 | 0.0152 | 0.0142 | 0.2857 | 484121 | TRUE | TRUE | TRUE |
| 231 | rs72755233 | A | G | 17.5971 | -0.0146092 | 0.00193574 | 1.10E-13 | 534045 | -0.0306 | 0.0214 | 0.1535 | 484121 | TRUE | TRUE | TRUE |
| 232 | rs72801843 | A | T | 15.9992 | 0.0161306 | 0.0013257 | 3.60E-35 | 534045 | 0.0192 | 0.0214 | 0.3699 | 484121 | TRUE | TRUE | TRUE |
| 233 | rs73052033 | C | T | 36.6849 | -0.0168741 | 0.00156269 | 7.30E-27 | 534045 | 0.0102 | 0.0166 | 0.5385 | 484121 | TRUE | TRUE | TRUE |
| 234 | rs73175572 | G | A | 96.8930 | 0.0260563 | 0.00194427 | 1.30E-42 | 534045 | -0.0198 | 0.0214 | 0.3546 | 484121 | TRUE | TRUE | TRUE |
| 235 | rs73213484 | T | A | 62.1526 | -0.0134471 | 0.00174884 | 4.70E-14 | 534045 | 0.0145 | 0.014 | 0.3015 | 484121 | TRUE | TRUE | TRUE |
| 236 | rs73619441 | G | T | 31.9882 | -0.0135104 | 0.00174865 | 4.50E-15 | 534045 | 0.0073 | 0.0098 | 0.4535 | 484121 | TRUE | TRUE | TRUE |
| 237 | rs7377083 | A | C | 14.8015 | 0.00958917 | 0.00123599 | 1.90E-15 | 534045 | -0.0131 | 0.0092 | 0.1556 | 484121 | TRUE | TRUE | TRUE |
| 238 | rs73966422 | G | C | 19.4296 | 0.0150136 | 0.00184089 | 1.70E-16 | 534045 | -0.0259 | 0.0161 | 0.1071 | 484121 | TRUE | TRUE | TRUE |
| 239 | rs74494415 | T | C | 14.0196 | -0.0252977 | 0.00314827 | 4.70E-16 | 534045 | 0.0018 | 0.016 | 0.9099 | 484121 | TRUE | TRUE | TRUE |
| 240 | rs7460093 | A | G | 54.3205 | 0.00972935 | 0.0012297 | 2.80E-15 | 534045 | 0.0176 | 0.0137 | 0.1999 | 484121 | TRUE | TRUE | TRUE |
| 241 | rs7519259 | A | G | 16.8526 | 0.00843428 | 0.00121468 | 4.10E-12 | 534045 | 0.0038 | 0.0099 | 0.7013 | 484121 | TRUE | TRUE | TRUE |
| 242 | rs754635 | G | C | 40.5531 | 0.0130636 | 0.00190931 | 3.30E-12 | 534045 | 0.0045 | 0.0104 | 0.6627 | 484121 | TRUE | TRUE | TRUE |
| 243 | rs76040172 | A | G | 41.7813 | -0.0173302 | 0.00272729 | 8.60E-11 | 534045 | -0.0138 | 0.0144 | 0.3365 | 484121 | TRUE | TRUE | TRUE |
| 244 | rs7619139 | A | T | 58.4928 | 0.0128553 | 0.00123379 | 1.20E-25 | 534045 | 0.0047 | 0.0085 | 0.5824 | 484121 | TRUE | TRUE | TRUE |
| 245 | rs76307059 | G | C | 11.4077 | -0.0195087 | 0.00289943 | 4.90E-11 | 534045 | 0.0452 | 0.0244 | 0.0637206 | 484121 | TRUE | TRUE | TRUE |
| 246 | rs76364830 | A | G | 14.1678 | -0.0222943 | 0.00252013 | 7.00E-19 | 534045 | -0.0084 | 0.0151 | 0.5759 | 484121 | TRUE | TRUE | TRUE |
| 247 | rs76513770 | C | T | 27.3241 | -0.0166978 | 0.00182238 | 1.10E-20 | 534045 | -0.0058 | 0.0129 | 0.649901 | 484121 | TRUE | TRUE | TRUE |
| 248 | rs7665127 | T | C | 12.9558 | 0.00988975 | 0.00129377 | 1.90E-14 | 534045 | -3.00E-04 | 0.0092 | 0.9752 | 484121 | TRUE | TRUE | TRUE |
| 249 | rs76693355 | C | T | 15.3453 | -0.014207 | 0.00190087 | 3.10E-14 | 534045 | 0.0148 | 0.0122 | 0.2242 | 484121 | TRUE | TRUE | TRUE |
| 250 | rs76798800 | T | G | 64.9727 | 0.021599 | 0.00136791 | 1.40E-57 | 534045 | 0.0117 | 0.0137 | 0.3924 | 484121 | TRUE | TRUE | TRUE |
| 251 | rs77165542 | T | C | 98.8834 | -0.0636649 | 0.00330073 | 7.50E-86 | 534045 | 0.0311 | 0.0429 | 0.4688 | 484121 | TRUE | TRUE | TRUE |
| 252 | rs7728095 | G | A | 35.8721 | 0.0129997 | 0.00125549 | 4.40E-26 | 534045 | -0.0049 | 0.01 | 0.625299 | 484121 | TRUE | TRUE | TRUE |
| 253 | rs7728690 | T | C | 14.7094 | -0.00967582 | 0.00124542 | 1.30E-14 | 534045 | -2.00E-04 | 0.0087 | 0.9795 | 484121 | TRUE | TRUE | TRUE |
| 254 | rs7731023 | G | A | 14.7500 | 0.00785695 | 0.0012257 | 1.60E-10 | 534045 | -0.0145 | 0.0109 | 0.1833 | 484121 | TRUE | TRUE | TRUE |
| 255 | rs7740107 | A | T | 31.1598 | -0.0278651 | 0.00137448 | 1.80E-95 | 534045 | -0.0011 | 0.014 | 0.9367 | 484121 | TRUE | TRUE | TRUE |
| 256 | rs77641763 | T | C | 15.8310 | -0.0110363 | 0.00185565 | 7.40E-10 | 534045 | 0.0193 | 0.0171 | 0.2593 | 484121 | TRUE | TRUE | TRUE |
| 257 | rs77848106 | A | C | 18.9795 | -0.00899002 | 0.00133197 | 2.10E-12 | 534045 | -0.0217 | 0.0086 | 0.0111099 | 484121 | TRUE | TRUE | TRUE |
| 258 | rs7810870 | G | C | 11.6983 | -0.00965481 | 0.00178828 | 4.30E-08 | 534045 | -0.0196 | 0.0283 | 0.489199 | 484121 | TRUE | TRUE | TRUE |
| 259 | rs78287937 | G | T | 12.6599 | 0.0127488 | 0.00212695 | 2.30E-09 | 534045 | -0.0196 | 0.0281 | 0.4851 | 484121 | TRUE | TRUE | TRUE |
| 260 | rs7925214 | T | C | 34.4965 | 0.00742042 | 0.00122889 | 1.00E-09 | 534045 | 8.00E-04 | 0.0085 | 0.921 | 484121 | TRUE | TRUE | TRUE |
| 261 | rs7952436 | T | C | 195.2531 | -0.032854 | 0.00221628 | 8.60E-51 | 534045 | 0.0126 | 0.0272 | 0.643101 | 484121 | TRUE | TRUE | TRUE |
| 262 | rs7969505 | C | G | 24.3143 | -0.0123727 | 0.00192369 | 3.40E-10 | 534045 | 0.0131 | 0.0214 | 0.541499 | 484121 | TRUE | TRUE | TRUE |
| 263 | rs7994814 | T | C | 18.3031 | 0.00911979 | 0.00125382 | 1.10E-12 | 534045 | -0.0128 | 0.0201 | 0.5232 | 484121 | TRUE | TRUE | TRUE |
| 264 | rs8002779 | A | G | 26.0324 | -0.00723785 | 0.00124315 | 5.40E-09 | 534045 | -0.0083 | 0.0084 | 0.3201 | 484121 | TRUE | TRUE | TRUE |
| 265 | rs8007644 | A | G | 68.4675 | 0.00763049 | 0.00126237 | 1.10E-09 | 534045 | -0.0051 | 0.0087 | 0.559 | 484121 | TRUE | TRUE | TRUE |
| 266 | rs8024244 | A | G | 75.2567 | 0.0103072 | 0.00141033 | 8.70E-13 | 534045 | 0.0064 | 0.009 | 0.4761 | 484121 | TRUE | TRUE | TRUE |
| 267 | rs8042545 | A | G | 42.8475 | 0.0120881 | 0.0014277 | 7.70E-18 | 534045 | -0.014 | 0.0113 | 0.216 | 484121 | TRUE | TRUE | TRUE |
| 268 | rs8059189 | A | G | 31.3559 | -0.0107879 | 0.00127348 | 1.20E-16 | 534045 | 0.0246 | 0.0205 | 0.2297 | 484121 | TRUE | TRUE | TRUE |
| 269 | rs8066863 | G | A | 39.5244 | -0.0175659 | 0.00191039 | 1.40E-19 | 534045 | 0.0349 | 0.0148 | 0.0181501 | 484121 | TRUE | TRUE | TRUE |
| 270 | rs8074074 | T | C | 17.1781 | 0.00899983 | 0.0014241 | 4.20E-11 | 534045 | -0.016 | 0.0092 | 0.0814592 | 484121 | TRUE | TRUE | TRUE |
| 271 | rs8132129 | T | C | 24.3339 | -0.00932601 | 0.00159425 | 1.10E-08 | 534045 | 0.0076 | 0.016 | 0.634 | 484121 | TRUE | TRUE | TRUE |
| 272 | rs822549 | C | T | 13.4674 | 0.0119515 | 0.0013736 | 5.80E-19 | 534045 | -0.003 | 0.0099 | 0.759799 | 484121 | TRUE | TRUE | TRUE |
| 273 | rs887912 | C | T | 28.9023 | -0.0110788 | 0.00132035 | 1.70E-17 | 534045 | -0.0068 | 0.0152 | 0.6531 | 484121 | TRUE | TRUE | TRUE |
| 274 | rs9327336 | C | T | 28.8933 | 0.0088433 | 0.00127657 | 5.80E-12 | 534045 | 0.0089 | 0.0085 | 0.2965 | 484121 | TRUE | TRUE | TRUE |
| 275 | rs9352808 | T | G | 35.2577 | -0.0114826 | 0.00121714 | 1.10E-20 | 534045 | -0.0079 | 0.0084 | 0.3452 | 484121 | TRUE | TRUE | TRUE |
| 276 | rs9398171 | T | C | 33.6535 | 0.0210115 | 0.00133717 | 1.80E-56 | 534045 | 0.0052 | 0.0094 | 0.5824 | 484121 | TRUE | TRUE | TRUE |
| 277 | rs9513510 | C | G | 166.8876 | -0.00902971 | 0.00132982 | 1.30E-11 | 534045 | 0.0021 | 0.0086 | 0.8069 | 484121 | TRUE | TRUE | TRUE |
| 278 | rs9568856 | A | G | 48.2824 | 0.0144239 | 0.00182656 | 3.40E-15 | 534045 | -0.0011 | 0.0109 | 0.9223 | 484121 | TRUE | TRUE | TRUE |
| 279 | rs9634212 | A | C | 19.2315 | 0.0226699 | 0.00146933 | 2.90E-54 | 534045 | 0.0142 | 0.0093 | 0.1296 | 484121 | TRUE | TRUE | TRUE |
| 280 | rs9654453 | C | T | 30.6801 | 0.0118361 | 0.00180909 | 1.80E-10 | 534045 | 0.0062 | 0.0174 | 0.722099 | 484121 | TRUE | TRUE | TRUE |
| 281 | rs9861443 | C | A | 29.3244 | 0.0110555 | 0.00134902 | 3.70E-16 | 534045 | 0.0037 | 0.0104 | 0.719399 | 484121 | TRUE | TRUE | TRUE |
| 282 | rs9888533 | T | C | 14.7613 | 0.00739921 | 0.00124552 | 1.10E-08 | 534045 | 0.0019 | 0.009 | 0.8365 | 484121 | TRUE | TRUE | TRUE |
| 283 | rs9888796 | C | T | 16.4620 | -0.00970134 | 0.00139427 | 1.10E-12 | 534045 | 5.00E-04 | 0.0118 | 0.9695 | 484121 | TRUE | TRUE | TRUE |
| 284 | rs9892365 | G | A | 62.3554 | -0.0150126 | 0.00129635 | 3.30E-30 | 534045 | 0.0188 | 0.0089 | 0.0354797 | 484121 | TRUE | TRUE | TRUE |
| 285 | rs9894577 | A | G | 53.7266 | -0.0136663 | 0.00131077 | 4.70E-25 | 534045 | -0.003 | 0.0103 | 0.769699 | 484121 | TRUE | TRUE | TRUE |
| 286 | rs9898189 | G | C | 13.1346 | -0.00800023 | 0.00132175 | 8.20E-10 | 534045 | -0.0048 | 0.0094 | 0.6089 | 484121 | TRUE | TRUE | TRUE |
| 287 | rs9960619 | T | C | 26.5002 | 0.00895586 | 0.0012853 | 6.10E-13 | 534045 | -0.0128 | 0.0112 | 0.252 | 484121 | TRUE | TRUE | TRUE |
| 288 | rs9989141 | T | C | 26.2594 | 0.00970164 | 0.00126785 | 3.20E-15 | 534045 | 0.0042 | 0.0097 | 0.6635 | 484121 | TRUE | TRUE | TRUE |
| 289 | rs10170971 | C | G | - | -0.00915477 | 0.0012123 | 1.90E-14 | 534045 | -0.0061 | 0.0092 | 0.5097 | 484121 | FALSE | TRUE | TRUE |
| 290 | rs1038196 | C | G | - | -0.0254899 | 0.00121538 | 5.50E-100 | 534045 | 0.0052 | 0.0102 | 0.6083 | 484121 | FALSE | TRUE | TRUE |
| 291 | rs10432304 | T | A | - | 0.00840196 | 0.00124786 | 2.50E-11 | 534045 | 0.0124 | 0.02 | 0.5346 | 484121 | FALSE | TRUE | TRUE |
| 292 | rs10791249 | A | T | - | 0.00721426 | 0.0012268 | 1.10E-08 | 534045 | -0.0057 | 0.0121 | 0.6339 | 484121 | FALSE | TRUE | TRUE |
| 293 | rs10883560 | G | C | - | 0.016215 | 0.00123213 | 2.50E-39 | 534045 | -0.0274 | 0.0121 | 0.0235299 | 484121 | FALSE | TRUE | TRUE |
| 294 | rs11234234 | A | T | - | -0.00725011 | 0.00122809 | 4.00E-09 | 534045 | -0.0019 | 0.0087 | 0.8294 | 484121 | FALSE | TRUE | TRUE |
| 295 | rs11695297 | A | T | - | 0.0070526 | 0.00124544 | 1.60E-08 | 534045 | -0.0131 | 0.0086 | 0.1264 | 484121 | FALSE | TRUE | TRUE |
| 296 | rs13047416 | G | C | - | -0.00882468 | 0.00126905 | 6.00E-13 | 534045 | -0.0046 | 0.0091 | 0.6131 | 484121 | FALSE | TRUE | TRUE |
| 297 | rs13264909 | T | A | - | -0.0093819 | 0.00123096 | 3.20E-14 | 534045 | -0.0139 | 0.0089 | 0.1175 | 484121 | FALSE | TRUE | TRUE |
| 298 | rs1478575 | A | T | - | 0.0163876 | 0.00130032 | 5.70E-36 | 534045 | -0.0046 | 0.0089 | 0.6008 | 484121 | FALSE | TRUE | TRUE |
| 299 | rs153758 | C | G | - | -0.00848413 | 0.00122575 | 5.10E-12 | 534045 | -0.0024 | 0.0084 | 0.774399 | 484121 | FALSE | TRUE | TRUE |
| 300 | rs2365363 | A | T | - | 0.00690397 | 0.00121578 | 4.90E-09 | 534045 | 0.002 | 0.0086 | 0.8189 | 484121 | FALSE | TRUE | TRUE |
| 301 | rs3749387 | C | G | - | 0.0107353 | 0.00122263 | 6.80E-19 | 534045 | 4.00E-04 | 0.0087 | 0.9664 | 484121 | FALSE | TRUE | TRUE |
| 302 | rs3771382 | G | C | - | -0.0114084 | 0.00122087 | 1.30E-19 | 534045 | -1.00E-04 | 0.009 | 0.9877 | 484121 | FALSE | TRUE | TRUE |
| 303 | rs3796232 | C | G | - | -0.0072872 | 0.00121793 | 1.40E-09 | 534045 | 0.0035 | 0.0136 | 0.7976 | 484121 | FALSE | TRUE | TRUE |
| 304 | rs380740 | A | T | - | 0.0106557 | 0.00121805 | 1.40E-18 | 534045 | 0.003 | 0.0085 | 0.723601 | 484121 | FALSE | TRUE | TRUE |
| 305 | rs3807949 | T | A | - | 0.00697938 | 0.0012183 | 1.40E-08 | 534045 | 0.0104 | 0.0089 | 0.2422 | 484121 | FALSE | TRUE | TRUE |
| 306 | rs4677150 | G | C | - | -0.00730914 | 0.00121769 | 1.10E-09 | 534045 | 0.0099 | 0.0085 | 0.2481 | 484121 | FALSE | TRUE | TRUE |
| 307 | rs4776970 | T | A | - | -0.0130164 | 0.0012736 | 3.70E-26 | 534045 | -0.0021 | 0.0095 | 0.8273 | 484121 | FALSE | TRUE | TRUE |
| 308 | rs4899012 | C | G | - | -0.0209835 | 0.00125084 | 3.90E-64 | 534045 | -0.0114 | 0.0095 | 0.2284 | 484121 | FALSE | TRUE | TRUE |
| 309 | rs4973593 | G | C | - | -0.00701488 | 0.00120663 | 6.20E-09 | 534045 | 0.012 | 0.0085 | 0.1576 | 484121 | FALSE | TRUE | TRUE |
| 310 | rs58857770 | G | C | - | -0.00915724 | 0.00124905 | 7.50E-14 | 534045 | -0.0099 | 0.0085 | 0.2473 | 484121 | FALSE | TRUE | TRUE |
| 311 | rs62396185 | C | G | - | -0.0179794 | 0.00150597 | 5.70E-33 | 534045 | -0.0171 | 0.0104 | 0.0994191 | 484121 | FALSE | TRUE | TRUE |
| 312 | rs6597975 | G | C | - | 0.00738407 | 0.0012297 | 4.90E-09 | 534045 | -0.0083 | 0.0089 | 0.35 | 484121 | FALSE | TRUE | TRUE |
| 313 | rs773145 | C | G | - | -0.00699917 | 0.00123601 | 3.10E-09 | 534045 | 5.00E-04 | 0.0087 | 0.9503 | 484121 | FALSE | TRUE | TRUE |
| 314 | rs7916441 | C | G | - | -0.00807459 | 0.00122824 | 2.30E-10 | 534045 | -0.0088 | 0.0087 | 0.3122 | 484121 | FALSE | TRUE | TRUE |
| 315 | rs7942152 | A | T | - | -0.0101814 | 0.00122468 | 3.80E-16 | 534045 | 0.0075 | 0.0085 | 0.3752 | 484121 | FALSE | TRUE | TRUE |
| 316 | rs9976812 | G | C | - | -0.0139731 | 0.00124629 | 2.80E-29 | 534045 | 0.0044 | 0.0091 | 0.6242 | 484121 | FALSE | TRUE | TRUE |
| 317 | rs10020631 | A | G | - | -0.00846262 | 0.00141405 | 2.90E-09 | 534045 | 0.0108 | 0.0126 | 0.3924 | 484121 | FALSE | TRUE | TRUE |
| 318 | rs10145154 | T | C | - | 0.017176 | 0.00147103 | 1.60E-32 | 534045 | -0.0104 | 0.016 | 0.5164 | 484121 | FALSE | TRUE | TRUE |
| 319 | rs10269774 | A | G | - | 0.0251585 | 0.00129714 | 1.10E-83 | 534045 | -0.0238 | 0.0113 | 0.03434 | 484121 | FALSE | TRUE | TRUE |
| 320 | rs10282707 | T | C | - | 0.00753287 | 0.00124209 | 1.10E-08 | 534045 | 0.0086 | 0.0085 | 0.3146 | 484121 | FALSE | TRUE | TRUE |
| 321 | rs10404726 | T | C | - | -0.00954329 | 0.00122499 | 4.50E-16 | 534045 | -3.00E-04 | 0.0114 | 0.9762 | 484121 | FALSE | TRUE | TRUE |
| 322 | rs1047891 | A | C | - | 0.0156861 | 0.00129669 | 5.20E-34 | 534045 | -0.0144 | 0.0106 | 0.1736 | 484121 | FALSE | TRUE | TRUE |
| 323 | rs10020631 | A | G | - | -0.00846262 | 0.00141405 | 2.90E-09 | 534045 | 0.0108 | 0.0126 | 0.3924 | 484121 | TRUE | FALSE | TRUE |
| 324 | rs10145154 | T | C | - | 0.017176 | 0.00147103 | 1.60E-32 | 534045 | -0.0104 | 0.016 | 0.5164 | 484121 | TRUE | FALSE | TRUE |
| 325 | rs10269774 | A | G | - | 0.0251585 | 0.00129714 | 1.10E-83 | 534045 | -0.0238 | 0.0113 | 0.03434 | 484121 | TRUE | FALSE | TRUE |
| 326 | rs10282707 | T | C | - | 0.00753287 | 0.00124209 | 1.10E-08 | 534045 | 0.0086 | 0.0085 | 0.3146 | 484121 | TRUE | FALSE | TRUE |
| 327 | rs10404726 | T | C | - | -0.00954329 | 0.00122499 | 4.50E-16 | 534045 | -3.00E-04 | 0.0114 | 0.9762 | 484121 | TRUE | FALSE | TRUE |
| 328 | rs1047891 | A | C | - | 0.0156861 | 0.00129669 | 5.20E-34 | 534045 | -0.0144 | 0.0106 | 0.1736 | 484121 | TRUE | FALSE | TRUE |
| 329 | rs10767659 | T | G | - | -0.0184382 | 0.00129493 | 3.50E-47 | 534045 | -0.0056 | 0.0094 | 0.551099 | 484121 | TRUE | FALSE | TRUE |
| 330 | rs10775348 | G | A | - | 0.0123402 | 0.00133633 | 5.80E-20 | 534045 | -0.0042 | 0.0128 | 0.7405 | 484121 | TRUE | FALSE | TRUE |
| 331 | rs10775406 | G | A | - | -0.0108954 | 0.00142609 | 1.40E-14 | 534045 | -0.029 | 0.0156 | 0.0633505 | 484121 | TRUE | FALSE | TRUE |
| 332 | rs1121 | A | G | - | -0.011556 | 0.00133127 | 3.20E-18 | 534045 | -0.0275 | 0.0086 | 0.001438 | 484121 | TRUE | FALSE | TRUE |
| 333 | rs12140153 | T | G | - | -0.0215916 | 0.00211615 | 2.40E-25 | 534045 | -0.0404 | 0.0244 | 0.09794 | 484121 | TRUE | FALSE | TRUE |
| 334 | rs1260326 | C | T | - | 0.018366 | 0.00123351 | 1.30E-49 | 534045 | -7.00E-04 | 0.0085 | 0.9354 | 484121 | TRUE | FALSE | TRUE |
| 335 | rs12888955 | A | G | - | -0.0093836 | 0.00128345 | 2.50E-14 | 534045 | -0.0061 | 0.0085 | 0.4715 | 484121 | TRUE | FALSE | TRUE |
| 336 | rs1412234 | C | T | - | 0.0127902 | 0.00130134 | 6.50E-24 | 534045 | 0.0054 | 0.0098 | 0.5829 | 484121 | TRUE | FALSE | TRUE |
| 337 | rs17491275 | G | T | - | 0.015078 | 0.00166102 | 8.90E-20 | 534045 | -0.0052 | 0.0111 | 0.6404 | 484121 | TRUE | FALSE | TRUE |
| 338 | rs2102278 | G | A | - | 0.0113309 | 0.00130218 | 9.80E-20 | 534045 | 0.0233 | 0.0087 | 0.00738193 | 484121 | TRUE | FALSE | TRUE |
| 339 | rs2280141 | G | T | - | 0.0118855 | 0.00121871 | 4.60E-22 | 534045 | -0.0072 | 0.0085 | 0.3949 | 484121 | TRUE | FALSE | TRUE |
| 340 | rs2284932 | A | G | - | 0.00709367 | 0.00123694 | 8.00E-09 | 534045 | -0.006 | 0.0087 | 0.4912 | 484121 | TRUE | FALSE | TRUE |
| 341 | rs2307111 | C | T | - | -0.0180158 | 0.00124155 | 1.30E-47 | 534045 | -2.00E-04 | 0.0085 | 0.9857 | 484121 | TRUE | FALSE | TRUE |
| 342 | rs2678204 | G | T | - | 0.0133508 | 0.00127583 | 2.90E-26 | 534045 | -0.0043 | 0.0113 | 0.702999 | 484121 | TRUE | FALSE | TRUE |
| 343 | rs2812208 | C | G | - | 0.0633463 | 0.00426521 | 9.80E-51 | 534045 | 0.0648 | 0.047 | 0.1677 | 484121 | TRUE | FALSE | TRUE |
| 344 | rs35506085 | A | G | - | -0.0186299 | 0.00158145 | 9.00E-31 | 534045 | -0.0107 | 0.0092 | 0.2458 | 484121 | TRUE | FALSE | TRUE |
| 345 | rs35626515 | A | C | - | 0.0129766 | 0.00124043 | 1.60E-26 | 534045 | -0.0109 | 0.0095 | 0.2502 | 484121 | TRUE | FALSE | TRUE |
| 346 | rs35665085 | A | G | - | -0.0169576 | 0.00266141 | 1.20E-10 | 534045 | 0.0474 | 0.0298 | 0.1117 | 484121 | TRUE | FALSE | TRUE |
| 347 | rs4369382 | G | A | - | -0.0102317 | 0.00138473 | 6.50E-14 | 534045 | 0.0131 | 0.0087 | 0.1296 | 484121 | TRUE | FALSE | TRUE |
| 348 | rs4588572 | G | A | - | 0.00933806 | 0.00144553 | 2.90E-11 | 534045 | -0.0011 | 0.0123 | 0.9264 | 484121 | TRUE | FALSE | TRUE |
| 349 | rs4715207 | T | C | - | 0.0221752 | 0.00157953 | 1.70E-45 | 534045 | 0.0094 | 0.0104 | 0.3642 | 484121 | TRUE | FALSE | TRUE |
| 350 | rs55831773 | T | C | - | -0.0150691 | 0.00155619 | 5.10E-22 | 534045 | -0.0213 | 0.0131 | 0.1048 | 484121 | TRUE | FALSE | TRUE |
| 351 | rs56094641 | G | A | - | 0.0412245 | 0.00123984 | 1.00E-200 | 534045 | 0.0081 | 0.0095 | 0.3948 | 484121 | TRUE | FALSE | TRUE |
| 352 | rs5753630 | G | A | - | 0.00784561 | 0.00123883 | 5.20E-11 | 534045 | -0.0049 | 0.0105 | 0.641201 | 484121 | TRUE | FALSE | TRUE |
| 353 | rs6124249 | C | T | - | 0.0074819 | 0.00133679 | 4.10E-09 | 534045 | 0.0022 | 0.0087 | 0.7971 | 484121 | TRUE | FALSE | TRUE |
| 354 | rs62621812 | A | G | - | 0.0385148 | 0.00442777 | 2.90E-18 | 534045 | -0.018 | 0.0388 | 0.6434 | 484121 | TRUE | FALSE | TRUE |
| 355 | rs6567160 | C | T | - | 0.0449666 | 0.00144504 | 1.00E-200 | 534045 | 0.0127 | 0.0102 | 0.2154 | 484121 | TRUE | FALSE | TRUE |
| 356 | rs7080472 | T | G | - | 0.0109773 | 0.00123499 | 9.10E-19 | 534045 | -0.0359 | 0.0086 | 2.84E-05 | 484121 | TRUE | FALSE | TRUE |
| 357 | rs7132908 | A | G | - | 0.0172948 | 0.0012484 | 1.00E-45 | 534045 | -1.00E-04 | 0.009 | 0.9925 | 484121 | TRUE | FALSE | TRUE |
| 358 | rs73004967 | G | A | - | -0.0177547 | 0.00242315 | 4.40E-13 | 534045 | 0.0141 | 0.0275 | 0.6084 | 484121 | TRUE | FALSE | TRUE |
| 359 | rs76895963 | G | T | - | 0.0954736 | 0.00470476 | 1.20E-92 | 534045 | -0.0642 | 0.045 | 0.1536 | 484121 | TRUE | FALSE | TRUE |
| 360 | rs78378222 | G | T | - | 0.07743 | 0.00563246 | 1.80E-44 | 534045 | 0.0225 | 0.057 | 0.6925 | 484121 | TRUE | FALSE | TRUE |
| 361 | rs9379084 | A | G | - | -0.0147219 | 0.00195388 | 3.80E-14 | 534045 | 0.0081 | 0.0109 | 0.4588 | 484121 | TRUE | FALSE | TRUE |
| 362 | rs9388498 | T | G | - | 0.017573 | 0.00159981 | 5.60E-28 | 534045 | -0.0193 | 0.0259 | 0.4573 | 484121 | TRUE | FALSE | TRUE |
| 363 | rs9496614 | C | T | - | 0.00907259 | 0.00141328 | 7.90E-11 | 534045 | -0.0126 | 0.0163 | 0.4415 | 484121 | TRUE | FALSE | TRUE |
| 364 | rs9564268 | C | T | - | -0.00829326 | 0.00125828 | 8.40E-11 | 534045 | 0.0061 | 0.0112 | 0.5872 | 484121 | TRUE | FALSE | TRUE |
| 365 | rs10124197 | C | T | - | -0.00708775 | 0.00132487 | 4.00E-08 | 534045 | 0.006 | 0.0149 | 0.6892 | 484121 | TRUE | TRUE | FALSE |
| 366 | rs10215645 | C | T | - | 0.00793283 | 0.00122103 | 7.70E-11 | 534045 | -0.0068 | 0.0093 | 0.462 | 484121 | TRUE | TRUE | FALSE |
| 367 | rs10243988 | A | G | - | -0.00909182 | 0.00153622 | 1.70E-08 | 534045 | 0.0235 | 0.0124 | 0.0572902 | 484121 | TRUE | TRUE | FALSE |
| 368 | rs1035607 | C | A | - | 0.00898685 | 0.00141981 | 3.40E-10 | 534045 | 9.00E-04 | 0.0089 | 0.9227 | 484121 | TRUE | TRUE | FALSE |
| 369 | rs1060967 | C | G | - | -0.00816335 | 0.00126479 | 2.30E-11 | 534045 | -0.0025 | 0.0096 | 0.7907 | 484121 | TRUE | TRUE | FALSE |
| 370 | rs10769939 | T | C | - | -0.00911419 | 0.00128219 | 7.20E-13 | 534045 | -0.0076 | 0.0087 | 0.3837 | 484121 | TRUE | TRUE | FALSE |
| 371 | rs10773172 | A | G | - | 0.0173355 | 0.00138851 | 1.60E-36 | 534045 | -0.0188 | 0.0111 | 0.0909097 | 484121 | TRUE | TRUE | FALSE |
| 372 | rs10803955 | G | A | - | -0.010769 | 0.00120505 | 3.20E-19 | 534045 | 0.0052 | 0.0093 | 0.5784 | 484121 | TRUE | TRUE | FALSE |
| 373 | rs10811668 | A | C | - | -0.00879786 | 0.00151909 | 2.50E-09 | 534045 | -0.0139 | 0.01 | 0.1652 | 484121 | TRUE | TRUE | FALSE |
| 374 | rs10835368 | C | T | - | 0.00910497 | 0.00125782 | 1.70E-13 | 534045 | 0.0085 | 0.0091 | 0.3533 | 484121 | TRUE | TRUE | FALSE |
| 375 | rs10958478 | T | A | - | 0.0180477 | 0.00145531 | 8.60E-36 | 534045 | -0.003 | 0.0104 | 0.7738 | 484121 | TRUE | TRUE | FALSE |
| 376 | rs10973198 | C | T | - | -0.00775301 | 0.00121899 | 9.70E-11 | 534045 | 0.0061 | 0.0086 | 0.4804 | 484121 | TRUE | TRUE | FALSE |
| 377 | rs10979612 | C | T | - | 0.0179176 | 0.00233872 | 1.90E-15 | 534045 | 0.0022 | 0.0131 | 0.8699 | 484121 | TRUE | TRUE | FALSE |
| 378 | rs10985962 | T | C | - | -0.00720236 | 0.00127069 | 7.00E-09 | 534045 | -0.0084 | 0.0089 | 0.3448 | 484121 | TRUE | TRUE | FALSE |
| 379 | rs10995319 | C | T | - | -0.00865181 | 0.0014387 | 1.60E-09 | 534045 | 0.0019 | 0.0121 | 0.8726 | 484121 | TRUE | TRUE | FALSE |
| 380 | rs111365325 | T | C | - | -0.0133059 | 0.00144045 | 2.20E-20 | 534045 | -0.0033 | 0.0118 | 0.777 | 484121 | TRUE | TRUE | FALSE |
| 381 | rs11142705 | A | G | - | -0.00798757 | 0.00124142 | 1.20E-10 | 534045 | -0.0126 | 0.0085 | 0.139 | 484121 | TRUE | TRUE | FALSE |
| 382 | rs1122848 | G | A | - | -0.00724958 | 0.00130665 | 1.70E-08 | 534045 | -0.0266 | 0.0095 | 0.00517297 | 484121 | TRUE | TRUE | FALSE |
| 383 | rs11263862 | T | C | - | 0.00794387 | 0.00131018 | 3.40E-09 | 534045 | -0.008 | 0.0086 | 0.3561 | 484121 | TRUE | TRUE | FALSE |
| 384 | rs112685832 | A | C | - | 0.014217 | 0.00191907 | 1.00E-13 | 534045 | 0.001 | 0.0226 | 0.9663 | 484121 | TRUE | TRUE | FALSE |
| 385 | rs113115311 | A | C | - | -0.0128855 | 0.00236902 | 2.90E-08 | 534045 | -0.0061 | 0.0147 | 0.676401 | 484121 | TRUE | TRUE | FALSE |
| 386 | rs114177791 | T | G | - | 0.0113071 | 0.00148728 | 1.70E-14 | 534045 | -0.0149 | 0.0168 | 0.3763 | 484121 | TRUE | TRUE | FALSE |
| 387 | rs11498196 | T | C | - | 0.00791775 | 0.00124142 | 2.10E-10 | 534045 | -0.0219 | 0.0085 | 0.01037 | 484121 | TRUE | TRUE | FALSE |
| 388 | rs11524516 | A | G | - | -0.010174 | 0.00133659 | 2.20E-15 | 534045 | 0.0194 | 0.0124 | 0.1179 | 484121 | TRUE | TRUE | FALSE |
| 389 | rs11593107 | T | C | - | -0.00792108 | 0.00122311 | 5.60E-10 | 534045 | 0.0161 | 0.0086 | 0.061 | 484121 | TRUE | TRUE | FALSE |
| 390 | rs11616283 | C | T | - | 0.0113507 | 0.00178826 | 1.20E-10 | 534045 | -0.0123 | 0.0211 | 0.5593 | 484121 | TRUE | TRUE | FALSE |
| 391 | rs116337081 | T | C | - | 0.0136505 | 0.00237141 | 3.70E-09 | 534045 | -0.0219 | 0.0292 | 0.4536 | 484121 | TRUE | TRUE | FALSE |
| 392 | rs11663903 | A | G | - | 0.00717217 | 0.00123663 | 1.90E-09 | 534045 | -0.0089 | 0.0199 | 0.6552 | 484121 | TRUE | TRUE | FALSE |
| 393 | rs1167309 | T | C | - | -0.0104944 | 0.00129452 | 2.00E-16 | 534045 | -0.0047 | 0.0087 | 0.592899 | 484121 | TRUE | TRUE | FALSE |
| 394 | rs11712872 | A | G | - | 0.0170954 | 0.00189283 | 2.50E-19 | 534045 | 0.0132 | 0.016 | 0.4089 | 484121 | TRUE | TRUE | FALSE |
| 395 | rs11739036 | A | G | - | -0.0117189 | 0.00131851 | 3.50E-18 | 534045 | 0.0025 | 0.01 | 0.8011 | 484121 | TRUE | TRUE | FALSE |
| 396 | rs117451679 | G | A | - | 0.0133981 | 0.00200104 | 3.80E-11 | 534045 | -0.0086 | 0.0213 | 0.685001 | 484121 | TRUE | TRUE | FALSE |
| 397 | rs1176314 | G | T | - | -0.00704636 | 0.00123396 | 5.60E-09 | 534045 | -0.001 | 0.0084 | 0.907 | 484121 | TRUE | TRUE | FALSE |
| 398 | rs11771928 | A | G | - | -0.00749689 | 0.00133365 | 4.10E-09 | 534045 | -0.0166 | 0.0121 | 0.1712 | 484121 | TRUE | TRUE | FALSE |
| 399 | rs11773731 | G | A | - | 0.0116689 | 0.00126071 | 2.30E-20 | 534045 | 0.016 | 0.0086 | 0.0611604 | 484121 | TRUE | TRUE | FALSE |
| 400 | rs11784985 | T | A | - | -0.00834207 | 0.00124208 | 3.60E-12 | 534045 | -0.0066 | 0.0112 | 0.552699 | 484121 | TRUE | TRUE | FALSE |
| 401 | rs11833839 | T | C | - | 0.0169207 | 0.00262949 | 1.10E-10 | 534045 | -0.0012 | 0.0124 | 0.9225 | 484121 | TRUE | TRUE | FALSE |
| 402 | rs11925245 | G | A | - | -0.0107224 | 0.00158489 | 8.00E-12 | 534045 | -0.0107 | 0.0091 | 0.2403 | 484121 | TRUE | TRUE | FALSE |
| 403 | rs11997525 | A | T | - | 0.0135707 | 0.00163554 | 1.90E-16 | 534045 | 0.0164 | 0.0103 | 0.113 | 484121 | TRUE | TRUE | FALSE |
| 404 | rs12031493 | G | A | - | -0.00829079 | 0.00121424 | 2.90E-12 | 534045 | 0.0085 | 0.0088 | 0.3298 | 484121 | TRUE | TRUE | FALSE |
| 405 | rs12041740 | A | G | - | -0.0155671 | 0.00137651 | 1.70E-29 | 534045 | -0.0046 | 0.0101 | 0.6524 | 484121 | TRUE | TRUE | FALSE |
| 406 | rs12091972 | C | A | - | 0.0222206 | 0.00211016 | 3.30E-26 | 534045 | -0.0097 | 0.0156 | 0.5346 | 484121 | TRUE | TRUE | FALSE |
| 407 | rs12148418 | A | G | - | -0.0104214 | 0.001224 | 3.60E-18 | 534045 | 0.0014 | 0.0085 | 0.8696 | 484121 | TRUE | TRUE | FALSE |
| 408 | rs12188627 | G | A | - | -0.0107869 | 0.00121316 | 4.20E-19 | 534045 | -0.0243 | 0.0102 | 0.01689 | 484121 | TRUE | TRUE | FALSE |
| 409 | rs12213070 | A | G | - | -0.00930116 | 0.00127337 | 3.80E-13 | 534045 | -0.0074 | 0.0091 | 0.4153 | 484121 | TRUE | TRUE | FALSE |
| 410 | rs12227680 | A | T | - | 0.00764283 | 0.00137898 | 3.90E-08 | 534045 | 0.002 | 0.0147 | 0.8912 | 484121 | TRUE | TRUE | FALSE |
| 411 | rs12434837 | T | C | - | -0.0116772 | 0.00197247 | 1.10E-08 | 534045 | 0.0134 | 0.0154 | 0.3867 | 484121 | TRUE | TRUE | FALSE |
| 412 | rs12520308 | C | T | - | -0.00907289 | 0.0012978 | 9.80E-13 | 534045 | -0.0052 | 0.0087 | 0.5514 | 484121 | TRUE | TRUE | FALSE |
| 413 | rs12520894 | C | T | - | 0.00667023 | 0.00121938 | 9.10E-09 | 534045 | -0.0112 | 0.0111 | 0.3146 | 484121 | TRUE | TRUE | FALSE |
| 414 | rs12533548 | G | A | - | 0.00729168 | 0.00127963 | 8.10E-09 | 534045 | 1.00E-04 | 0.0091 | 0.9937 | 484121 | TRUE | TRUE | FALSE |
| 415 | rs12543207 | T | C | - | -0.00982666 | 0.00143741 | 2.10E-12 | 534045 | -0.0048 | 0.0087 | 0.5768 | 484121 | TRUE | TRUE | FALSE |
| 416 | rs12588830 | A | G | - | 0.0100055 | 0.00157846 | 1.40E-10 | 534045 | 0.0056 | 0.009 | 0.5331 | 484121 | TRUE | TRUE | FALSE |
| 417 | rs12657771 | A | G | - | -0.0117786 | 0.00122852 | 6.80E-22 | 534045 | -0.0026 | 0.0085 | 0.7621 | 484121 | TRUE | TRUE | FALSE |
| 418 | rs12679359 | T | G | - | -0.0122436 | 0.00177134 | 2.70E-12 | 534045 | 0.0033 | 0.0094 | 0.7278 | 484121 | TRUE | TRUE | FALSE |
| 419 | rs12729817 | G | A | - | -0.00869965 | 0.00120729 | 1.80E-12 | 534045 | 0.0205 | 0.0084 | 0.01439 | 484121 | TRUE | TRUE | FALSE |
| 420 | rs12784071 | T | C | - | 0.00898176 | 0.00144751 | 1.40E-09 | 534045 | 0.0164 | 0.0136 | 0.2282 | 484121 | TRUE | TRUE | FALSE |
| 421 | rs12881375 | A | C | - | -0.00709574 | 0.00134994 | 3.10E-08 | 534045 | -0.0037 | 0.0095 | 0.696899 | 484121 | TRUE | TRUE | FALSE |
| 422 | rs12900800 | A | G | - | -0.00900092 | 0.0014203 | 8.10E-11 | 534045 | 0.0196 | 0.0151 | 0.1941 | 484121 | TRUE | TRUE | FALSE |
| 423 | rs12908182 | T | C | - | -0.0102749 | 0.0012226 | 3.40E-16 | 534045 | 0.0128 | 0.0085 | 0.1319 | 484121 | TRUE | TRUE | FALSE |
| 424 | rs12959994 | A | G | - | -0.0115206 | 0.00146637 | 1.80E-15 | 534045 | -0.0031 | 0.0093 | 0.736801 | 484121 | TRUE | TRUE | FALSE |
| 425 | rs12981554 | G | A | - | -0.00891311 | 0.00123404 | 8.20E-13 | 534045 | -0.0055 | 0.0087 | 0.5281 | 484121 | TRUE | TRUE | FALSE |
| 426 | rs13007086 | T | A | - | 0.0362174 | 0.0015995 | 7.80E-116 | 534045 | 0.003 | 0.0127 | 0.8129 | 484121 | TRUE | TRUE | FALSE |
| 427 | rs13077747 | C | T | - | 0.0131083 | 0.0012139 | 4.60E-27 | 534045 | -0.0177 | 0.0087 | 0.0407803 | 484121 | TRUE | TRUE | FALSE |
| 428 | rs13099930 | G | A | - | -0.00864195 | 0.00128509 | 1.70E-11 | 534045 | -0.0026 | 0.0104 | 0.8061 | 484121 | TRUE | TRUE | FALSE |
| 429 | rs13125807 | T | C | - | 0.0113994 | 0.00174859 | 1.50E-11 | 534045 | 0.003 | 0.0106 | 0.7756 | 484121 | TRUE | TRUE | FALSE |
| 430 | rs13173394 | C | T | - | 0.00736701 | 0.00125897 | 5.90E-09 | 534045 | -0.0025 | 0.0085 | 0.7651 | 484121 | TRUE | TRUE | FALSE |
| 431 | rs13176855 | T | C | - | -0.00645232 | 0.00123293 | 4.60E-08 | 534045 | 0.0132 | 0.0099 | 0.1832 | 484121 | TRUE | TRUE | FALSE |
| 432 | rs13244614 | A | C | - | 0.0116781 | 0.00134935 | 1.20E-18 | 534045 | 0.0246 | 0.0116 | 0.0332698 | 484121 | TRUE | TRUE | FALSE |
| 433 | rs13299559 | T | C | - | -0.00949077 | 0.00122514 | 4.50E-14 | 534045 | 0.0167 | 0.0113 | 0.1397 | 484121 | TRUE | TRUE | FALSE |
| 434 | rs13392666 | A | T | - | 0.00805374 | 0.00145575 | 1.80E-08 | 534045 | -0.0024 | 0.0158 | 0.8797 | 484121 | TRUE | TRUE | FALSE |
| 435 | rs1374370 | A | G | - | 0.00892898 | 0.00131109 | 1.60E-11 | 534045 | -0.0062 | 0.0123 | 0.616601 | 484121 | TRUE | TRUE | FALSE |
| 436 | rs139031896 | C | A | - | -0.021306 | 0.003723 | 7.10E-09 | 534045 | 0.0105 | 0.0433 | 0.8085 | 484121 | TRUE | TRUE | FALSE |
| 437 | rs1421035 | T | C | - | 0.00768281 | 0.00128788 | 1.60E-09 | 534045 | -0.0086 | 0.0087 | 0.3219 | 484121 | TRUE | TRUE | FALSE |
| 438 | rs1424371 | T | C | - | 0.00682779 | 0.00127261 | 2.90E-08 | 534045 | 0.0026 | 0.0085 | 0.763199 | 484121 | TRUE | TRUE | FALSE |
| 439 | rs1431663 | C | A | - | -0.0104291 | 0.00123388 | 2.70E-17 | 534045 | -0.0078 | 0.0138 | 0.570401 | 484121 | TRUE | TRUE | FALSE |
| 440 | rs144260843 | A | G | - | -0.0231757 | 0.00401798 | 5.90E-09 | 534045 | 0.0565 | 0.0507 | 0.2655 | 484121 | TRUE | TRUE | FALSE |
| 441 | rs1517248 | G | A | - | -0.00757752 | 0.00127431 | 3.60E-09 | 534045 | 0.0052 | 0.014 | 0.7089 | 484121 | TRUE | TRUE | FALSE |
| 442 | rs1539110 | T | C | - | 0.00817826 | 0.00141399 | 1.90E-09 | 534045 | -0.0236 | 0.0155 | 0.1276 | 484121 | TRUE | TRUE | FALSE |
| 443 | rs1553065 | A | G | - | -0.00767302 | 0.00122929 | 2.40E-10 | 534045 | -0.009 | 0.0087 | 0.2971 | 484121 | TRUE | TRUE | FALSE |
| 444 | rs1618407 | C | T | - | 0.0118004 | 0.00156053 | 2.90E-15 | 534045 | 0.007 | 0.0091 | 0.4436 | 484121 | TRUE | TRUE | FALSE |
| 445 | rs1657222 | A | G | - | 0.00850909 | 0.00124828 | 1.30E-11 | 534045 | -0.0017 | 0.0084 | 0.8417 | 484121 | TRUE | TRUE | FALSE |
| 446 | rs17042011 | T | C | - | 0.00918505 | 0.00151809 | 9.00E-10 | 534045 | 0.0139 | 0.0103 | 0.1762 | 484121 | TRUE | TRUE | FALSE |
| 447 | rs17197114 | C | T | - | 0.0128264 | 0.00161007 | 1.50E-15 | 534045 | 0.0138 | 0.0148 | 0.3514 | 484121 | TRUE | TRUE | FALSE |
| 448 | rs1722322 | G | T | - | -0.00800814 | 0.00137673 | 2.30E-09 | 534045 | -0.0115 | 0.0092 | 0.2101 | 484121 | TRUE | TRUE | FALSE |
| 449 | rs1765133 | T | C | - | -0.00984288 | 0.00145352 | 5.10E-11 | 534045 | -0.0054 | 0.0105 | 0.6084 | 484121 | TRUE | TRUE | FALSE |
| 450 | rs17708997 | G | A | - | 0.0144055 | 0.00193082 | 2.80E-14 | 534045 | 0.0029 | 0.0179 | 0.8724 | 484121 | TRUE | TRUE | FALSE |
| 451 | rs17881236 | A | G | - | -0.0202624 | 0.00363006 | 4.00E-08 | 534045 | 0.0033 | 0.0448 | 0.9411 | 484121 | TRUE | TRUE | FALSE |
| 452 | rs1813212 | G | A | - | -0.0084891 | 0.0012263 | 2.60E-12 | 534045 | 0.0052 | 0.0086 | 0.5406 | 484121 | TRUE | TRUE | FALSE |
| 453 | rs1846221 | C | G | - | -0.00946551 | 0.00162378 | 5.60E-09 | 534045 | -0.0134 | 0.0156 | 0.3895 | 484121 | TRUE | TRUE | FALSE |
| 454 | rs1883798 | G | A | - | -0.00859234 | 0.00138127 | 1.70E-10 | 534045 | -0.0286 | 0.0151 | 0.0586003 | 484121 | TRUE | TRUE | FALSE |
| 455 | rs1910466 | C | T | - | -0.00833396 | 0.00121925 | 2.10E-11 | 534045 | 0.0174 | 0.0084 | 0.0388401 | 484121 | TRUE | TRUE | FALSE |
| 456 | rs1919442 | G | A | - | -0.00867775 | 0.00142386 | 1.20E-09 | 534045 | 0.0066 | 0.012 | 0.5818 | 484121 | TRUE | TRUE | FALSE |
| 457 | rs1960268 | T | G | - | 0.00782713 | 0.00139994 | 5.10E-09 | 534045 | 0.0088 | 0.01 | 0.3808 | 484121 | TRUE | TRUE | FALSE |
| 458 | rs1971955 | G | A | - | -0.0128869 | 0.00172333 | 1.70E-14 | 534045 | -0.0012 | 0.0094 | 0.9001 | 484121 | TRUE | TRUE | FALSE |
| 459 | rs2025129 | G | A | - | -0.00854624 | 0.00143047 | 6.10E-09 | 534045 | 0.0071 | 0.0131 | 0.584901 | 484121 | TRUE | TRUE | FALSE |
| 460 | rs2045767 | G | T | - | -0.00747892 | 0.00123685 | 4.90E-09 | 534045 | -0.0149 | 0.0088 | 0.0919306 | 484121 | TRUE | TRUE | FALSE |
| 461 | rs204928 | G | A | - | -0.00834644 | 0.00121828 | 5.20E-12 | 534045 | -0.0012 | 0.0087 | 0.8947 | 484121 | TRUE | TRUE | FALSE |
| 462 | rs2065999 | C | T | - | -0.00710784 | 0.00125604 | 5.40E-09 | 534045 | 0.004 | 0.0114 | 0.7258 | 484121 | TRUE | TRUE | FALSE |
| 463 | rs212538 | C | A | - | 0.00785849 | 0.001219 | 6.30E-11 | 534045 | -0.0056 | 0.0101 | 0.576901 | 484121 | TRUE | TRUE | FALSE |
| 464 | rs214247 | C | T | - | -0.00869719 | 0.00124966 | 1.20E-12 | 534045 | 0.0173 | 0.0096 | 0.0715007 | 484121 | TRUE | TRUE | FALSE |
| 465 | rs2163832 | C | T | - | -0.0126765 | 0.00129077 | 1.60E-22 | 534045 | 0.0299 | 0.0147 | 0.0420097 | 484121 | TRUE | TRUE | FALSE |
| 466 | rs2197563 | A | G | - | 0.010305 | 0.00123172 | 2.40E-16 | 534045 | -0.0311 | 0.0092 | 0.000753095 | 484121 | TRUE | TRUE | FALSE |
| 467 | rs2197780 | C | T | - | 0.0117034 | 0.00134559 | 2.90E-18 | 534045 | -0.0127 | 0.0094 | 0.1794 | 484121 | TRUE | TRUE | FALSE |
| 468 | rs2214964 | T | C | - | -0.00714483 | 0.00122226 | 2.00E-08 | 534045 | 0.0227 | 0.0088 | 0.00972389 | 484121 | TRUE | TRUE | FALSE |
| 469 | rs223942 | G | A | - | -0.00873474 | 0.00124814 | 1.30E-12 | 534045 | -0.0083 | 0.0086 | 0.3357 | 484121 | TRUE | TRUE | FALSE |
| 470 | rs2281175 | C | T | - | 0.0113782 | 0.00129339 | 7.40E-19 | 534045 | -0.0051 | 0.0093 | 0.582801 | 484121 | TRUE | TRUE | FALSE |
| 471 | rs2286028 | C | G | - | 0.0165585 | 0.00151626 | 2.70E-29 | 534045 | 0.016 | 0.0129 | 0.2168 | 484121 | TRUE | TRUE | FALSE |
| 472 | rs2320731 | T | C | - | 0.00826623 | 0.00130254 | 9.90E-11 | 534045 | 0.0077 | 0.0094 | 0.4113 | 484121 | TRUE | TRUE | FALSE |
| 473 | rs2358947 | G | C | - | -0.00969299 | 0.00175924 | 4.30E-08 | 534045 | -0.0069 | 0.0095 | 0.4689 | 484121 | TRUE | TRUE | FALSE |
| 474 | rs236587 | C | T | - | -0.00843594 | 0.001399 | 9.30E-10 | 534045 | 0.0047 | 0.0087 | 0.592199 | 484121 | TRUE | TRUE | FALSE |
| 475 | rs238819 | C | T | - | 0.00954743 | 0.00159163 | 2.60E-09 | 534045 | 0.0162 | 0.01 | 0.1052 | 484121 | TRUE | TRUE | FALSE |
| 476 | rs2442497 | T | C | - | -0.0109625 | 0.00178319 | 1.40E-09 | 534045 | -0.0059 | 0.0114 | 0.6047 | 484121 | TRUE | TRUE | FALSE |
| 477 | rs251388 | T | C | - | -0.0095493 | 0.00121755 | 6.80E-15 | 534045 | -0.0032 | 0.0092 | 0.728501 | 484121 | TRUE | TRUE | FALSE |
| 478 | rs2592208 | A | C | - | -0.00657976 | 0.0012188 | 3.50E-08 | 534045 | -0.0016 | 0.0084 | 0.8451 | 484121 | TRUE | TRUE | FALSE |
| 479 | rs2615074 | A | G | - | 0.00966192 | 0.00125162 | 2.80E-15 | 534045 | 0.0052 | 0.0089 | 0.5592 | 484121 | TRUE | TRUE | FALSE |
| 480 | rs2627692 | T | C | - | -0.00987569 | 0.00121806 | 6.10E-17 | 534045 | -0.0016 | 0.0086 | 0.8534 | 484121 | TRUE | TRUE | FALSE |
| 481 | rs2749807 | T | A | - | 0.0076709 | 0.00129486 | 2.00E-10 | 534045 | 0.0024 | 0.0086 | 0.784099 | 484121 | TRUE | TRUE | FALSE |
| 482 | rs2756895 | T | C | - | 0.00759888 | 0.00121955 | 1.20E-09 | 534045 | 0.0099 | 0.0085 | 0.2433 | 484121 | TRUE | TRUE | FALSE |
| 483 | rs28445331 | C | A | - | 0.0074215 | 0.00125967 | 2.00E-09 | 534045 | -0.0066 | 0.0086 | 0.444 | 484121 | TRUE | TRUE | FALSE |
| 484 | rs28575622 | T | G | - | 0.0166054 | 0.00253745 | 1.40E-11 | 534045 | 0.0043 | 0.0178 | 0.8084 | 484121 | TRUE | TRUE | FALSE |
| 485 | rs29946 | C | T | - | 0.00774908 | 0.00124964 | 2.10E-09 | 534045 | 0.0143 | 0.0101 | 0.1558 | 484121 | TRUE | TRUE | FALSE |
| 486 | rs3013301 | A | G | - | -0.00685818 | 0.00127614 | 4.70E-08 | 534045 | -0.0048 | 0.0093 | 0.6078 | 484121 | TRUE | TRUE | FALSE |
| 487 | rs3116603 | C | G | - | -0.027381 | 0.00148378 | 1.30E-77 | 534045 | 0.0263 | 0.0149 | 0.0770105 | 484121 | TRUE | TRUE | FALSE |
| 488 | rs32736 | G | T | - | 0.00684865 | 0.0012795 | 4.00E-08 | 534045 | -0.0021 | 0.0095 | 0.8272 | 484121 | TRUE | TRUE | FALSE |
| 489 | rs34693680 | T | C | - | 0.0128338 | 0.00178742 | 1.40E-13 | 534045 | 0.0025 | 0.0183 | 0.8919 | 484121 | TRUE | TRUE | FALSE |
| 490 | rs34825238 | T | G | - | 0.00810291 | 0.00141182 | 1.10E-08 | 534045 | -0.0212 | 0.0111 | 0.0558599 | 484121 | TRUE | TRUE | FALSE |
| 491 | rs35050648 | T | G | - | 0.00907837 | 0.00143679 | 1.90E-10 | 534045 | -0.0089 | 0.0117 | 0.4478 | 484121 | TRUE | TRUE | FALSE |
| 492 | rs35446432 | T | C | - | 0.00711527 | 0.00129235 | 1.90E-08 | 534045 | 0.0087 | 0.0092 | 0.3401 | 484121 | TRUE | TRUE | FALSE |
| 493 | rs35492502 | A | G | - | 0.00967173 | 0.00132361 | 2.50E-13 | 534045 | -0.0056 | 0.0114 | 0.625 | 484121 | TRUE | TRUE | FALSE |
| 494 | rs359938 | G | A | - | -0.0092321 | 0.00143701 | 1.70E-10 | 534045 | 0.0193 | 0.0136 | 0.1559 | 484121 | TRUE | TRUE | FALSE |
| 495 | rs36073309 | A | G | - | 0.00832163 | 0.00129455 | 1.50E-10 | 534045 | 0.0052 | 0.0099 | 0.6016 | 484121 | TRUE | TRUE | FALSE |
| 496 | rs36089326 | T | A | - | 0.00736535 | 0.00129415 | 7.80E-09 | 534045 | 0.006 | 0.012 | 0.615399 | 484121 | TRUE | TRUE | FALSE |
| 497 | rs3772051 | A | G | - | -0.00819723 | 0.00144342 | 8.80E-09 | 534045 | -4.00E-04 | 0.0126 | 0.9759 | 484121 | TRUE | TRUE | FALSE |
| 498 | rs3778157 | C | T | - | 0.0110266 | 0.00162305 | 4.20E-12 | 534045 | 0.0335 | 0.0156 | 0.0315 | 484121 | TRUE | TRUE | FALSE |
| 499 | rs3808424 | C | T | - | -0.0156684 | 0.00147209 | 4.10E-26 | 534045 | -4.00E-04 | 0.0087 | 0.9638 | 484121 | TRUE | TRUE | FALSE |
| 500 | rs3845344 | T | C | - | 0.0091788 | 0.00123617 | 3.50E-14 | 534045 | 0.0061 | 0.009 | 0.4993 | 484121 | TRUE | TRUE | FALSE |
| 501 | rs40393 | C | T | - | 0.00769746 | 0.00124844 | 1.60E-09 | 534045 | 0.0059 | 0.0096 | 0.541 | 484121 | TRUE | TRUE | FALSE |
| 502 | rs4235838 | A | G | - | -0.00834798 | 0.00129969 | 2.20E-10 | 534045 | 0.003 | 0.0087 | 0.7309 | 484121 | TRUE | TRUE | FALSE |
| 503 | rs4257594 | T | C | - | -0.00726035 | 0.00125748 | 2.60E-08 | 534045 | 2.00E-04 | 0.009 | 0.9848 | 484121 | TRUE | TRUE | FALSE |
| 504 | rs4291242 | C | T | - | 0.00829704 | 0.00145756 | 5.10E-09 | 534045 | 0.0318 | 0.0097 | 0.000982811 | 484121 | TRUE | TRUE | FALSE |
| 505 | rs4439140 | A | G | - | -0.00972751 | 0.00126402 | 6.10E-15 | 534045 | 0.0152 | 0.0087 | 0.0793999 | 484121 | TRUE | TRUE | FALSE |
| 506 | rs4525525 | T | G | - | -0.0106369 | 0.00140076 | 1.30E-14 | 534045 | -0.0031 | 0.0096 | 0.743401 | 484121 | TRUE | TRUE | FALSE |
| 507 | rs4566595 | A | C | - | 0.00727039 | 0.0012622 | 4.30E-09 | 534045 | 0.0021 | 0.0091 | 0.8156 | 484121 | TRUE | TRUE | FALSE |
| 508 | rs4648626 | A | C | - | 0.00789919 | 0.00121348 | 5.40E-11 | 534045 | -0.0164 | 0.0086 | 0.0574698 | 484121 | TRUE | TRUE | FALSE |
| 509 | rs4676442 | C | T | - | 0.00746662 | 0.00126062 | 3.90E-10 | 534045 | 0.0093 | 0.0085 | 0.273 | 484121 | TRUE | TRUE | FALSE |
| 510 | rs4727295 | A | G | - | -0.0170036 | 0.0016162 | 2.20E-26 | 534045 | 0.014 | 0.0173 | 0.4192 | 484121 | TRUE | TRUE | FALSE |
| 511 | rs4795318 | T | C | - | 0.00863275 | 0.0012214 | 2.60E-12 | 534045 | -0.0174 | 0.0092 | 0.0566905 | 484121 | TRUE | TRUE | FALSE |
| 512 | rs4800670 | C | G | - | -0.00691881 | 0.0012578 | 3.00E-08 | 534045 | 0.002 | 0.009 | 0.8211 | 484121 | TRUE | TRUE | FALSE |
| 513 | rs4912537 | T | C | - | -0.0125205 | 0.00142205 | 5.70E-19 | 534045 | -0.0129 | 0.0104 | 0.2118 | 484121 | TRUE | TRUE | FALSE |
| 514 | rs4912905 | C | G | - | -0.0100783 | 0.00140773 | 7.60E-13 | 534045 | 0.0053 | 0.0091 | 0.5577 | 484121 | TRUE | TRUE | FALSE |
| 515 | rs4948293 | A | G | - | -0.0105384 | 0.00150107 | 1.10E-12 | 534045 | 0.0095 | 0.0104 | 0.361 | 484121 | TRUE | TRUE | FALSE |
| 516 | rs5019542 | T | C | - | -0.00894166 | 0.00126069 | 1.20E-13 | 534045 | -0.0055 | 0.0095 | 0.5639 | 484121 | TRUE | TRUE | FALSE |
| 517 | rs528378 | C | A | - | 0.00705542 | 0.00126092 | 1.40E-08 | 534045 | 0.0187 | 0.0138 | 0.1728 | 484121 | TRUE | TRUE | FALSE |
| 518 | rs532499 | C | T | - | -0.00863852 | 0.00139547 | 4.70E-10 | 534045 | 0.0044 | 0.0116 | 0.701501 | 484121 | TRUE | TRUE | FALSE |
| 519 | rs55674305 | A | G | - | -0.00964482 | 0.00132353 | 1.20E-13 | 534045 | -0.0071 | 0.0108 | 0.510199 | 484121 | TRUE | TRUE | FALSE |
| 520 | rs55800172 | A | G | - | 0.0189177 | 0.00247115 | 2.20E-14 | 534045 | -0.0486 | 0.0311 | 0.1178 | 484121 | TRUE | TRUE | FALSE |
| 521 | rs55854145 | C | A | - | -0.0172474 | 0.00269612 | 4.80E-11 | 534045 | -0.0125 | 0.0322 | 0.6967 | 484121 | TRUE | TRUE | FALSE |
| 522 | rs56130943 | C | A | - | 0.00940205 | 0.00148129 | 3.00E-10 | 534045 | 0.0078 | 0.0167 | 0.6404 | 484121 | TRUE | TRUE | FALSE |
| 523 | rs56207600 | A | G | - | 0.0136817 | 0.00194733 | 4.70E-12 | 534045 | 0.0206 | 0.0132 | 0.1172 | 484121 | TRUE | TRUE | FALSE |
| 524 | rs56304624 | A | G | - | -0.00676137 | 0.00124345 | 2.50E-08 | 534045 | -0.007 | 0.0087 | 0.4207 | 484121 | TRUE | TRUE | FALSE |
| 525 | rs57135834 | T | C | - | -0.0160957 | 0.00141391 | 1.20E-29 | 534045 | -0.0235 | 0.0104 | 0.0246502 | 484121 | TRUE | TRUE | FALSE |
| 526 | rs599004 | T | C | - | -0.0101078 | 0.00134756 | 2.40E-14 | 534045 | -0.0165 | 0.0095 | 0.0816394 | 484121 | TRUE | TRUE | FALSE |
| 527 | rs60071805 | A | G | - | 0.00746956 | 0.00130594 | 3.80E-08 | 534045 | 0.0088 | 0.0107 | 0.4104 | 484121 | TRUE | TRUE | FALSE |
| 528 | rs6056342 | A | G | - | -0.00767753 | 0.00129089 | 7.50E-10 | 534045 | -0.0101 | 0.0101 | 0.3166 | 484121 | TRUE | TRUE | FALSE |
| 529 | rs60667771 | C | T | - | 0.0104829 | 0.00165495 | 1.80E-10 | 534045 | 0.0094 | 0.0099 | 0.3405 | 484121 | TRUE | TRUE | FALSE |
| 530 | rs6142059 | C | T | - | 0.00973613 | 0.0012236 | 1.70E-17 | 534045 | 0.0129 | 0.0085 | 0.1272 | 484121 | TRUE | TRUE | FALSE |
| 531 | rs61628776 | G | A | - | -0.0145523 | 0.00175353 | 3.90E-17 | 534045 | 0.0057 | 0.011 | 0.6034 | 484121 | TRUE | TRUE | FALSE |
| 532 | rs61775433 | G | A | - | 0.0131892 | 0.00158491 | 2.90E-17 | 534045 | -0.0162 | 0.0125 | 0.1953 | 484121 | TRUE | TRUE | FALSE |
| 533 | rs62275882 | A | G | - | -0.0107818 | 0.00174749 | 4.30E-10 | 534045 | 0.0053 | 0.0201 | 0.7935 | 484121 | TRUE | TRUE | FALSE |
| 534 | rs632224 | G | A | - | -0.0120826 | 0.00123139 | 1.30E-22 | 534045 | -0.013 | 0.0085 | 0.1246 | 484121 | TRUE | TRUE | FALSE |
| 535 | rs6493780 | G | A | - | -0.0134594 | 0.00212751 | 2.30E-10 | 534045 | -0.0337 | 0.0232 | 0.1474 | 484121 | TRUE | TRUE | FALSE |
| 536 | rs6563808 | C | T | - | -0.00992402 | 0.00138759 | 1.10E-12 | 534045 | 0.007 | 0.009 | 0.4368 | 484121 | TRUE | TRUE | FALSE |
| 537 | rs6693481 | C | T | - | -0.00787115 | 0.00131386 | 2.70E-09 | 534045 | -0.0077 | 0.0087 | 0.3762 | 484121 | TRUE | TRUE | FALSE |
| 538 | rs67362530 | A | G | - | -0.0154582 | 0.00190473 | 1.80E-17 | 534045 | 0.0018 | 0.0172 | 0.919 | 484121 | TRUE | TRUE | FALSE |
| 539 | rs6745626 | T | C | - | 0.0090194 | 0.00122922 | 1.90E-14 | 534045 | -4.00E-04 | 0.0088 | 0.9617 | 484121 | TRUE | TRUE | FALSE |
| 540 | rs6772164 | A | C | - | 0.00897332 | 0.00126491 | 1.60E-12 | 534045 | -0.0044 | 0.0145 | 0.7604 | 484121 | TRUE | TRUE | FALSE |
| 541 | rs6779752 | A | G | - | -0.0131839 | 0.0012699 | 1.10E-25 | 534045 | 0.0087 | 0.0106 | 0.4105 | 484121 | TRUE | TRUE | FALSE |
| 542 | rs6905095 | A | C | - | -0.00816381 | 0.00122372 | 7.70E-12 | 534045 | -0.0063 | 0.0084 | 0.453799 | 484121 | TRUE | TRUE | FALSE |
| 543 | rs695922 | G | A | - | -0.00977492 | 0.00165769 | 1.20E-09 | 534045 | 0.0185 | 0.0108 | 0.0853493 | 484121 | TRUE | TRUE | FALSE |
| 544 | rs6975015 | A | G | - | 0.0168627 | 0.00189534 | 2.70E-19 | 534045 | -0.0305 | 0.0217 | 0.1603 | 484121 | TRUE | TRUE | FALSE |
| 545 | rs71423263 | G | T | - | 0.0131324 | 0.0017342 | 6.60E-15 | 534045 | 0.007 | 0.0131 | 0.594101 | 484121 | TRUE | TRUE | FALSE |
| 546 | rs7188009 | A | G | - | 0.00841701 | 0.0012411 | 1.20E-11 | 534045 | -0.0269 | 0.0146 | 0.0644006 | 484121 | TRUE | TRUE | FALSE |
| 547 | rs7245985 | G | T | - | -0.011072 | 0.00150737 | 3.00E-13 | 534045 | -0.0129 | 0.0117 | 0.2722 | 484121 | TRUE | TRUE | FALSE |
| 548 | rs7269113 | C | T | - | -0.00863801 | 0.00155122 | 1.10E-08 | 534045 | 0.0122 | 0.0174 | 0.4848 | 484121 | TRUE | TRUE | FALSE |
| 549 | rs72697614 | A | C | - | 0.00775289 | 0.00130993 | 9.90E-10 | 534045 | -0.0027 | 0.0101 | 0.787601 | 484121 | TRUE | TRUE | FALSE |
| 550 | rs72771080 | T | C | - | 0.0138091 | 0.00148901 | 3.50E-21 | 534045 | -0.0114 | 0.0157 | 0.4688 | 484121 | TRUE | TRUE | FALSE |
| 551 | rs72820209 | T | A | - | 0.00702928 | 0.00123674 | 7.40E-09 | 534045 | -0.0118 | 0.0135 | 0.3837 | 484121 | TRUE | TRUE | FALSE |
| 552 | rs72845395 | C | G | - | -0.0222242 | 0.00307003 | 2.20E-13 | 534045 | 0.0498 | 0.0349 | 0.1538 | 484121 | TRUE | TRUE | FALSE |
| 553 | rs7301341 | C | T | - | -0.00948523 | 0.00129534 | 1.20E-13 | 534045 | 0.0038 | 0.0087 | 0.66 | 484121 | TRUE | TRUE | FALSE |
| 554 | rs73081811 | G | T | - | 0.00878213 | 0.00121732 | 2.60E-13 | 534045 | -0.0105 | 0.0109 | 0.332 | 484121 | TRUE | TRUE | FALSE |
| 555 | rs73093103 | T | A | - | 0.0241633 | 0.00335482 | 1.30E-13 | 534045 | -0.0024 | 0.038 | 0.9498 | 484121 | TRUE | TRUE | FALSE |
| 556 | rs7321045 | A | G | - | 0.00945976 | 0.00123758 | 2.50E-15 | 534045 | -0.0023 | 0.0084 | 0.7813 | 484121 | TRUE | TRUE | FALSE |
| 557 | rs73791415 | T | G | - | -0.00718243 | 0.00126585 | 2.40E-08 | 534045 | -0.0114 | 0.0107 | 0.2856 | 484121 | TRUE | TRUE | FALSE |
| 558 | rs7399309 | T | C | - | 0.0120441 | 0.00184542 | 3.50E-11 | 534045 | 0.0149 | 0.0101 | 0.1405 | 484121 | TRUE | TRUE | FALSE |
| 559 | rs74032128 | G | A | - | 0.0216905 | 0.00343987 | 3.40E-10 | 534045 | -0.056 | 0.0434 | 0.1968 | 484121 | TRUE | TRUE | FALSE |
| 560 | rs741409 | T | C | - | 0.0108485 | 0.00166966 | 9.60E-11 | 534045 | -0.0017 | 0.012 | 0.8898 | 484121 | TRUE | TRUE | FALSE |
| 561 | rs7426945 | G | A | - | 0.00750398 | 0.00121691 | 2.40E-09 | 534045 | 0.0058 | 0.0084 | 0.4943 | 484121 | TRUE | TRUE | FALSE |
| 562 | rs74737644 | G | A | - | -0.01409 | 0.00212978 | 7.70E-12 | 534045 | -0.0113 | 0.0211 | 0.590999 | 484121 | TRUE | TRUE | FALSE |
| 563 | rs7487292 | G | T | - | 0.0102479 | 0.00122966 | 2.60E-17 | 534045 | -0.0094 | 0.0085 | 0.2651 | 484121 | TRUE | TRUE | FALSE |
| 564 | rs751894 | T | C | - | 0.00917897 | 0.00151504 | 2.00E-10 | 534045 | 0.0121 | 0.0112 | 0.2789 | 484121 | TRUE | TRUE | FALSE |
| 565 | rs752070 | G | A | - | 0.0108737 | 0.0018214 | 5.80E-10 | 534045 | -0.0038 | 0.0114 | 0.740099 | 484121 | TRUE | TRUE | FALSE |
| 566 | rs7546249 | A | T | - | 0.0151577 | 0.00135446 | 8.60E-30 | 534045 | 0.0072 | 0.0092 | 0.4361 | 484121 | TRUE | TRUE | FALSE |
| 567 | rs7576796 | A | G | - | -0.00803553 | 0.00144742 | 4.20E-08 | 534045 | -0.0101 | 0.016 | 0.5271 | 484121 | TRUE | TRUE | FALSE |
| 568 | rs76067562 | T | G | - | 0.0139888 | 0.00207169 | 1.30E-11 | 534045 | 0.0271 | 0.0247 | 0.2725 | 484121 | TRUE | TRUE | FALSE |
| 569 | rs7671407 | A | G | - | -0.00755734 | 0.00135575 | 2.20E-08 | 534045 | -0.0018 | 0.01 | 0.8603 | 484121 | TRUE | TRUE | FALSE |
| 570 | rs76733024 | G | A | - | -0.01454 | 0.00245091 | 9.10E-10 | 534045 | -0.008 | 0.0234 | 0.732399 | 484121 | TRUE | TRUE | FALSE |
| 571 | rs7673764 | A | G | - | -0.0139224 | 0.00248308 | 1.30E-08 | 534045 | 0.0258 | 0.0209 | 0.2181 | 484121 | TRUE | TRUE | FALSE |
| 572 | rs7691068 | C | T | - | 0.00663792 | 0.00121378 | 4.60E-08 | 534045 | 0.0049 | 0.0092 | 0.5926 | 484121 | TRUE | TRUE | FALSE |
| 573 | rs76951439 | T | C | - | 0.0204589 | 0.00271149 | 3.20E-14 | 534045 | -0.023 | 0.0373 | 0.5386 | 484121 | TRUE | TRUE | FALSE |
| 574 | rs77093479 | G | C | - | -0.0111724 | 0.00165359 | 2.10E-11 | 534045 | -7.00E-04 | 0.0099 | 0.9423 | 484121 | TRUE | TRUE | FALSE |
| 575 | rs7719688 | G | A | - | -0.00878572 | 0.00123064 | 2.80E-13 | 534045 | -8.00E-04 | 0.0095 | 0.9317 | 484121 | TRUE | TRUE | FALSE |
| 576 | rs7755185 | G | A | - | 0.00879721 | 0.0013112 | 1.20E-11 | 534045 | 0.0123 | 0.0086 | 0.1546 | 484121 | TRUE | TRUE | FALSE |
| 577 | rs7758804 | T | C | - | 0.00741954 | 0.0012456 | 1.90E-08 | 534045 | -0.005 | 0.0097 | 0.6076 | 484121 | TRUE | TRUE | FALSE |
| 578 | rs7776917 | A | G | - | 0.0127745 | 0.00121941 | 4.60E-26 | 534045 | 1.00E-04 | 0.0087 | 0.9944 | 484121 | TRUE | TRUE | FALSE |
| 579 | rs781648 | T | C | - | -0.0139006 | 0.0024113 | 9.70E-09 | 534045 | 0.0407 | 0.0282 | 0.1488 | 484121 | TRUE | TRUE | FALSE |
| 580 | rs7843128 | C | T | - | -0.00777863 | 0.00127149 | 5.00E-09 | 534045 | -0.0091 | 0.0089 | 0.3032 | 484121 | TRUE | TRUE | FALSE |
| 581 | rs7900548 | G | T | - | -0.0138869 | 0.00160846 | 1.40E-17 | 534045 | 0.033 | 0.0118 | 0.00518203 | 484121 | TRUE | TRUE | FALSE |
| 582 | rs8017780 | A | C | - | -0.00926013 | 0.00149837 | 3.40E-10 | 534045 | -0.0132 | 0.0117 | 0.2564 | 484121 | TRUE | TRUE | FALSE |
| 583 | rs80295797 | T | C | - | -0.0120873 | 0.00128938 | 2.20E-21 | 534045 | 0.0091 | 0.0096 | 0.3433 | 484121 | TRUE | TRUE | FALSE |
| 584 | rs8091374 | A | G | - | -0.0101354 | 0.00169263 | 1.30E-09 | 534045 | -0.0095 | 0.0108 | 0.3774 | 484121 | TRUE | TRUE | FALSE |
| 585 | rs836532 | A | G | - | 0.0110783 | 0.00151656 | 4.80E-14 | 534045 | 0.0475 | 0.0245 | 0.0525896 | 484121 | TRUE | TRUE | FALSE |
| 586 | rs878347 | C | T | - | -0.00912659 | 0.00126373 | 4.20E-13 | 534045 | -0.0027 | 0.0086 | 0.756201 | 484121 | TRUE | TRUE | FALSE |
| 587 | rs882378 | C | A | - | 0.00992979 | 0.00132012 | 1.00E-14 | 534045 | -0.0065 | 0.0213 | 0.759899 | 484121 | TRUE | TRUE | FALSE |
| 588 | rs903162 | A | G | - | 0.00752678 | 0.00131174 | 8.40E-09 | 534045 | 0.0035 | 0.0093 | 0.7059 | 484121 | TRUE | TRUE | FALSE |
| 589 | rs9291926 | G | T | - | -0.0118167 | 0.0012162 | 2.50E-22 | 534045 | 0.0081 | 0.0085 | 0.3409 | 484121 | TRUE | TRUE | FALSE |
| 590 | rs9399656 | T | C | - | -0.00838239 | 0.0012173 | 7.40E-12 | 534045 | -0.0061 | 0.0102 | 0.5481 | 484121 | TRUE | TRUE | FALSE |
| 591 | rs946197 | C | G | - | 0.0137795 | 0.00143529 | 3.00E-21 | 534045 | 0.0068 | 0.0091 | 0.4553 | 484121 | TRUE | TRUE | FALSE |
| 592 | rs9480933 | G | C | - | -0.012177 | 0.00127221 | 1.40E-22 | 534045 | -0.0081 | 0.0085 | 0.3387 | 484121 | TRUE | TRUE | FALSE |
| 593 | rs987204 | A | G | - | 0.0103734 | 0.00122123 | 1.70E-17 | 534045 | -0.0094 | 0.0085 | 0.2684 | 484121 | TRUE | TRUE | FALSE |
| 594 | rs9951619 | G | T | - | 0.0118305 | 0.0014549 | 2.40E-17 | 534045 | 0.0053 | 0.011 | 0.629901 | 484121 | TRUE | TRUE | FALSE |
| 595 | rs9967287 | T | G | - | 0.00774088 | 0.00140914 | 3.00E-08 | 534045 | 0.0246 | 0.0088 | 0.00526405 | 484121 | TRUE | TRUE | FALSE |
| 596 | rs9985795 | C | T | - | -0.0071301 | 0.00121915 | 4.10E-09 | 534045 | -0.0146 | 0.0136 | 0.2836 | 484121 | TRUE | TRUE | FALSE |
| 597 | rs1795323 | G | T | - | 0.00636662 | 0.00121506 | 4.00E-08 | 534045 | 0.0045 | 0.009 | 0.6131 | 484121 | TRUE | TRUE | FALSE |
| 598 | rs926436 | A | G | - | 0.00916139 | 0.00173375 | 4.20E-08 | 534045 | 0.0126 | 0.0106 | 0.2339 | 484121 | TRUE | TRUE | FALSE |
| Abbreviations: BMR, Basal Metabolic Rate; IS, Ischemic Stroke; EA,effect allele; OA, Other allele;Beta,the effect size of the SNP;SE,standard error;EXP,exposure;out,outcome;rs,reference single-nucleotide polymorphism; SNP,single-nucleotide polymorphism; palindromic+ambiguous, TRUE means SNP was not palindromic or ambiguous; non-confounders,TRUE means SNP was not related to confounding factors; non-outliers, TRUE means SNP was not outliers. | | | | | | | | | | | | | | | |
