# Supplementary material for: The association between basal metabolic rate and ischemic stroke: a Mendelian randomization study
Source: Front Neurol. 2025 Mar 3;16:1434740. doi: 10.3389/fneur.2025.1434740 (PMC11912940; doi:10.3389/fneur.2025.1434740)
Supplement: Supplementary file 7 [file Table_4.DOCX]

| **Supplementary Table 4 MR Analysis of IS and BMR Indicators** | | | | | |
| --- | --- | --- | --- | --- | --- |
| Outcome | Method | BMR | | |  |
|  |  | Number of SNPs | SE | OR(95%CI) | p Value |
| IS  (Before  MR-PRESSO) | IVW | 522 | 0.041 | 1.090(1.004–1.182) | 3.962E-02 |
|  | MR-Egger | 522 | 0.112 | 1.139(0.915-1.418) | 2.454E-01 |
|  | WME | 522 | 0.063 | 1.151(1.018-1.301) | 2.443E-02 |
|  | Simple mode | 522 | 0.185 | 1.175(0.817-1.690) | 3.841E-01 |
|  | Weighted mode | 522 | 0.149 | 1.218(0.909-1.632) | 1.867E-01 |
| IS  (After  MR-PRESSO) | IVW | 288 | 0.050 | 1.108(1.005-1.221) | 3.925E-02 |
|  | MR-Egger | 288 | 0.129 | 1.291(1.002-1.663) | 4.910E-02 |
|  | WME | 288 | 0.074 | 1.179(1.020-1.363) | 2.631E-02 |
|  | Simple mode | 288 | 0.200 | 1.031(0.701-1.516) | 8.758E-01 |
|  | Weighted mode | 288 | 0.150 | 1.189(0.885-1.600) | 2.511E-01 |
| BMR, Basal Metabolic Rate; IS, Ischemic Stroke; MR, Mendelian randomization; SE, standard error; SNP, single nucleotide polymorphism; IVW, inverse variance weighting; WME, weighted median. | | | | | |
|  |  |  |  |  |  |
|  |  |  |  |  |  |
