# Supplementary material for: The association between basal metabolic rate and ischemic stroke: a Mendelian randomization study
Source: Front Neurol. 2025 Mar 3;16:1434740. doi: 10.3389/fneur.2025.1434740 (PMC11912940; doi:10.3389/fneur.2025.1434740)
Supplement: Supplementary file 8 [file Table_5.DOCX]

| **Supplementary Table 5 Assessing directional pleiotropy through MR-Egger intercept and MR-PRESSO test among univariable MR.** | | | | | | |
| --- | --- | --- | --- | --- | --- | --- |
| Exposure | Outcome | MR-egger intercept | | |  | MR-PRESSO |
|  |  | intercept | P-value | SE |  | Global test  Pval |
| BWM | IS | -0.002 | 0.201 | 0.002 |  | 0.135 |
| BMR,Basal Metabolic Rate; IS, Ischemic Stroke; P value, p-value for the genetic association; SE,standard error. | | | | | | |
|  |  |  |  |  |  |  |
|  |  |  |  |  |  |  |
