# Supplementary material for: The association between basal metabolic rate and ischemic stroke: a Mendelian randomization study
Source: Front Neurol. 2025 Mar 3;16:1434740. doi: 10.3389/fneur.2025.1434740 (PMC11912940; doi:10.3389/fneur.2025.1434740)
Supplement: Supplementary file 9 [file Table_6.DOCX]

| **Supplementary Table 6 Analysis of Cochran’s *Q* test for univariate Heterogeneity** | | | | | | | | |
| --- | --- | --- | --- | --- | --- | --- | --- | --- |
| Exposure | Outcome | MR-  PRESSO | IVW | | | MR-Egger | | |
|  |  |  | Cochran’s Q | Q_df | Q- P value | Cochran’s Q | Q_df | Q- P value |
| BMR | IS | Before  (522SNPs) | 598.842 | 521 | 0.01 | 598.632 | 520 | 0.36 |
|  |  | After  (288SNPs) | 313.293 | 287 | 0.14 | 311.501 | 286 | 0.14 |
| BMR,Basal Metabolic Rate; IS, Ischemic Stroke; IVW, inverse variance weighting; | | | | | | | | |
