# Supplementary material for: The association between basal metabolic rate and ischemic stroke: a Mendelian randomization study
Source: Front Neurol. 2025 Mar 3;16:1434740. doi: 10.3389/fneur.2025.1434740 (PMC11912940; doi:10.3389/fneur.2025.1434740)
Supplement: Supplementary file 10 [file Table_7.DOCX]

| **Supplementary Table 7 Detailed information of SNPs used in BMR and IS Rverse MR analyses** | | | | | | | | | | | |
| --- | --- | --- | --- | --- | --- | --- | --- | --- | --- | --- | --- |
| N | SNP | EA | OA | beta.exp | se.exp | pval.exp | samplesize.exp | beta.out | se.out | pval.out | samplesize.out |
| 1 | rs11045239 | A | G | 0.0604 | 0.0089 | 1.12E-11 | 484121 | 0.00438129 | 0.00124501 | 0.000179999 | 534045 |
| 2 | rs11065836 | A | G | -0.0608 | 0.0103 | 3.34E-09 | 484121 | 0.00378224 | 0.0027307 | 0.19 | 534045 |
| 3 | rs11105378 | T | C | -0.054 | 0.0098 | 4.11E-08 | 484121 | 0.00637889 | 0.00161837 | 9.70E-05 | 534045 |
| 4 | rs11191772 | C | T | -0.0511 | 0.0086 | 3.40E-09 | 484121 | -0.00196452 | 0.00124722 | 0.14 | 534045 |
| 5 | rs11880613 | A | G | -0.0613 | 0.011 | 2.58E-08 | 484121 | 0.00519816 | 0.00155976 | 0.000870001 | 534045 |
| 6 | rs12509595 | C | T | 0.0577 | 0.0091 | 2.56E-10 | 484121 | -0.000689217 | 0.00133824 | 0.649999 | 534045 |
| 7 | rs1275980 | T | C | -0.0582 | 0.0094 | 5.28E-10 | 484121 | -0.00640196 | 0.00124202 | 2.30E-07 | 534045 |
| 8 | rs13123551 | A | T | 0.0552 | 0.0097 | 1.30E-08 | 484121 | -7.26E-05 | 0.00122931 | 0.96 | 534045 |
| 9 | rs2501968 | G | A | -0.049 | 0.0086 | 1.10E-08 | 484121 | -0.000195801 | 0.00123658 | 0.630001 | 534045 |
| 10 | rs2526620 | G | A | 0.05 | 0.0092 | 4.57E-08 | 484121 | 0.0018119 | 0.00155535 | 0.26 | 534045 |
| 11 | rs284160 | A | G | 0.0572 | 0.0096 | 2.80E-09 | 484121 | -0.00155011 | 0.00215441 | 0.42 | 534045 |
| 12 | rs7091346 | T | C | -0.0582 | 0.0098 | 2.53E-09 | 484121 | -0.00148983 | 0.00126844 | 0.18 | 534045 |
| 13 | rs7451833 | G | A | 0.128 | 0.022 | 5.82E-09 | 484121 | -0.00512977 | 0.00206007 | 0.00669993 | 534045 |
| 14 | rs7859727 | T | C | 0.0569 | 0.0087 | 5.23E-11 | 484121 | 5.03E-05 | 0.00121936 | 0.86 | 534045 |
| Abbreviations: IS, Ischemic Stroke; BMR, Basal Metabolic Rate; EA,effect allele; OA, Other allele;Beta,the effect size of the SNP;SE,standard error;EXP,exposure;out,outcome;rs,reference single-nucleotide polymorphism; SNP,single-nucleotide polymorphism; | | | | | | | | | | | |
