# Supplementary material for: The association between basal metabolic rate and ischemic stroke: a Mendelian randomization study
Source: Front Neurol. 2025 Mar 3;16:1434740. doi: 10.3389/fneur.2025.1434740 (PMC11912940; doi:10.3389/fneur.2025.1434740)
Supplement: Supplementary file 11 [file Table_8.DOCX]

| **Supplementary Table 8 MR Analysis of IS and BMR Indicators** | | | | | |
| --- | --- | --- | --- | --- | --- |
| Outcome | Method | IS | | |  |
|  |  | Number of SNPs | SE | OR(95%CI) | p Value |
| BMR | IVW | 14 | 0.017 | 1.004(0.971–1.038) | 8.224E-01 |
|  | MR-Egger | 14 | 0.071 | 1.139(0.915-1.418) | 3.300E-01 |
|  | WME | 14 | 0.011 | 1.002(0.980-1.025) | 8.723E-01 |
|  | Simple mode | 14 | 0.021 | 1.004(0.964-1.046) | 8.400E-01 |
|  | Weighted mode | 14 | 0.187 | 1.001(0.965-1.039) | 9.529E-01 |
| BMR, Basal Metabolic Rate; IS, Ischemic Stroke; MR, Mendelian randomization; SE, standard error; SNP, single nucleotide polymorphism; IVW, inverse variance weighting; WME, weighted median. | | | | | |
|  |  |  |  |  |  |
|  |  |  |  |  |  |
